# Deuterated Cyclopropanation of Alkenes by Iron Catalysis

**DOI:** 10.1021/acs.orglett.5c05260

**Published:** 2026-01-26

**Authors:** Ilias Khan Rana, Khue N. M. Nguyen, Duong T. Ngo, David A. Nagib

**Affiliations:** Department of Chemistry and Biochemistry, 2647 The Ohio State University, Columbus, Ohio 43210, United States

## Abstract

Deuterium labeling is a key tool used in drug development to observe and prevent metabolism. Here, we report a mild and operationally simple protocol for the synthesis of deuterated cyclopropanes with high levels of deuterium incorporation. This Fe-catalyzed strategy uses dichloromethane-*d*_2_ for safe, practical, and diazo-free access to carbene reactivity. A sterically and electronically diverse range of alkenes with varying functional groups are tolerated in this deuterated cyclopropanation. This highly air and water tolerant method complements existing strategies and significantly broadens access to valuable deuterated cyclopropanes, including with applications for the late-stage functionalization of pharmaceuticals.

## Graphical abstract

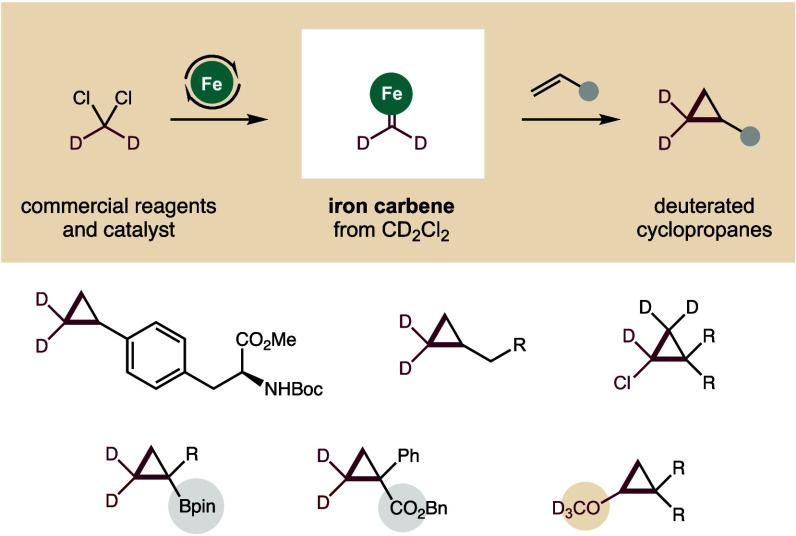

Deuterium labeling is a key tool used in drug discovery, as it enables the study and modulation of pharmacological activity.^1^ Specifically, the shorter, stronger, C–D bond slows metabolism, thus increasing stability and reducing dosage frequency.^2^ Deuterium incorporation can also reduce toxicity, increase half-life, and decrease drug–drug interactions.^3^ Likewise, deuterated drug analogs are commonly employed in ADME (absorption, distribution, metabolism and excretion) studies as standards for mass-based quantification of *in vivo* drug concentration.^4^ Several deuterated medicines are now in use, including deutetrabenazine, deucravacitinib, and deuruxolitinib (Figure 1a).^5^

**Figure 1:**
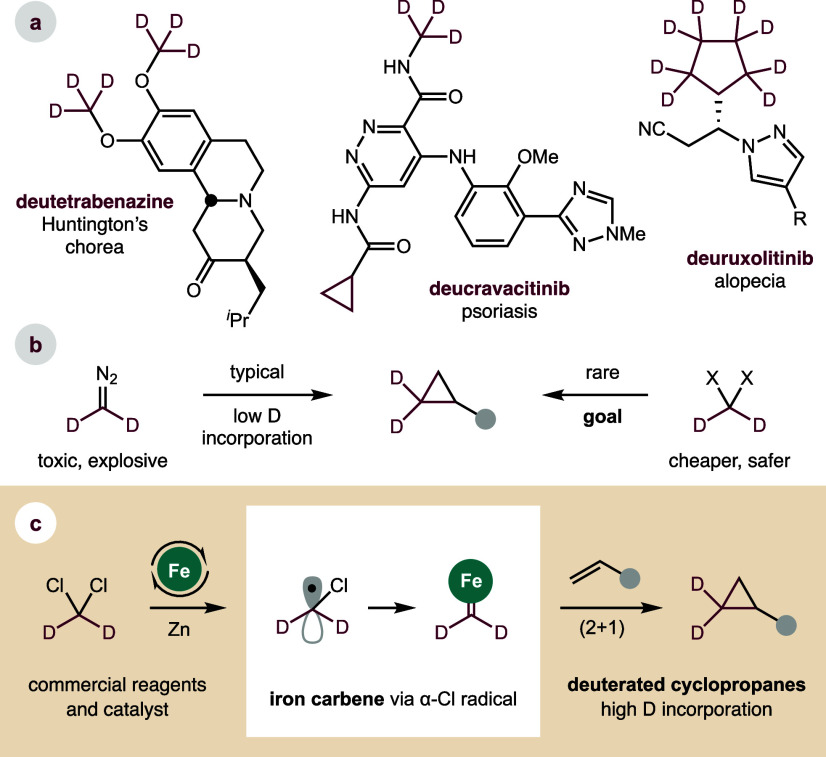
(a) Deuterium and cyclopropanes in medicine. Synthesis of deuterated cyclopropanes: (b) Current limitations. (c) Our Fe-catalyzed strategy.

Cyclopropanes are also privileged motifs in medicine, whose compact C­(*sp* ^3^)-rich architecture can similarly improve potency, increase metabolic stability, and reduce off-target effects, while boosting aqueous solubility and bioavailability.^6,7^ Yet, despite these many potential benefits, deuterated cyclopropanes remain rare in medicinal chemistry. Given the limited synthetic accessibility to this motif, we proposed development of a general and efficient method to deuterated cyclopropanes would significantly increase usage of these isotopically labeled small rings for mechanistic and pharmacological studies.

Deuterium labeled compounds are typically synthesized by hydrogen isotope exchange (HIE) of C­(*sp* ^2^)–H bonds by rare earth metal catalysts (typically Ir) with D_2_ gas.^8^ Recent advances now include H/D exchange of C­(*sp* ^3^)–H bonds by radical methods, as well as dehalogenative deuteration with D_2_O.^9,10^

Cyclopropanes are most rapidly synthesized by (2 + 1) cycloaddition – often with diazomethane.^11^ Yet, given the toxicity, volatility, and explosive nature of diazomethane, large scale, industrial use is discouraged,^12^ with preference to *in situ* generation.^13^ The synthesis and use of diazomethane-*d*_2_ to access deuterated cyclopropanes also remains underdeveloped, with major limitations including low fidelity of D-incorporation due to H/D exchange (Figure 1b).^14^

Modern cyclopropanation strategies^15^ have been developed employing dihalides,^16–21^ α-acyloxy halides,^22^ sulfones,^23^ carbonyls,^24,25^ redox active esters,^26^ or biradicals^27^ as carbene precursors. Notably, CH_2_Cl_2_ is an ideal methylene transfer reagent, with wide accessibility to its deuterated analog, CD_2_Cl_2_. Yet, few examples of cyclopropanation with dichloromethane-*d*_2_ are known. Pioneering examples include an enamine cyclopropanation by Yan (Ti catalyst),^28^ nucleophilic variants by Uyeda (Ni catalyst)^29^ and Pitre (vitamin B12),^30^ and a photocatalytic version by Lloret-Fillol (Ni cocatalyst).^31^

Inspired by these pioneering examples, we sought to develop a practical method for deuterated cyclopropanation that uses a commercially available Fe catalyst, mild Zn reductant, CD_2_Cl_2_, and is widely applicable to all alkene types (Figure 1c). We recently introduced a strategy that converts *gem*-dichlorides to carbenes by Fe catalysis, which we considered well-suited to address this challenge.^21^ In this approach, Zn (in the presence of LiI) reduces a dichloride to an α-Cl radical, which is trapped by an Fe catalyst. Upon α-Cl elimination, an Fe-carbene is generated and facilitates (2 + 1) cycloaddition with alkenes to access cyclopropanes. Since we have shown a variety of electronically diverse R groups are tolerated on the dichloride, we hoped dichloromethane-*d*_2_ may also serve as a suitable carbene precursor.

To our delight, deuterated cyclopropanes are indeed accessible by employing CD_2_Cl_2_ as a carbene precursor in this strategy. The optimized conditions, as shown in Table 1, were developed using the alkene, 1,1-diphenylethylene, to yield the nonvolatile cyclopropane **1**. When 5 mol % iron tetraphenylporphyrin chloride (FeTPPCl) is used as a catalyst, along with Zn as sacrificial reductant, and LiI as an additive to improve CD_2_Cl_2_ reduction, and all are stirred with the alkene in THF at 60 °C, then >99% of the cyclopropane product is obtained. Notably, >99% D-incorporation is observed – without the H/D exchange seen with diazomethane-*d*_2_.

**Table 1:**
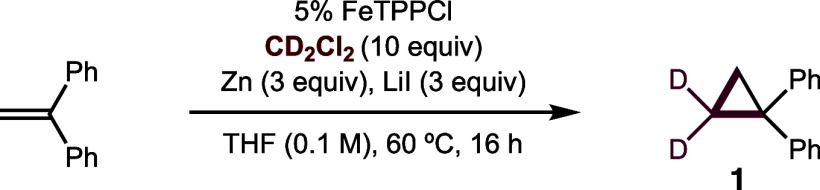
Fe-Catalyzed Cyclopropanation with CD_2_Cl_2_

entry	change from above^a^	yield **1**	D_2_ incorporation
**1**	**none**	**>99%**	**>20:1**
2	no FeTPPCl	0%	
3	no Zn or no LiI	0%	
4	NaI instead of LiI	90%	>20:1
5	LiF, LiClO_4_, LiBF_4_ instead of LiI	0%	
6	FeCl_2_, FeCl_3_ instead of FeTPPCl	<10%	>20:1
7	FeCl_3_ + TPP instead of FeTPPCl	99%	>20:1
8	Mn instead of Zn (±LiI)	24%	>20:1
9	+ H_2_O (10 equiv)	97%	>20:1
10	+ air (0.5 mL)	53%	>20:1
11	1 mmol scale	98%	>20:1

a 0.1 mmol alkene. Yields and D-incorporation determined by ^1^H NMR.

Important control reactions reveal that the Fe catalyst, Zn reductant, and LiI additive are all essential components for the transformation (entries 1–3). However, NaI may be used instead of LiI—with only a slight decrease in efficiency (entry 4, 90%). Yet, the iodide is necessary, as other Li salts (e.g., LiF, LiClO_4_, LiBF_4_) are not suitable replacements for LiI (entry 5). This suggests that halide exchange of CD_2_Cl_2_ to a more easily reduced iodide is likely operative.^21^ The porphyrin ligand is also necessary, as ligandless Fe salts (FeCl_2_, FeCl_3_) were ineffective (entry 6, <10%). Fortunately, efficient reactivity is restored with the addition of tetraphenylporphyrin (TPP) ligand to FeCl_3_ (entry 7, 99%). No precomplexation procedure is needed for this simple variation. Interestingly, replacing the mild reductant, Zn (−1.0 V; all reduction potentials versus SCE), with a stronger reductant, Mn (−1.4 V)^32^ is deleterious to the reaction (entry 8, 24%)—suggesting the rates of radical generation and capture are better synced in the Zn-mediated system. Notably, the cyclopropanation exhibits exceptional water tolerance (adding 10 equiv H_2_O retains 97% and >20:1 D-incorporation), suitable air tolerance (53%), and it was easily scaled to 1 mmol (98%)—demonstrating the robustness of this operationally simple protocol (entries 9–11).

With optimized conditions in hand, we then evaluated the scope and synthetic utility of this Fe-catalyzed deuterated cyclopropanation (Figure 2). Pleasingly, we found a sterically and electronically diverse range of alkenes were amenable to this carbene cycloaddition. For example, nucleophilic alkenes such as 1,1-disubstituted styrenes provide cyclopropanes in excellent yields (**1**–**6**). Interestingly, a fully deuterated cyclopropane-*d*_4_ (**2**) can be synthesized from the easily prepared *d*_2_-alkene. Electronically diverse alkenes are well-tolerated with electronically rich (**3**: *p*-OMe, σ_p_ = −0.2), neutral (**4**: *p*-F, σ_p_ = +0.1) and poor (**5**: *m*-CF_3_, σ_m_ = +0.4) styrenes all affording cyclopropanes in high yields (88–90%). Spirocyclic deuterated cyclopropane is also accessible, as in the fluorene scaffold (**6**).

**Figure 2:**
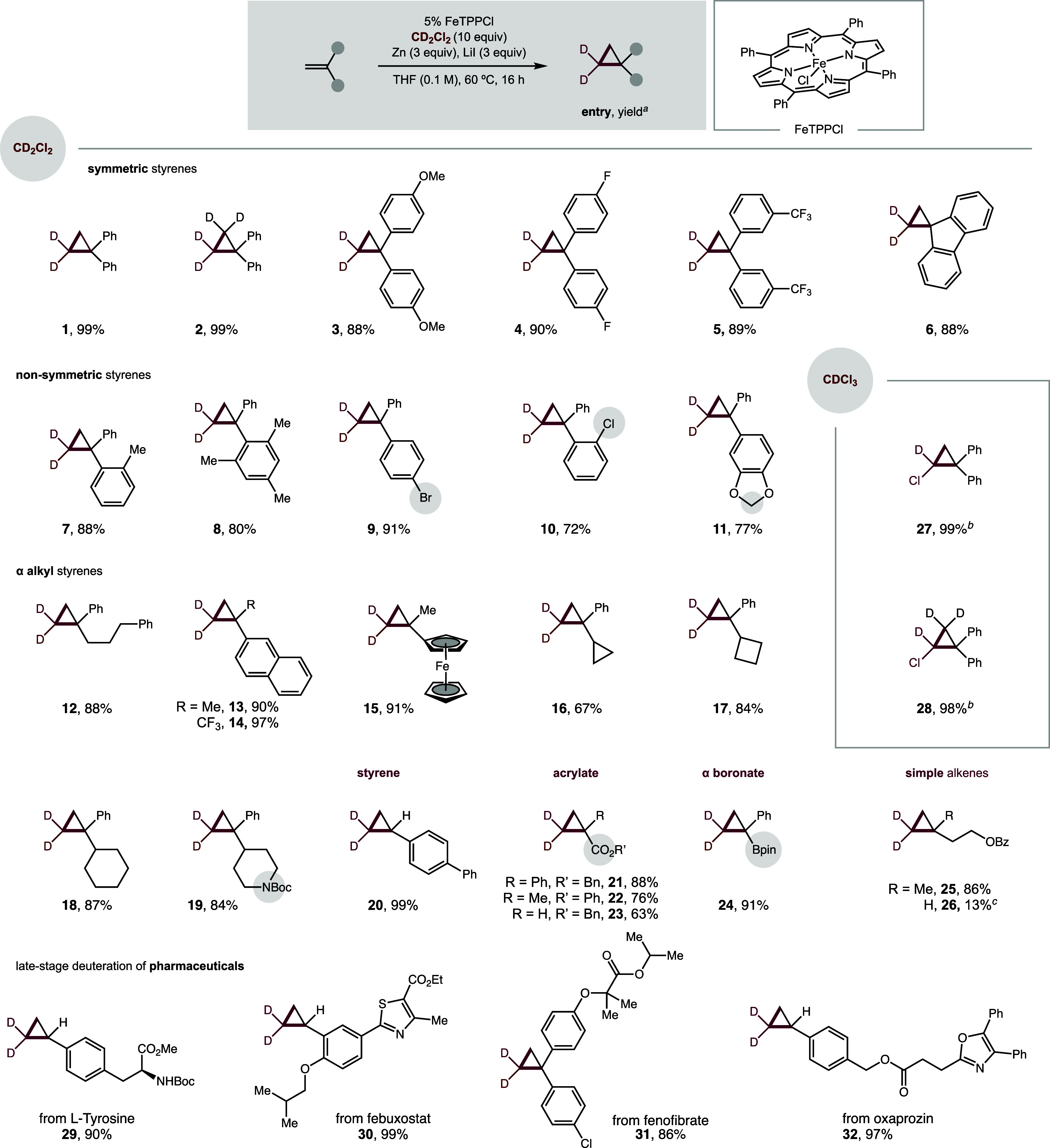
Evaluation of Fe-catalyzed deuterated cyclopropanation. ^*a*^Conditions: Alkene (0.2 mmol, 1 equiv), CD_2_Cl_2_ (10 equiv), FeTPPCl (5%), Zn (3 equiv), LiI (3 equiv), THF (1 mL), 60 °C, 16 h. ^*b*^CDCl_3_ (10 equiv) instead of CD_2_Cl_2_. ^*c*^Volatile product. Isolated yields. >99% D in all cases.

Steric bulk at the *ortho* positions are equally well-handled, as in the case of *o-*tolyl (**7**) and mesityl (**8**) substituents. Alkenes with halogen substituents, such as *p*-Br (**9**) or *o*-Cl, (**10**) react smoothly to provide cyclopropanes with cross-coupling handles. The carbene cycloaddition also tolerates activated C–H bonds (**11**) with no deleterious side reactivity.

Other alkenes—such as α-alkyl styrenes—are also amenable to this deuterated cyclopropanation. Phenyl (**12**), naphthyl (**13**–**14**) and ferrocenyl (**15**) arenes are tolerated. Aliphatic rings are also suitable substituents, including cyclopropane, cyclobutane, cyclohexane, and piperidine (**16**–**19**)—with the latter showcasing the most common heterocycle in pharmaceuticals.

Additional alkene classes that are amenable to this cyclopropanation include styrene (**20**), acrylates (**21**–**23**), and vinyl boronate (**24**)—with the latter suitable for additional diversification by cross-coupling. Moreover, simple alkenes are suitable, with 1,1-disubstitution (**25**) providing greater efficiency than terminal unactivated alkene (**26**). Notably, chloro-deuterated cyclopropanes (**27**, **28**) are also easily synthesized by using chloroform-*d* (CDCl_3_) instead of CD_2_Cl_2_ as the carbene precursor. This chlorinated handle provides a valuable synthetic handle for further cross-coupling. This product was previously only accessible by an electrochemical method.^33^

Having elucidated a broad tolerance of alkenes, we then probed utility of this deuterated cyclopropanation in late-stage functionalization of pharmaceuticals and other complex biologically relevant molecules. To this end, many alkenes were found to be well-tolerated, including those derived from l-tyrosine (**29**), febuxostat (**30**), fenofibrate (**31**), and oxaprozin (**32**).

Given the utility of deuterium in improving metabolic stability of the methoxy group (see deutetrabenazine, in Figure 1a), we wished to extend this strategy to an -OCD_3_ group. To our delight, when deuterated methoxy carbene precursor (prepared by deoxychlorination of methyl formate-*d*_3_ with oxalyl chloride) was employed, *d*_3_-methoxy cyclopropane **33** is readily obtained (eq 1; base added to counteract residual acid). We expect this Fe-catalyzed method for direct cyclopropanation with such a readily available dichloride will significantly improve access to drug derivatives with this important motif.



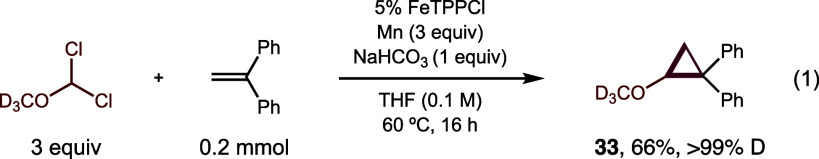

1


A mechanism for this Fe-catalyzed cyclopropanation is shown in Figure 3. In this proposal, *in situ* halide exchange of CD_2_Cl_2_ (**A**; −2.2 V)^21^ with LiI generates a more easily reducible iodide, ICDCl (**B**; −1.0 V).^21^ This intermediate is reduced by the mild reductant Zn (−1.4 V with LiI)^32^ to α-chloro radical **C** via single electron transfer (SET). Although additional halide exchange to CD_2_I_2_ (−0.7 V)^21^ cannot be excluded, it is unnecessary for SET. In parallel, the Fe­(III)­TPPCl precatalyst is reduced by Zn to Fe­(II)­TPP. This Fe­(II) porphyrin is known to rapidly capture chloromethyl radicals (2 × 10^9^ M^–1^ s^–1^).^34^ Thus, we expect α-chloro radical **C** is quickly captured by Fe­(II)­TPP to form organoiron­(III) **D**. Further reduction of **D** by Zn or Zn^+^ precedes by either: (1) SET to α-chloro Fe­(II), followed by α-chloride elimination, or (2) Cl· abstraction to α-radical Fe­(III). Either path provides the reactive Fe carbene **E**. Lastly, (2 + 1) cycloaddition of the deutero Fe carbene and alkene furnishes the cyclopropane and returns Fe­(II) to the catalytic cycle.

**Figure 3:**
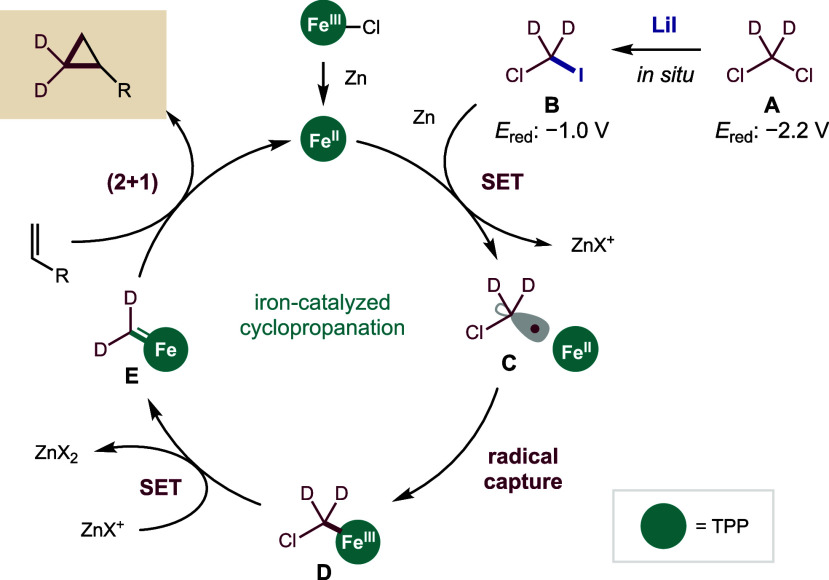
Mechanism of Fe-catalyzed cyclopropanation.

In conclusion, we have developed a mild, efficient, and robust method to access deuterated cyclopropanes from a diverse range of alkenes using CD_2_Cl_2_ or CDCl_3_ and a simple, commercially available, earth abundant iron catalyst. This iron carbene strategy provides high deuterium incorporation, has high air and water tolerance, and is compatible with many functional groups including halides and boronates, allowing further diversification by cross-coupling. Importantly, this operationally simple protocol provides ready access to deuterated cyclopropanes of complex and pharmaceutically relevant molecules. Thus, we expect its wide utility in the field of medicinal chemistry.

## Supplementary Material



## Data Availability

The data underlying this study are available in the published article and its Supporting Information.

## Notes

Detailed experimental procedures, characterization data, NMR spectra for all new compounds (PDF)
