# Supplementary material for: Deuterated Cyclopropanation of Alkenes by Iron Catalysis
Source: Org Lett. 2026 Jan 26;28(5):1750–4. doi: 10.1021/acs.orglett.5c05260 (PMC12888020; doi:10.1021/acs.orglett.5c05260)

Supplementary Material:

Deuterated Cyclopropanation of Alkenes by Iron Catalysis

Ilias Khan Rana, Khue N. M. Nguyen, Duong T. Ngo, David A. Nagib*

Department of Chemistry and Biochemistry, The Ohio State University
151 W Woodruff Ave., Columbus, OH, 43210, United States

Corresponding Author
Email: nagib.1@osu.edu

Table of Contents

Section	Pages
I. General Information	3
II. General Procedures	4-5
III. Preparation of Substrates	6-9
IV. Characterization of Products	10-26
V. References	27-28
VI. NMR Spectra	29-63

I. General Information

All chemicals and reagents were purchased from Sigma-Aldrich, Alfa Aesar, Acros, TCI, Oakwood, or Ambeed. CH_2Cl_2 and THF were dried and degassed with N_2 using an Innovative Technology solvent system. Silicycle F60 (230-400 mesh) silica gel was used for flash column chromatography. Thin layer chromatography (TLC) analyses were performed using Merck silica gel 60 F254 plates and visualized under UV (254 nm), $KMnO_4$, or iodine stain. ^{1}H, ^{19}F, ^{13}C NMR spectra were recorded using a Bruker AVIII 400 NMR spectrometer. ^{1}H NMR and ^{13}C NMR chemical shifts are reported in parts per million and referenced with respect to $CDCl_3$ (^{1}H: residual $CHCl_3$ at δ 7.26, ^{13}C: $CDCl_3$ triplet at δ 77.16). ^{1}H NMR data are reported as chemical shifts (δ ppm), multiplicity (s = singlet, bs = broad singlet, d = doublet, t = triplet, q = quartet, quint = quintet, sext = sextet, m = multiplet, app t = apparent triplet, app q = apparent quartet, app qd = apparent quartet of doublets), coupling constant (Hz), relative integral. ^{19}F NMR data are reported as chemical shifts (δ ppm). High resolution mass spectra were obtained using Thermo Fisher Scientific Orbitrap LC-MS (ESI).

II. General Procedures (GP)

GP1: Alkene Synthesis Via Wittig Reaction

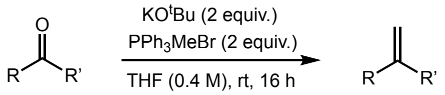

To an oven dried round bottom flask was added PPh_3MeBr (2 equiv.) and KO^{t}Bu (2 equiv.) under N_2 atmosphere. Dry THF (0.4 M) was added, and the resulting suspension was stirred at room temperature for 1 h. A solution of the ketone (1 equiv.) in THF was then added and the reaction mixture was stirred for 16 h at room temperature. After completion of the reaction, sat. NH_4Cl solution and deionized water were added, the organic layer was separated and the aqueous layer was extracted with Et_2O. The combined organic layers were dried with sodium sulfate, filtered, concentrated *in vacuo* and purified by silica gel flash column chromatography with appropriate eluents to afford the pure alkenes.

GP2: Alkene Synthesis Via Addition of Aryllithium to Acetophenone and Elimination

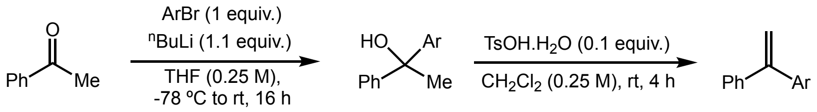

To a solution of the aryl bromide (1 equiv.) in dry THF (0.25 M) under N_2 atmosphere, ^{n}BuLi (1.1 equiv.) was added dropwise at -78 °C and the resulting solution was stirred for 2 h at the same temperature. Acetophenone (1.1 equiv.) was added dropwise to at -78 °C and the reaction mixture was allowed to warm up to room temperature and stirred overnight. After completion, the reaction was quenched with water (10 mL) and the organic layer was extracted with Et_2O (3 x 10 mL). The solvent was evaporated *in vacuo* and the resulting residue was dissolved in dry DCM (0.25 M). $TsOH.H_2O$ (10 mol%) was added and the reaction mixture was stirred at room temperature for 4 h. After TLC analysis indicated full conversion, the solvent was evaporated, and the crude mixture was purified by silica gel flash column chromatography to afford the pure alkenes.

GP3: Cyclopropanation Using CD_2Cl_2

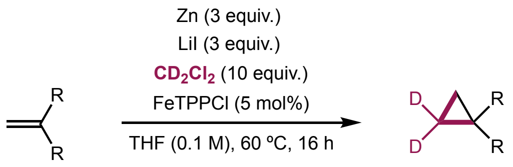

To an oven-dried 8 mL vial, Zn dust (3 equiv.), LiI (3 equiv.) FeTPPCl (5%), and a stir bar were added. The vial was sealed with a Teflon-lined septa cap, evacuated, and backfilled with N_2. Under N_2 atmosphere, THF (0.1 M) and CD_2Cl_2 (10 equiv.) were added, followed by the alkene (1 equiv.). The vial was then sealed with parafilm and electrical tape and the reaction mixture was allowed to stir at 60 °C overnight (stir rate: 800 rpm). After cooling to room temperature, the reaction was diluted with diethyl ether and filtered through a short pad of silica gel. The solvent was removed in vacuo and the crude mixture was purified by column chromatography on silica gel to afford the cyclopropanation product.

GP4: Cyclopropanation Using $D_3COCHCl_2$

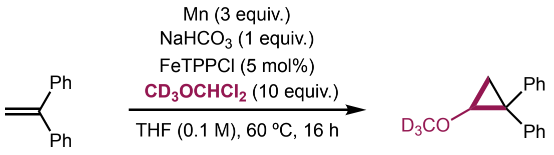

To an oven-dried 8 mL vial, Mn (3 equiv.), $NaHCO_3$ (1 equiv.) FeTPPCl (5%), and a stir bar were added. The vial was sealed with a Teflon-lined septa cap, evacuated, and backfilled with N_2. Under N_2 atmosphere, THF (0.1 M) and the alkene (1 equiv.) were added, followed by $D_3COCHCl_2$ (10 equiv.). The vial was then sealed with parafilm and electrical tape and the reaction mixture was allowed to stir at 60 °C overnight (stir rate: 800 rpm). After cooling to room temperature, the reaction was diluted with diethyl ether and filtered through a short pad of silica gel. The solvent was removed in vacuo and the crude mixture was purified by column chromatography on silica gel to afford the cyclopropanation product.

Notes:

i. In house synthesized carbene precursor, $D_3COCHCl_2$ contained HCl impurity which is detrimental to the cyclopropanation. It should be treated with $NaHCO_3$ (10 mg/mmol) for 15 mins (or until effervescence ceases) before the reaction to neutralize excess HCl. The precursor still remained significantly acidic but typically did not lower the cyclopropane yield as long as the aforementioned pretreatment was conducted.

ii. Excess $NaHCO_3$ in the cyclopropanation reaction should be avoided as 2 equiv. $NaHCO_3$ gave 25% of the product whereas 3 equiv. $NaHCO_3$ gave no product.

iii. For the protic version of the carbene precursor, i.e. $MeOCHCl_2$, commercial samples obtained from Oakwood Chemical showed much higher efficiency (>65% product) even without $NaHCO_3$ treatment than those obtained from Thermo Scientific (<5% product) which contain significantly higher amount of HCl.

III. Preparation of Substrates

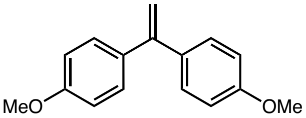

Prepared according to GP1 from 4,4’-dimethoxybenzophenone. Spectral data match the literature.[1]

4,4'-(ethene-1,1-diyl)bis(methoxybenzene)

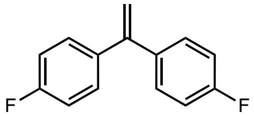

Prepared according to GP1 from 4,4’-difluorobenzophenone. Spectral data match the literature.[2]

4,4'-(ethene-1,1-diyl)bis(fluorobenzene)

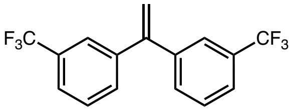

Prepared according to GP1 from 3,3’-bis(trifluoromethyl)benzophenone. Spectral data match the literature.[3]

3,3'-(ethene-1,1-diyl)bis((trifluoromethyl)benzene)

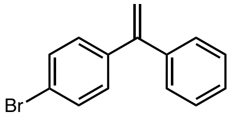

Prepared according to GP1 from 4-bromobenzophenone. Spectral data match the literature.[2]

1-bromo-4-(1-phenylvinyl)benzene

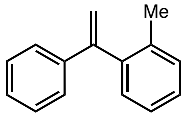

Prepared according to GP1 from 2-methylbenzophenone. Spectral data match the literature.[2]

1-methyl-2-(1-phenylvinyl)benzene

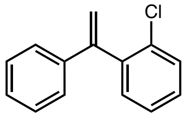

Prepared according to GP1 from 2-chlorobenzophenone. Spectral data match the literature.[2]

1-chloro-2-(1-phenylvinyl)benzene

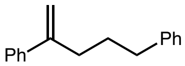

Prepared according to GP1 from 1,4-diphenylbutan-1-one. Spectral data match the literature.[4]

pent-4-ene-1,4-diyldibenzene

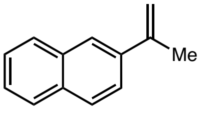

Prepared according to GP1 from 2-acetylnaphthalene. Spectral data match the literature.[1]

2-(prop-1-en-2-yl)naphthalene

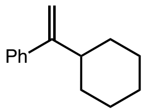

Prepared according to GP1 from cyclohexylphenylketone. Spectral data match the literature.[1]

(1-cyclohexylvinyl)benzene

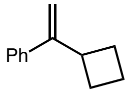

Prepared according to GP1 from cyclobutylphenylketone. Spectral data match the literature.[1]

(1-cyclobutylvinyl)benzene

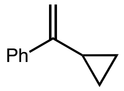

Prepared according to GP1 from cyclopropylphenylketone. Spectral data match the literature.[5]

(1-cyclopropylvinyl)benzene

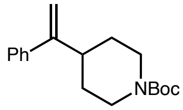

Prepared according to GP1 from tert-butyl 4-benzoylpiperidine-1-carboxylate. Spectral data match the literature.[6]

***tert*-butyl 4-(1-phenylvinyl)piperidine-1-carboxylate**

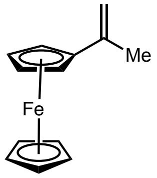

Prepared according to GP1 from acetylferrocene. Spectral data match the literature.[7]

2-(prop-1-en-2-yl)ferrocene

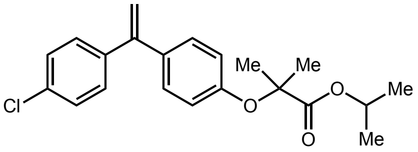

Prepared according to GP1 from fenofibrate. Spectral data match the literature.[8]

isopropyl 2-(4-(1-(4-chlorophenyl)vinyl)phenoxy)-2-methylpropanoate

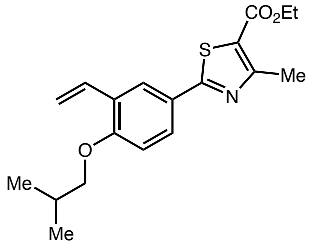

Prepared according to GP1 from the aldehyde. Spectral data match the literature.[9]

ethyl 2-(4-isobutoxy-3-vinylphenyl)-4-methylthiazole-5-carboxylate

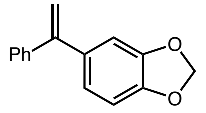

Prepared according to GP2 from 5-bromobenzo[*d*][1,3]dioxole and acetophenone. Spectral data match the literature.[10]

5-(1-phenylvinyl)benzo[*d*][1,3]dioxole

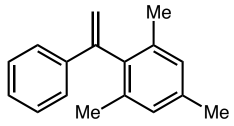

Prepared according to GP2 from 2-bromo-1,3,5-trimethylbenzene and acetophenone. Spectral data match the literature.[11]

1,3,5-trimethyl-2-(1-phenylvinyl)benzene

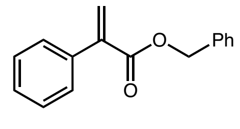

Prepared by Steglich esterification of phenylacrylic acid and benzyl alcohol. Spectral data match the literature.[12]

benzyl 2-phenylacrylate

Methyl (*S*)-2-((*tert*-butoxycarbonyl)amino)-3-(4-vinylphenyl)propanoate

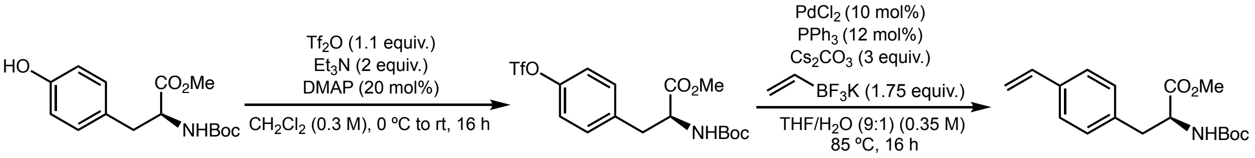

To a solution of the phenol (4 g, 13.5 mmol, 1 equiv.), Et_3N (3.84 mL, 2 equiv.) and DMAP (336 mg, 20 mol%) in CH_2Cl_2 (50 mL) at 0 °C was added Tf_2O (4.28 g, 1.1 equiv.) dropwise and the resulting mixture was allowed to warm up to room temperature and stirred for 16 h. After completion of the reaction, deionized water was added, the organic layer was separated and the aqueous layer was extracted with CH_2Cl_2 (3 x 20 mL). The combined organic layers were dried over sodium sulfate, filtered, concentrated *in vacuo* and purified by column chromatography (20% EtOAc/Hexane) to obtain the triflate as a white solid (5 g, 85%).

To a 20 mL vial equipped with a stirbar was added the triflate (1.5 g, 3.5 mmol, 1 equiv.), potassium vinyltrifluoroborate (820 mg, 1.75 equiv.), Cs_2CO_3 (3.42 g, 3 equiv.) and PPh_3 (110 mg, 12 mol%) followed by addition of $PdCl_2$ (62 mg, 10 mol%) in the glovebox. THF/H_2O (9:1) (10 mL) was added to the vial outside the glovebox and the reaction mixture was heated to 85 °C and stirred for 16 h. After completion, the reaction mixture was diluted with CH_2Cl_2 and filtered through a short pad of celite, concentrated *in vacuo* and purified by column chromatography to afford the alkene as a white solid (700 mg, 66%). Spectral data match the literature.[13]

4-vinylbenzyl 3-(4,5-diphenyloxazol-2-yl)propanoate

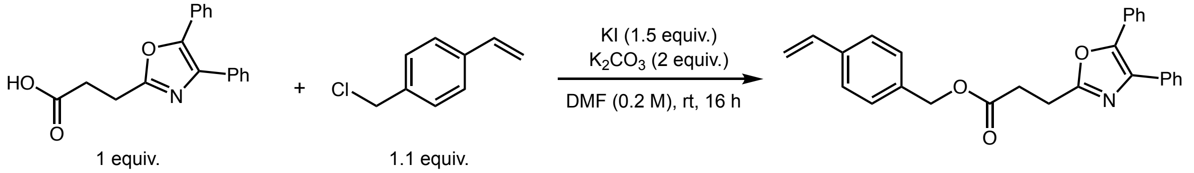

To a stirring solution of oxaprozin (2.35 g, 8 mmol, 1 equiv.) in dry DMF (40 mL) were sequentially added K_2CO_3 (2.2 g, 2 equiv.) and KI (2 g, 1.5 equiv.) at room temperature. To the stirring suspension, 4-

vinylbenzyl chloride (1.34 g, 1.1 equiv.) was added dropwise and the resulting mixture was stirred for 16 h. After completion, the reaction mixture was diluted with EtOAc followed by addition of deionized water. The organic layer was separated and washed with H_2O (3 x 50 mL) and brine (1 x 50 mL), dried with sodium sulfate, filtered and concentrated *in vacuo*. The crude mixture was purified by column chromatography (20% EtOAc/Hexane) to afford the alkene as a white solid (2.8 g, 86%). Spectral data match the literature.[14]

(ethene-1,1-diyl-2,2-d_2)dibenzene

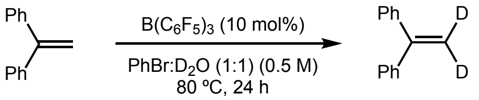

To an oven dried 8 mL vial equipped with a stir bar was added $B(C_6F_5)_3$ (51 mg, 10 mol%). The vial was sealed with a Teflon-lined septa cap, evacuated, and backfilled with N_2 three times, followed by addition of 1,1-diphenylethylene (180 mg, 1 mmol, 1 equiv.), PhBr (1 mL) and D_2O (1 mL). The vial was sealed with electrical tape and parafilm and the reaction mixture was heated to 80 °C and stirred for 24 h. After completion, the reaction was quenched with deionized water (4 mL) and extracted with EtOAc (3 x 10 mL). The combined organic layers were dried with sodium sulfate, filtered and concentrated *in vacuo*. The crude mixture was purified by column chromatography (100% Hexane) to afford the alkene as a colorless oil (135 mg, 76%). Spectral data match the literature.[15]

(dichloromethoxy)methane-d_3

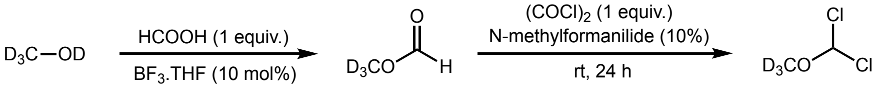

An oven dried 50 mL round bottom flask equipped with a stir bar was charged with CD_3OD (3.6 g, 100 mmol, 1 equiv.), HCOOH (4.6 g, 1 equiv.) and BF_3.THF (1 mL, 10 mol%). The flask was fitted with a distillation apparatus with a Vigreux column and a receiving flask cooled to 0 °C. The flask was heated to 60 °C in an oil bath with stirring and methyl formate-d_3 was collected as the distillate as a clear liquid (4 g, 67%).[16] bp 30 °C.

An oven dried 50 mL two-necked round bottom flask equipped with a stir bar and a water cooled condenser was charged with methyl formate-d_3 (3.78 g, 60 mmol, 1.5 equiv.) and N-methylformanilide (540 mg, 10 mol%). To the stirring solution, oxalyl chloride (3.4 mL, 1 equiv.) was added dropwise and the resulting mixture was stirred at room temperature for 24 h. After completion, the reaction mixture was subjected to distillation with a Vigreux column to obtain the gem-dichloride as a colorless liquid (2 g, 42%).[17] bp 65 – 75 °C

^{1}H NMR (400 MHz, $CDCl_3$): δ 7.33 (s, 1H).

^{13}C NMR (101 MHz, $CDCl_3$): δ 159.2, 98.9.

HRMS (ESI) m/z: $[M+H]^+$ Calcd. for $C_2H_2D_3Cl_2O$ 117.9906; Found 117.9905

IV. Characterization of Products

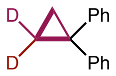

(cyclopropane-1,1-diyl-2,2-d_2)dibenzene (1)

Prepared according to **GP3** using diphenylethylene (0.2 mmol, 36 mg) and CD_2Cl_2 (2 mmol, 128 μL). The reaction mixture was purified by column chromatography (100% hexane) to provide the title compound **1** as a colorless oil (39 mg, 99%).

1 mmol scale up: To an oven-dried 20 mL vial, Zn dust (196 mg, 3 mmol, 3 equiv.), LiI (401 mg, 3 mmol, 3 equiv.), FeTPPCl (37.5 mg, 5 mol%), and a stir bar were added. The vial was sealed with a Teflon-lined septa cap, evacuated, and backfilled with N_2. Under N_2 atmosphere, THF (10 mL, 0.1 M) and CD_2Cl_2 (0.64 mL, 10 mmol, 10 equiv.) were added, followed by 1,1-diphenylethylene (180 mg, 1 equiv.). The vial was then sealed with parafilm and electrical tape and the reaction mixture was allowed to stir at 60 °C overnight (stir rate: 800 rpm). After cooling to room temperature, the reaction mixture was concentrated in vacuo and the crude mixture was purified by column chromatography (100% hexane) to afford the title compound **1** as a colorless oil (193 mg, 98%).

R_f = 0.55 (100% Hexane)

^{1}H NMR (400 MHz, $CDCl_3$): δ 7.34 – 7.17 (m, 10H), 1.30 (s, 2H).

^{13}C NMR (101 MHz, $CDCl_3$): δ 145.7, 128.4, 128.2, 125.9, 29.7, 16.2.

HRMS (ESI) m/z: $[M+H]^+$ Calcd. for $C_{15}H_{13}D_2$ 197.1299; Found 197.1286.

IR (neat) (cm^{-1}): 3023, 2925, 1599, 1494, 1451, 1020, 751, 693.

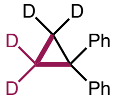

(cyclopropane-1,1-diyl-2,2,3,3-d_4)dibenzene (2)

Prepared according to **GP3** using diphenylethylene-d_2 (0.2 mmol, 36.4 mg) and CD_2Cl_2 (2 mmol, 128 μL). The reaction mixture was purified by column chromatography (100% hexane) to provide the title compound **2** as a colorless oil (39 mg, 99%).

R_f = 0.55 (100% Hexane)

^{1}H NMR (400 MHz, $CDCl_3$): δ 7.32 – 7.17 (m, 10H).

^{13}C NMR (101 MHz, $CDCl_3$): δ 145.7, 128.4, 128.2, 125.9, 29.5, 15.6.

HRMS (ESI) m/z: $[M+H]^+$ Calcd. for $C_{15}H_{11}D_4$ 199.1425; Found 199.1421.

IR (neat) (cm^{-1}): 3025, 2923, 1599, 1492, 1444, 1074, 778, 697.

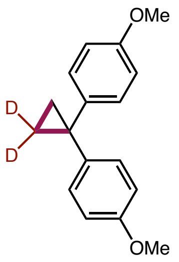

4,4'-(cyclopropane-1,1-diyl-2,2-d_2)bis(methoxybenzene) (3)

Prepared according to **GP3** using 4,4'-(ethene-1,1-diyl)bis(methoxybenzene) (0.2 mmol, 48 mg) and CD_2Cl_2 (2 mmol, 128 μL). The reaction mixture was purified by column chromatography (1% EtOAc/hexane) to provide the title compound **3** as a colorless oil (45 mg, 88%).

R*f* = 0.48 (5% EtOAc/Hexane)

^{1}H NMR (400 MHz, $CDCl_3$): δ 7.09 – 7.05 (m, 4H), 6.74 – 6.70 (m, 4H), 3.69 (s, 6H), 1.11 (s, 2H).

^{13}C NMR (101 MHz, $CDCl_3$): δ 157.7, 138.3, 129.3, 113.6, 55.3, 28.3, 15.9.

HRMS (ESI) m/z: $[M+H]^+$ Calcd. for $C_{17}H_{17}D_2O_2$ 257.1511; Found 257.1512.

IR (neat) (cm^{-1}): 3000, 2952, 1609, 1509, 1239, 1172, 1027, 823.

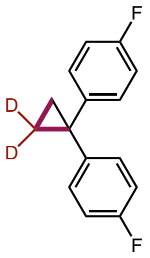

4,4'-(cyclopropane-1,1-diyl-2,2-d_2)bis(fluorobenzene) (4)

Prepared according to **GP3** using 4,4'-(ethene-1,1-diyl)bis(fluorobenzene) (0.2 mmol, 43.2 mg) and CD_2Cl_2 (2 mmol, 128 μL). The reaction mixture was purified by column chromatography (100% hexane) to provide the title compound **4** as a colorless oil (42 mg, 90%).

R*f* = 0.56 (100% Hexane)

^{1}H NMR (400 MHz, $CDCl_3$): δ 7.23 – 7.18 (m, 4H), 7.01 – 6.95 (m, 4H), 1.27 (s, 2H).

^{13}C NMR (101 MHz, $CDCl_3$): δ 162.4, 160.0, 141.2, 141.2, 129.8, 129.7, 115.1, 114.9, 28.6, 16.0.

^{19}F NMR (376 MHz, $CDCl_3$): δ -117.00.

HRMS (ESI) m/z: $[M+H]^+$ Calcd. for $C_{15}H_{11}D_2F_2$ 233.1111; Found 233.1107.

IR (neat) (cm^{-1}): 3042, 2928, 1603, 1507, 1217, 1163, 824, 548.

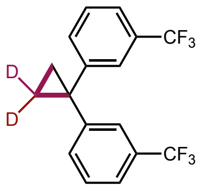

3,3'-(cyclopropane-1,1-diyl-2,2-*d*$_2$)bis((trifluoromethyl)benzene) (5)

Prepared according to **GP3** using 3,3'-(ethene-1,1-diyl)bis((trifluoromethyl)benzene) (0.2 mmol, 63.2 mg) and CD_2Cl_2 (2 mmol, 128 μL). The reaction mixture was purified by column chromatography (100% hexane) to provide the title compound **5** as a colorless oil (59 mg, 89%).

R*$_f$* = 0.57 (100% Hexane)

^{1}H NMR (400 MHz, $CDCl_3$): δ 7.41 – 7.37 (m, 4H), 7.34 – 7.30 (m, 4H), 1.29 (s, 2H).

^{13}C NMR (101 MHz, $CDCl_3$): δ 145.7, 132.0, 131.3, 131.0, 130.7, 130.4, 128.9, 128.1, 125.4, 124.8, 124.8, 124.8, 124.7, 123.3, 123.2, 123.2, 123.2, 122.7, 29.6, 16.3.

^{19}F NMR (376 MHz, $CDCl_3$): δ -62.60.

HRMS (ESI) m/z: $[M+Li]^+$ Calcd. for $C_{17}H_{10}D_2F_6Li$ 339.1129; Found 339.1116.

IR (neat) (cm^{-1}): 3076, 2966, 1490, 1435, 1332, 1117, 799, 698.

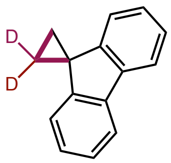

spiro[cyclopropane-1,9'-fluorene]-2,2-*d*$_2$ (6)

Prepared according to **GP3** using 9-methylene-9*H*-fluorene (0.2 mmol, 35.6 mg) and CD_2Cl_2 (2 mmol, 128 μL). The reaction mixture was purified by column chromatography (100% hexane) to provide the title compound **6** as an off white solid (34 mg, 88%).

R*$_f$* = 0.50 (100% Hexane)

^{1}H NMR (400 MHz, CDCl$_3$): δ 7.75 (dt, *J* = 7.6, 1.0 Hz, 2H), 7.30 – 7.18 (m, 4H), 6.96 (dt, *J* = 7.4, 1.0 Hz, 2H), 1.62 (s, 2H).

^{13}C NMR (101 MHz, CDCl$_3$): δ 148.1, 139.8, 126.8, 125.9, 120.0, 118.6, 29.3, 18.1.

HRMS (ESI) m/z: $[M+H]^+$ Calcd. for $C_{15}H_{11}D_2$ 195.1143; Found 195.1149.

IR (neat) (cm^{-1}): 3038, 2920, 1437, 1339, 1217, 1097, 923, 751.

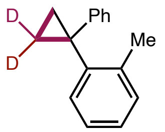

1-methyl-2-(1-phenylcyclopropyl-2,2-*d*$_2$)benzene (7)

Prepared according to **GP3** using 1-methyl-2-(1-phenylvinyl)benzene (0.2 mmol, 38.8 mg) and CD_2Cl_2 (2 mmol, 128 μL). The reaction mixture was purified by column chromatography (100% hexane) to provide the title compound **7** as a colorless oil (37 mg, 88%).

R*$_f$* = 0.58 (100% Hexane)

^{1}H NMR (400 MHz, CDCl$_3$): δ 7.36 – 7.30 (m, 1H), 7.14 – 7.07 (m, 5H), 7.04 – 6.98 (m, 1H), 6.87 – 6.83 (m, 2H), 2.14 (s, 3H), 1.27 (d, *J* = 4.6 Hz, 1H), 1.20 (d, *J* = 4.6 Hz, 1H).

^{13}C NMR (101 MHz, CDCl$_3$): δ 145.3, 142.4, 139.1, 130.9, 130.4, 128.1, 126.9, 125.8, 125.4, 125.1, 27.90, 19.8, 17.7.

HRMS (ESI) m/z: $[M+H]^+$ Calcd. for $C_{16}H_{15}D_2$ 211.1456; Found 211.1449.

IR (neat) (cm^{-1}): 3020, 2923, 1600, 1494, 1451, 1022, 750, 728, 694.

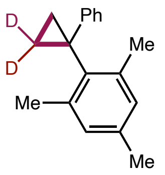

1,3,5-trimethyl-2-(1-phenylcyclopropyl-2,2-*d*$_2$)benzene (8)

Prepared according to **GP3** using 1,3,5-trimethyl-2-(1-phenylvinyl)benzene (0.2 mmol, 44.4 mg) and CD_2Cl_2 (2 mmol, 128 μL). The reaction mixture was purified by column chromatography (100% hexane) to provide the title compound **8** as a colorless oil (38 mg, 80%).

R*$_f$* = 0.60 (100% Hexane)

^{1}H NMR (400 MHz, CDCl$_3$): δ 7.13 – 7.08 (m, 2H), 7.04 – 6.98 (m, 1H), 6.81 (s, 2H), 6.76 – 6.70 (m, 2H), 2.22 (s, 3H), 2.15 (s, 6H), 1.37 (d, *J* = 4.7 Hz, 1H), 1.17 (d, *J* = 4.7 Hz, 1H).

^{13}C NMR (101 MHz, CDCl$_3$): δ 145.2, 139.2, 137.3, 136.2, 129.1, 128.2, 124.8, 124.3, 24.2, 21.9, 20.9, 20.6.

HRMS (ESI) m/z: $[M+H]^+$ Calcd. for $C_{18}H_{19}D_2$ 239.1769; Found 239.1764.

IR (neat) (cm^{-1}): 3002, 2918, 1600, 1493, 1452, 1027, 851, 751, 711, 693.

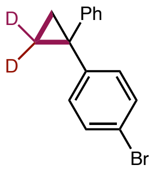

1-bromo-4-(1-phenylcyclopropyl-2,2-*d*$_2$)benzene (9)

Prepared according to **GP3** using 1-bromo-4-(1-phenylvinyl)benzene (0.2 mmol, 51.8 mg) and CD_2Cl_2 (2 mmol, 128 μL). The reaction mixture was purified by column chromatography (100% hexane) to provide the title compound **9** as a colorless oil (50 mg, 91%).

R*$_f$* = 0.58 (100% Hexane)

^{1}H NMR (400 MHz, CDCl$_3$): δ 7.32 – 7.27 (m, 2H), 7.20 – 7.16 (m, 2H), 7.14 – 7.07 (m, 3H), 7.03 – 6.98 (m, 2H), 1.21 (d, *J* = 4.7 Hz, 1H), 1.16 (d, *J* = 4.7 Hz, 1H).

^{13}C NMR (101 MHz, CDCl$_3$): δ 145.0, 144.8, 131.3, 130.1, 128.3, 128.3, 126.1, 119.7, 29.3, 16.2.

HRMS (ESI) m/z: $[M+H]^+$ Calcd. for $C_{15}H_{12}D_2Br$ 275.0404; Found 275.0403.

IR (neat) (cm^{-1}): 3024, 2925, 1493, 1073, 1007, 819, 756, 696.

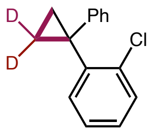

1-chloro-2-(1-phenylcyclopropyl-2,2-*d*$_2$)benzene (10)

Prepared according to **GP3** using 1-chloro-2-(1-phenylvinyl)benzene (0.2 mmol, 42.8 mg) and CD_2Cl_2 (2 mmol, 128 μL). The reaction mixture was purified by column chromatography (100% hexane) to provide the title compound **10** as a colorless oil (33 mg, 72%).

R*$_f$* = 0.52 (100% Hexane)

^{1}H NMR (400 MHz, $CDCl_3$): δ 7.43 (dd, J = 7.5, 1.9 Hz, 1H), 7.27 (dd, J = 7.5, 1.9 Hz, 1H), 7.19 – 7.10 (m, 4H), 7.06 – 7.01 (m, 1H), 6.99 – 6.94 (m, 2H), 1.31 (d, J = 5.0 Hz, 1H), 1.23 (d, J = 5.0 Hz, 1H).

^{13}C NMR (101 MHz, $CDCl_3$): δ 144.4, 142.0, 136.6, 132.6, 129.9, 128.1, 128.1, 126.8, 126.1, 125.5, 28.4, 17.4.

HRMS (ESI) m/z: $[M+H]^+$ Calcd. for $C_{15}H_{12}D_2Cl$ 231.0910; Found 231.0907.

IR (neat) (cm^{-1}): 3057, 2924, 2211, 1496, 1431, 1034, 751, 731, 693.

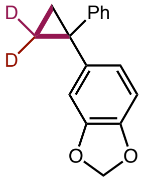

5-(1-phenylcyclopropyl-2,2-*d*$_2$)benzo[*d*][1,3]dioxole (11)

Prepared according to **GP3** using 5-(1-phenylvinyl)benzo[*d*][1,3]dioxole (0.2 mmol, 44.8 mg) and CD_2Cl_2 (2 mmol, 128 μL). The reaction mixture was purified by column chromatography (1% EtOAc/hexane) to provide the title compound **11** as a yellowish oil (37 mg, 77%).

R_f = 0.57 (5% EtOAc/Hexane)

^{1}H NMR (400 MHz, $CDCl_3$): δ 7.19 – 7.06 (m, 5H), 6.71 – 6.61 (m, 3H), 5.82 (s, 2H), 1.15 (d, J = 1.5 Hz, 2H).

^{13}C NMR (101 MHz, $CDCl_3$): δ 147.5, 146.0, 145.8, 139.6, 128.2, 127.8, 125.8, 121.8, 109.6, 107.9, 100.9, 29.6, 16.1.

HRMS (ESI) m/z: $[M+H]^+$ Calcd. for $C_{16}H_{13}D_2O_2$ 241.1198; Found 241.1198.

IR (neat) (cm^{-1}): 3022, 2956, 1483, 1433, 1225, 1036, 935, 809, 697.

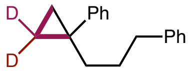

(3-(1-phenylcyclopropyl-2,2-*d*$_2$)propyl)benzene (12)

Prepared according to **GP3** using pent-4-ene-1,4-diyldibenzene (0.2 mmol, 44.4 mg) and CD_2Cl_2 (2 mmol, 128 μL). The reaction mixture was purified by column chromatography (100% hexane) to provide the title compound **12** as a colorless oil (42 mg, 88%).

R_f = 0.62 (100% Hexane)

^{1}H NMR (400 MHz, CDCl$_3$): δ 7.21 – 7.13 (m, 6H), 7.12 – 7.00 (m, 4H), 2.46 (qd, J = 6.5, 3.6 Hz, 2H), 1.57 – 1.50 (m, 4H), 0.69 (d, J = 4.3 Hz, 1H), 0.55 (d, J = 4.2 Hz, 1H).

^{13}C NMR (101 MHz, CDCl$_3$): δ 145.3, 142.6, 129.0, 128.3, 128.2, 128.0, 125.8, 125.6, 40.0, 36.0, 28.9, 25.4, 12.8.

HRMS (ESI) m/z: $[M+H]^+$ Calcd. for $C_{18}H_{19}D_2$ 239.1769; Found 239.1792.

IR (neat) (cm^{-1}): 3024, 2931, 1601, 1495, 142, 1029, 907, 731, 696.

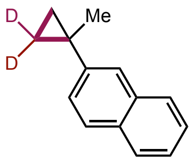

2-(1-methylcyclopropyl-2,2-d_2)naphthalene (13)

Prepared according to **GP3** using 2-(prop-1-en-2-yl)naphthalene (0.2 mmol, 33.6 mg) and CD_2Cl_2 (2 mmol, 128 μL). The reaction mixture was purified by column chromatography (100% hexane) to provide the title compound **13** as a colorless oil (33 mg, 90%).

R$_f$ = 0.64 (100% Hexane)

^{1}H NMR (400 MHz, CDCl$_3$): δ 7.87 – 7.74 (m, 4H), 7.54 – 7.39 (m, 3H), 1.56 (s, 3H), 1.02 (d, J = 4.4 Hz, 1H), 0.85 (d, J = 4.4 Hz, 1H).

^{13}C NMR (101 MHz, CDCl$_3$): δ 144.5, 133.5, 131.8, 127.8, 127.5, 127.5, 125.9, 125.6, 125.1, 124.9, 25.7, 19.8, 15.3.

HRMS (ESI) m/z: $[M+H]^+$ Calcd. for $C_{14}H_{13}D_2$ 185.1299; Found 185.1295.

IR (neat) (cm^{-1}): 3055, 2954, 1599, 1506, 1133, 854, 806, 744.

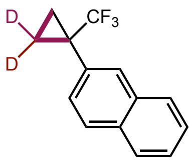

2-(1-(trifluoromethyl)cyclopropyl-2,2-d_2)naphthalene (14)

Prepared according to **GP3** using 2-(3,3,3-trifluoroprop-1-en-2-yl)naphthalene (0.2 mmol, 44.4 mg) and CD_2Cl_2 (2 mmol, 128 μL). The reaction mixture was purified by column chromatography (100% hexane) to provide the title compound **14** as a white solid. (46 mg, 97%).

R$_f$ = 0.45 (100% Hexane)

^{1}H NMR (400 MHz, CDCl$_3$): δ 7.94 (d, J = 1.8 Hz, 1H), 7.86 – 7.80 (m, 3H), 7.61 – 7.56 (m, 1H), 7.53 – 7.48 (m, 2H), 1.43 (d, J = 5.1 Hz, 1H), 1.13 (dd, J = 5.2, 2.1 Hz, 1H).

^{13}C NMR (101 MHz, CDCl$_3$): δ 133.5, 133.1, 133.0, 130.6, 128.6, 128.0, 127.9, 127.6, 126.4, 126.3, 28.3, 28.03, 9.7, 9.6.

^{19}F NMR (376 MHz, CDCl$_3$): δ -69.81.

HRMS (ESI) m/z: $[M+H]^+$ Calcd. for $C_{14}H_{10}D_2F_3$ 239.1017; Found 239.1012.

IR (neat) (cm^{-1}): 3057, 2924, 1598, 1505, 1343, 1124, 1052, 818, 750 .

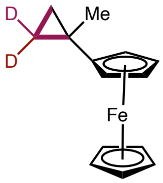

2-(1-methylcyclopropyl-2,2-*d*$_2$)ferrocene (15)

Prepared according to **GP3** using 2-(prop-1-en-2-yl)ferrocene (0.2 mmol, 45.2 mg) and CD_2Cl_2 (2 mmol, 128 μL). The reaction mixture was purified by column chromatography (100% hexane) to provide the title compound **14** as an orange solid (44 mg, 91%).

R$_f$ = 0.64 (Hexane)

^{1}H NMR (400 MHz, CDCl$_3$): δ 4.07 (s, 5H), 3.95 (t, J = 1.9 Hz, 2H), 3.91 (t, J = 1.9 Hz, 2H), 1.29 (s, 3H), 0.61 (d, J = 4.2 Hz, 1H), 0.58 (d, J = 4.2 Hz, 1H).

^{13}C NMR (101 MHz, CDCl$_3$): δ 68.3, 66.8, 65.2, 23.6, 17.7, 14.0.

HRMS (ESI) m/z: $[M+H]^+$ Calcd. for $C_{14}H_{15}D_2Fe$ 243.0805; Found 243.0794.

IR (neat) (cm^{-1}): 3093, 2955, 1485, 1441, 1376, 1142, 1104, 998, 812.

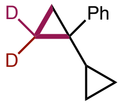

1-phenyl-1,1'-bi(cyclopropane)-2,2-*d*$_2$ (16)

Prepared according to **GP3** using (1-cyclopropylvinyl)benzene (0.2 mmol, 28.8 mg) and CD_2Cl_2 (2 mmol, 128 μL). The reaction mixture was purified by column chromatography (100% hexane) to provide the title compound **15** as a colorless oil (21 mg, 67%).

R*f* = 0.45 (Hexane)

^{1}H NMR (400 MHz, $CDCl_3$): δ 7.25 – 7.10 (m, 4H), 7.07 – 7.00 (m, 1H), 1.19 – 1.14 (m, 1H), 0.58 (d, *J* = 4.5 Hz, 1H), 0.49 (d, *J* = 4.5 Hz, 1H), 0.33 – 0.26 (m, 2H), 0.02 – -0.03 (m, 2H).

^{13}C NMR (101 MHz, $CDCl_3$): δ 146.7, 128.1, 127.7, 125.6, 29.7, 24.9, 17.1, 11.4, 2.7.

HRMS (ESI) m/z: $[M+Na]^+$ Calcd. for $C_{12}H_{12}D_2Na$ 183.1119; Found 183.1126.

IR (neat) (cm^{-1}): 3077, 2924, 1600, 1494, 1444, 1015, 795, 752, 698.

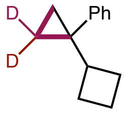

(1-cyclobutylcyclopropyl-2,2-*d*$_2$)benzene (17)

Prepared according to **GP3** using (1-cyclobutylvinyl)benzene (0.2 mmol, 34.8 mg) and CD_2Cl_2 (2 mmol, 128 μL). The reaction mixture was purified by column chromatography (100% hexane) to provide the title compound **16** as a colorless oil (32 mg, 84%).

R*f* = 0.82 (100% Hexane)

^{1}H NMR (400 MHz, $CDCl_3$): δ 7.34 – 7.28 (m, 4H), 7.26 – 7.18 (m, 1H), 2.77 – 2.66 (m, 1H), 1.91 – 1.83 (m, 2H), 1.81 – 1.71 (m, 1H), 1.70 – 1.60 (m, 3H), 0.79 (d, *J* = 4.1 Hz, 1H), 0.72 (d, *J* = 4.1 Hz, 1H).

^{13}C NMR (101 MHz, $CDCl_3$): δ 145.5, 129.2, 127.9, 125.8, 42.0, 27.9, 25.4, 17.3, 9.4.

HRMS (ESI) m/z: $[M+Na]^+$ Calcd. for $C_{13}H_{14}D_2Na$ 197.1275; Found 197.1283.

IR (neat) (cm^{-1}): 3062, 2923, 1492, 1442, 792, 732, 699.

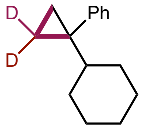

(1-cyclohexylcyclopropyl-2,2-*d*$_2$)benzene (18)

Prepared according to **GP3** using (1-cyclohexylvinyl)benzene (0.2 mmol, 37.2 mg) and CD_2Cl_2 (2 mmol, 128 μL). The reaction mixture was purified by column chromatography (100% hexane) to provide the title compound **17** as a colorless oil (35 mg, 87%).

R_f = 0.85 (100% Hexane)

^{1}H NMR (400 MHz, $CDCl_3$): δ 7.21 – 7.08 (m, 5H), 1.71 (ddq, *J* = 12.3, 3.9, 2.0 Hz, 2H), 1.64 – 1.57 (m, 2H), 1.50 – 1.43 (m, 1H), 1.12 – 1.01 (m, 2H), 0.90 – 0.78 (m, 3H), 0.68 (tt, *J* = 11.9, 3.0 Hz, 1H), 0.60 (d, *J* = 4.2 Hz, 1H), 0.54 (d, *J* = 4.2 Hz, 1H).

^{13}C NMR (101 MHz, $CDCl_3$): δ 143.8, 131.4, 127.4, 126.0, 47.6, 30.8, 30.7, 26.8, 26.3, 11.4.

HRMS (ESI) m/z: $[M+H]^+$ Calcd. for $C_{15}H_{19}D_2$ 203.1769; Found 203.1789.

IR (neat) (cm^{-1}): 3023, 2921, 1493, 1444, 1033, 793, 731, 699.

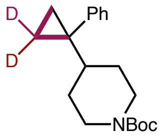

***tert*-butyl-4-(1-phenylcyclopropyl-2,2-*d*$_2$)piperidine-1-carboxylate (19)**

Prepared according to **GP3** using *tert*-butyl 4-(1-phenylvinyl)piperidine-1-carboxylate (0.2 mmol, 57.4 mg) and CD_2Cl_2 (2 mmol, 128 μL). The reaction mixture was purified by column chromatography (5% EtOAc/hexane) to provide the title compound **18** as a white solid (51 mg, 84%).

R_f = 0.39 (5% EtOAc/Hexane)

^{1}H NMR (400 MHz, $CDCl_3$): δ 7.29 – 7.22 (m, 5H), 4.11 (s, 2H), 2.63 – 2.52 (m, 2H), 1.71 (d, 12 Hz, 2H), 1.42 (s, 9H), 1.19 – 1.09 (m, 2H), 1.00 (dt, *J* = 12.1, 3.2 Hz, 1H), 0.76 (d, *J* = 4.3 Hz, 1H), 0.69 (d, *J* = 4.3 Hz, 1H).

^{13}C NMR (101 MHz, $CDCl_3$): δ 154.7, 143.0, 131.3, 127.6, 126.4, 79.1, 45.6, 30.4, 29.6, 28.4, 11.1.

HRMS (ESI) m/z: $[M+H]^+$ Calcd. for $C_{19}H_{26}D_2NO_2$ 304.2246; Found 304.2246.

IR (neat) (cm^{-1}): 3064, 2931, 1676, 1429, 1292, 1228, 1177, 1087, 699.

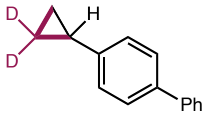

4-(cyclopropyl-2,2-*d*$_2$)-1,1'-biphenyl (20)

Prepared according to **GP3** using 4-vinyl-1,1'-biphenyl (0.2 mmol, 36 mg) and CD_2Cl_2 (2 mmol, 128 μL). The reaction mixture was purified by column chromatography (100% hexane) to provide the title compound **19** as an off-white solid (39 mg, 99%).

R_f = 0.55 (100% Hexane)

^{1}H NMR (400 MHz, CDCl$_3$): δ 7.62 – 7.56 (m, 2H), 7.54 – 7.49 (m, 2H), 7.47 – 7.41 (m, 2H), 7.37 – 7.31 (m, 1H), 7.20 – 7.13 (m, 2H), 1.94 (dd, J = 8.5, 5.2 Hz, 1H), 1.00 (dd, J = 8.5, 5.2 Hz, 1H), 0.77 – 0.72 (m, 1H).

^{13}C NMR (101 MHz, CDCl$_3$): δ 143.2, 141.1, 138.3, 128.7, 127.0, 126.9, 126.0, 14.9, 9.1, 8.7.

HRMS (ESI) m/z: $[M+H]^+$ Calcd. for $C_{15}H_{13}D_2$ 197.1299; Found 197.1295.

IR (neat) (cm^{-1}): 3031, 2922, 1596, 1486, 1407, 1037, 905, 820, 714.

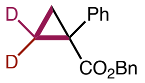

benzyl-1-phenylcyclopropane-1-carboxylate-2,2-*d*$_2$ (21)

Prepared according to **GP3** using benzyl 2-phenylacrylate (0.2 mmol, 47.6 mg) and CD_2Cl_2 (2 mmol, 128 μL). The reaction mixture was purified by column chromatography (2% EtOAc/hexane) to provide the title compound **20** as a yellowish oil (44 mg, 88%).

R$_f$ = 0.49 (5% EtOAc/Hexane)

^{1}H NMR (400 MHz, CDCl$_3$): δ 7.30 – 7.26 (m, 2H), 7.26 – 7.16 (m, 6H), 7.13 – 7.08 (m, 2H), 5.01 (s, 2H), 1.54 (d, J = 3.9 Hz, 1H), 1.13 (d, J = 3.9 Hz, 1H).

^{13}C NMR (101 MHz, CDCl$_3$): δ 174.3, 139.4, 136.2, 130.5, 128.4, 128.1, 127.8, 127.3, 127.2, 66.4, 29.0, 16.4.

HRMS (ESI) m/z: $[M+H]^+$ Calcd. for $C_{17}H_{15}D_2O_2$ 255.1354; Found 255.1349.

IR (neat) (cm^{-1}): 3030, 2920, 1717, 1497, 1289, 1173, 1069, 729, 694.

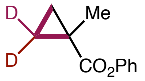

phenyl-1-methylcyclopropane-1-carboxylate-2,2-*d*$_2$ (22)

Prepared according to **GP3** using phenyl methacrylate (0.2 mmol, 32.4 mg) and CD_2Cl_2 (2 mmol, 128 μL). The reaction mixture was purified by column chromatography (2% EtOAc/hexane) to provide the title compound **22** as a yellowish oil (27 mg, 76%).

R$_f$ = 0.45 (5% EtOAc/Hexane)

^{1}H NMR (400 MHz, CDCl$_3$): δ 7.41 – 7.31 (m, 2H), 7.24 – 7.16 (m, 1H), 7.09 – 7.01 (m, 2H), 1.42 (s, 4H), 0.82 (d, J = 3.9 Hz, 1H).

^{13}C NMR (101 MHz, CDCl$_3$): δ 174.6, 151.0, 129.3, 125.5, 121.5, 19.3, 18.6, 17.2.

HRMS (ESI) m/z: $[M+H]^+$ Calcd. for $C_{11}H_{11}D_2O_2$ 179.1041; Found 179.1036.

IR (neat) (cm^{-1}): 3010, 2969, 1737, 1492, 1320, 1195, 1118, 922, 737, 688.

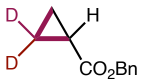

benzylcyclopropane-1-carboxylate-2,2-*d*$_2$ (23)

Prepared according to **GP3** using benzyl acrylate (0.2 mmol, 32.4 mg) and CD_2Cl_2 (2 mmol, 128 µL). The reaction mixture was purified by column chromatography (2% EtOAc/hexane) to provide the title compound **23** as a yellowish oil (coeluted with unreacted alkene as 5:1 mixture, 27 mg, 63% title compound **23**).

R$_f$ = 0.45 (5% EtOAc/Hexane)

^{1}H NMR (400 MHz, CDCl$_3$): δ 7.36 (s, 5H), 5.13 (s, 2H), 1.65 (dd, J = 8.1, 4.6 Hz, 1H), 1.06 – 1.00 (m, 1H), 0.89 – 0.84 (m, 1H).

^{13}C NMR (101 MHz, CDCl$_3$): δ 174.7, 136.1, 131.0, 128.5, 128.1, 66.3, 66.2, 12.7, 8.3.

HRMS (ESI) m/z: $[M+H]^+$ Calcd. for $C_{11}H_{11}D_2O_2$ 179.1041; Found 179.1042.

IR (neat) (cm^{-1}): 3059, 2977, 1699, 1441, 1340, 1189, 1042, 731.

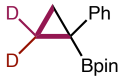

4,4,5,5-tetramethyl-2-(1-phenylcyclopropyl-2,2-*d*$_2$)-1,3,2-dioxaborolane (24)

Prepared according to **GP3** using 4,4,5,5-tetramethyl-2-(1-phenylvinyl)-1,3,2-dioxaborolane (0.2 mmol, 46 mg) and CD_2Cl_2 (2 mmol, 128 µL). The reaction mixture was purified by column chromatography (2% EtOAc/hexane) to provide the title compound **19** as a yellowish oil (45 mg, 91%).

R$_f$ = 0.53 (5% EtOAc/Hexane)

^{1}H NMR (400 MHz, CDCl$_3$): δ 7.21 – 7.12 (m, 4H), 7.08 – 7.01 (m, 1H), 1.14 (s, 12H), 1.02 (d, J = 3.5 Hz, 1H), 0.82 (d, J = 3.5 Hz, 1H).

^{13}C NMR (101 MHz, CDCl$_3$): δ 144.8, 128.9, 127.9, 125.2, 83.3, 24.6, 13.2.

HRMS (ESI) m/z: $[M+H]^+$ Calcd. for $C_{15}H_{20}D_2BO_2$ 247.1838; Found 247.1834.

IR (neat) (cm^{-1}): 3079, 2975, 1418, 1372, 1326, 1191, 1144, 852, 752, 699.

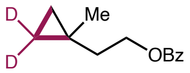

2-(1-methylcyclopropyl-2,2-d_2)ethyl benzoate (25)

Prepared according to **GP3** using 3-methylbut-3-en-1-yl benzoate (0.2 mmol, 38 mg) and CD_2Cl_2 (2 mmol, 128 μL). The reaction mixture was purified by column chromatography (2% EtOAc/hexane) to provide the title compound **22** as a colorless liquid (36 mg, 86%).

R_f = 0.48 (5% EtOAc/Hexane)

^{1}H NMR (400 MHz, $CDCl_3$): δ 8.06 – 8.03 (m, 2H), 7.57 – 7.53 (m, 1H), 7.46 – 7.42 (m, 2H), 4.43 (t, J = 6.9 Hz, 2H), 1.71 (t, J = 6.9 Hz, 2H), 1.12 (s, 3H), 0.35 (d, J = 4.4 Hz, 1H), 0.29 (d, J = 4.4 Hz, 1H).

^{13}C NMR (101 MHz, $CDCl_3$): δ 166.6, 132.7, 130.5, 129.5, 128.3, 77.2, 63.7, 63.1, 37.9, 22.7, 12.8, 12.5.

HRMS (ESI) m/z: $[M+H]^+$ Calcd. for $C_{13}H_{15}D_2O_2$ 207.1354; Found 207.1353.

IR (neat) (cm^{-1}): 3068, 2956, 1718, 1451, 1281, 1268, 1117, 1026, 710.

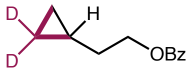

2-(cyclopropyl-2,2-d_2)ethyl benzoate (26)

Prepared according to **GP3** using but-3-en-1-yl benzoate (0.6 mmol, 105.6 mg) and CD_2Cl_2 (6 mmol, 384 μL). The reaction mixture was purified by column chromatography (2% EtOAc/hexane) to provide the title compound **23** as a colorless liquid (15 mg, 13%).

R_f = 0.48 (5% EtOAc/Hexane)

^{1}H NMR (400 MHz, $CDCl_3$): δ 8.01 – 7.96 (m, 2H), 7.51 – 7.46 (m, 1H), 7.40 – 7.34 (m, 2H), 4.33 (t, J = 6.6 Hz, 2H), 1.59 (t, J = 6.6 Hz, 2H), 0.73 (m, 1H), 0.41 (dd, J = 8.3, 4.5 Hz, 1H), 0.04 (dd, J = 8.3, 4.9 Hz, 1H).

^{13}C NMR (101 MHz, $CDCl_3$): δ 166.7, 132.7, 130.5, 129.5, 128.3, 65.1, 33.7, 7.6, 3.9.

HRMS (ESI) m/z: $[M+H]^+$ Calcd. for $C_{12}H_{13}D_2O_2$ 193.1198; Found 193.1193.

IR (neat) (cm^{-1}): 3068, 2942, 1716, 1451, 1275, 1115, 1026, 918, 715.

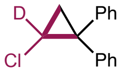

(2-chlorocyclopropane-1,1-diyl-2-*d*)dibenzene (27)

Prepared according to **GP3** using diphenylethylene (0.2 mmol, 36 mg) and $CDCl_3$ (2 mmol, 160 µL). The reaction mixture was purified by column chromatography (100% hexane) to provide the title compound **24** as a colorless oil (46 mg, 99%).

$\mathbf{R_f}$ = 0.47 (100% Hexane)

^{1}H NMR (400 MHz, $CDCl_3$): δ 7.46 – 7.41 (m, 2H), 7.40 – 7.34 (m, 2H), 7.32 – 7.24 (m, 5H), 7.24 – 7.18 (m, 1H), 1.82 – 1.75 (m, 2H).

^{13}C NMR (101 MHz, $CDCl_3$): δ 144.2, 139.6, 130.5, 128.6, 128.3, 127.7, 127.1, 126.6, 39.1, 36.8, 23.5.

HRMS (ESI) m/z: $[M+H]^+$ Calcd. for $C_{15}H_{13}DCl$ 230.0847; Found 230.0866.

IR (neat) (cm^{-1}): 3025, 2923, 1599, 1492, 1444, 1148, 1013, 746, 692.

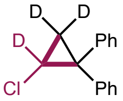

(2-chlorocyclopropane-1,1-diyl-2,3,3-d_3)dibenzene (28)

Prepared according to **GP3** using diphenylethylene-d_2 (0.2 mmol, 36 mg) and $CDCl_3$ (2 mmol, 160 µL). The reaction mixture was purified by column chromatography (100% hexane) to provide the title compound **25** as a colorless oil (45 mg, 98%).

$\mathbf{R_f}$ = 0.47 (100% Hexane)

^{1}H NMR (400 MHz, $CDCl_3$): δ 7.46 – 7.41 (m, 2H), 7.39 – 7.34 (m, 2H), 7.30 – 7.23 (m, 5H), 7.22 – 7.17 (m, 1H).

^{13}C NMR (101 MHz, $CDCl_3$): δ 144.2, 139.6, 130.6, 128.6, 128.2, 127.7, 127.1, 126.6, 39.2, 36.7.

HRMS (ESI) m/z: $[M+H]^+$ Calcd. for $C_{15}H_{11}D_3Cl$ 232.0972; Found 232.0969.

IR (neat) (cm^{-1}): 3025, 2923, 1600, 1491, 1444, 1164, 1078, 1037, 924, 825, 773, 690.

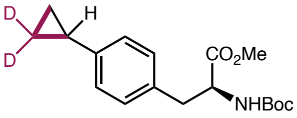

methyl (*S*)-2-((*tert*-butoxycarbonyl)amino)-3-(4-cyclopropyl-2,2-d_2)phenyl)propanoate (29)

Prepared according to **GP3** using methyl (*S*)-2-((*tert*-butoxycarbonyl)amino)-3-(4-vinylphenyl)propanoate (0.2 mmol, 61 mg) and CD_2Cl_2 (2 mmol, 128 μL). The reaction mixture was purified by column chromatography (15% EtOAc/Hexane) to provide the title compound **26** as a colorless oil (58 mg, 90%).

R$_f$ = 0.37 (20% EtOAc/Hexane)

^{1}H NMR (400 MHz, CDCl$_3$): δ 7.00 (s, 4H), 4.94 (d, *J* = 8.4 Hz, 1H), 4.56 (d, *J* = 7.6 Hz, 1H), 3.72 (s, 3H), 3.04 (qd, *J* = 13.8, 5.8 Hz, 2H), 1.85 (dd, *J* = 8.5, 5.1 Hz, 1H), 1.42 (s, 9H), 0.94 – 0.90 (m, 1H), 0.67 – 0.62 (m, 1H).

^{13}C NMR (101 MHz, CDCl$_3$): δ 172.4, 155.1, 142.7, 132.8, 129.2, 125.8, 79.8, 54.4, 52.1, 37.8, 29.7, 28.3, 14.8, 9.0, 8.9.

HRMS (ESI) m/z: $[M+H]^+$ Calcd. for $C_{18}H_{24}D_2NO_4$ 322.1987; Found 322.1982.

IR (neat) (cm^{-1}): 3369, 2927, 1714, 1497, 1365, 1161, 1017, 786.

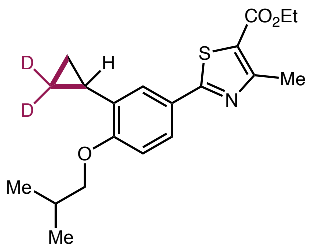

ethyl-2-(3-(cyclopropyl-2,2-d_2)-4-isobutoxyphenyl)-4-methylthiazole-5-carboxylate (30)

Prepared according to **GP3** using ethyl 2-(4-isobutoxy-3-vinylphenyl)-4-methylthiazole-5-carboxylate 0.2 mmol, 69 mg) and CD_2Cl_2 (2 mmol, 128 μL). The reaction mixture was purified by column chromatography (5% EtOAc/Hexane) to provide the title compound **27** as a colorless oil. (72 mg, 99%).

R$_f$ = 0.31 (10% EtOAc/Hexane)

^{1}H NMR (400 MHz, CDCl$_3$): δ 7.72 (dd, *J* = 8.5, 2.3 Hz, 1H), 7.46 (dd, *J* = 2.3, 0.6 Hz, 1H), 6.83 (d, *J* = 8.5 Hz, 1H), 4.34 (q, *J* = 7.1 Hz, 2H), 3.81 (d, *J* = 6.4 Hz, 2H), 2.75 (s, 3H), 2.22 – 2.10 (m, 2H), 1.38 (t, *J* = 7.1 Hz, 3H), 1.08 (d, *J* = 6.7 Hz, 6H), 0.94 (dd, *J* = 8.5, 4.5 Hz, 1H), 0.73 (t, *J* = 5.0 Hz, 1H).

^{13}C NMR (101 MHz, CDCl$_3$): δ 170.3, 162.4, 160.9, 160.4, 133.0, 125.4, 125.3, 123.9, 120.5, 110.9, 74.6, 61.0, 28.4, 19.3, 17.5, 14.3, 9.6, 7.3.

HRMS (ESI) m/z: $[M+H]^+$ Calcd. for $C_{20}H_{24}D_2NO_3S$ 362.1759; Found 362.1752.

IR (neat) (cm^{-1}): 2959, 2872, 1708, 1602, 1433, 1247, 1089, 907, 807, 729.

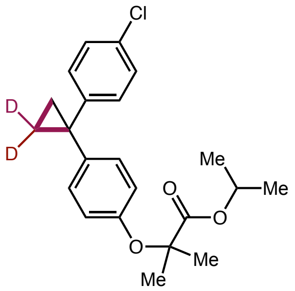

isopropyl-2-(4-(1-(4-chlorophenyl)cyclopropyl-2,2-d_2)phenoxy)-2-methylpropanoate (31)

Prepared according to **GP3** using isopropyl 2-(4-(1-(4-chlorophenyl)vinyl)phenoxy)-2-methylpropanoate (0.2 mmol, 71.6 mg) and CD_2Cl_2 (1 mmol, 64 µL). The reaction mixture was purified by column chromatography (2% EtOAc/ hexane) to provide the title compound **28** as a yellowish oil (64 mg, 86%).

R*f* = 0.46 (5% EtOAc/hexane)

^{1}H NMR (400 MHz, $CDCl_3$): δ 7.15 – 7.10 (m, 2H), 7.04 – 6.97 (m, 4H), 6.69 – 6.65 (m, 2H), 5.00 (p, *J* = 6.3 Hz, 1H), 1.49 (s, 6H), 1.15 (d, *J* = 4.5 Hz, 1H), 1.14 (d, *J* = 6.3 Hz, 6H), 1.12 (d, *J* = 4.5 Hz, 1H).

^{13}C NMR (101 MHz, $CDCl_3$): δ 173.7, 153.8, 144.6, 138.4, 131.5, 129.4, 129.1, 128.2, 118.8, 79.0, 68.8, 28.5, 25.4, 21.5, 16.2.

HRMS (ESI) m/z: $[M+H]^+$ Calcd. for $C_{22}H_{24}D_2ClO_3$ 375.1696; Found 375.1692.

IR (neat) (cm^{-1}): 3034, 2981, 1726, 1508, 1283, 1235, 1146, 1100, 1010, 819.

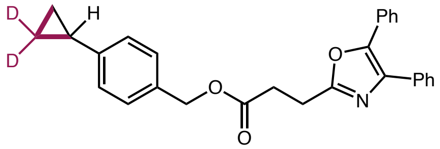

4-(cyclopropyl-2,2-d_2)benzyl 3-(4,5-diphenyloxazol-2-yl)propanoate (32)

Prepared according to **GP3** using 4-vinylbenzyl 3-(4,5-diphenyloxazol-2-yl)propanoate 0.2 mmol, 81.8 mg) and CD_2Cl_2 (2 mmol, 128 µL). The reaction mixture was purified by column chromatography (15% EtOAc/Hexane) to provide the title compound **29** as a colorless oil. (83 mg, 97%).

R*f* = 0.33 (20% EtOAc/Hexane)

^{1}H NMR (400 MHz, $CDCl_3$): δ 7.66 – 7.61 (m, 2H), 7.59 – 7.54 (m, 2H), 7.40 – 7.32 (m, 6H), 7.24 (d, *J* = 7.8 Hz, 2H), 7.01 (d, *J* = 7.9 Hz, 2H), 5.14 (s, 2H), 3.21 (t, *J* = 7.4 Hz, 2H), 2.97 (t, *J* = 7.4 Hz, 2H), 1.87 (dd, *J* = 8.5, 5.1 Hz, 1H), 0.95 (dd, *J* = 8.6, 4.5 Hz, 1H), 0.66 (t, *J* = 4.9 Hz, 1H).

^{13}C NMR (101 MHz, $CDCl_3$): δ 171.8, 161.7, 145.4, 144.3, 135.1, 132.7, 132.5, 129.0, 128.6, 128.5, 128.4, 128.3, 128.0, 127.9, 126.5, 125.8, 66.5, 31.2, 29.7, 23.5, 15.0, 9.1.

HRMS (ESI) m/z: $[M+H]^+$ Calcd. for $C_{28}H_{24}D_2NO_3$ 426.2038; Found 426.2034.

IR (neat) (cm^{-1}): 3074**,** 2956, 1733, 1604, 1444, 1159, 1045, 906, 814, 727.

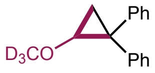

(2-(methoxy-*d*$_3$)cyclopropane-1,1-diyl)dibenzene (33)

Prepared according to **GP4** using 1,1-diphenylethylene (0.1 mmol, 18 mg) and CD_3OCHCl_2 (1 mmol, 118 mg). The reaction mixture was purified by column chromatography (3% EtOAc/Hexane) to provide the title compound **30** as a yellowish oil. (15 mg, 66%).

R_f = 0.31 (3% EtOAc/Hexane)

^{1}H NMR (400 MHz, $CDCl_3$): δ 7.37 – 7.27 (m, 8H), 7.25 – 7.19 (m, 2H), 3.78 (dd, J = 6.7, 3.8 Hz, 1H), 1.66 (dd, J = 6.1, 3.8 Hz, 1H), 1.44 (t, J = 6.4 Hz, 1H).

^{13}C NMR (101 MHz, $CDCl_3$): δ 144.8, 140.3, 129.6, 128.7, 128.5, 127.9, 126.2, 126.1, 65.7, 36.4, 21.0.

HRMS (ESI) m/z: $[M+H]^+$ Calcd. for $C_{16}H_{14}D_3O$ 228.1468; Found 228.1464.

IR (neat) (cm^{-1}): 3024, 2962, 1599, 1495, 1183, 1109, 1040, 749, 694.

V. References

1. Walker, J. C. L.; Oestreich, M. Regioselective Transfer Hydrodeuteration of Alkenes with a Hydrogen Deuteride Surrogate Using $B(C_6F_5)_3$ Catalysis. *Org. Lett.* **2018**, *20* (20), 6411–6414.

2. Zhang, S.; Shen, Z.; Jian, H. Cu/Ni-Catalyzed Cyanomethylation of Alkenes with Acetonitrile for the Synthesis of β,γ-Unsaturated Nitriles. *J. Org. Chem.* **2020**, *85* (9), 6143–6150.

3. Sakakibara, Y.; Itami, K.; Murakami, K. Switchable Decarboxylation by Energy- or Electron-Transfer Photocatalysis. *J. Am. Chem. Soc.* **2024**, *146* (2), 1554–1562.

4. Ranjan, P.; Pillitteri, S.; Coppola, G.; Oliva, M.; Van der Eycken, E. V.; Sharma, U. K. Unlocking the Accessibility of Alkyl Radicals from Boronic Acids through Solvent-Assisted Organophotoredox Activation. *ACS Catal.* **2021**, *11* (17), 10862–10870.

5. Pang, H.; Wang, Y.; Gallou, F.; Lipshutz, B. H. Fe-Catalyzed Reductive Couplings of Terminal (Hetero)Aryl Alkenes and Alkyl Halides under Aqueous Micellar Conditions. *J. Am. Chem. Soc.* **2019**, *141* (43), 17117–17124.

6. Le Saux, E.; Zanini, M.; Melchiorre, P. Photochemical Organocatalytic Benzylation of Allylic C-H Bonds. *J. Am. Chem. Soc.* **2022**, *144* (3), 1113–1118.

7. Yu, S.; Noble, A.; Bedford, R. B.; Aggarwal, V. K. Methylenespiro[2.3]Hexanes via Nickel-Catalyzed Cyclopropanations with [1.1.1]Propellane. *J. Am. Chem. Soc.* **2019**, *141* (51), 20325–20334.

8. Hu, C.; Chen, F.; Lu, G.-P.; Yi, W.-B. Deuterated N-Difluoromethylthiophthalimide: A Stable, Scalable Reagent for Radical and Electrophilic Deuteriodifluoromethylthiolations. *Chin. Chem. Lett.* **2022**, *33* (9), 4293–4297.

9. Yu, J.; Wu, Z.; Zhu, C. Efficient Docking-Migration Strategy for Selective Radical Difluoromethylation of Alkenes. *Angew. Chem. Int. Ed.* **2018**, *57* (52), 17156–17160.

10. Li, L.; Hilt, G. Regiodivergent DH or HD Addition to Alkenes: Deuterohydrogenation versus Hydrodeuterogenation. *Org. Lett.* **2020**, *22* (4), 1628–1632.

11. Hota, P. K.; Jose, A.; Mandal, S. K. Stereo- and Regioselective Addition of Arene to Alkyne Using Abnormal NHC Based Palladium Catalysts: Elucidating the Role of Trifluoroacetic Acid in Fujiwara Process. *Organometallics* **2017**, *36* (22), 4422–4431.

12. Dai, Z.-Y.; Nong, Z.-S.; Song, S.; Wang, P.-S. Asymmetric Photocatalytic C(Sp3)-H Bond Addition to α-Substituted Acrylates. *Org. Lett.* **2021**, *23* (8), 3157–3161.

13. Cong, F., Wei, Y.; Tang, P. Combining Photoredox and Silver Catalysis for Azidotrifluoromethoxylation of Styrenes. *Chem. Commun.* **2018**, *54* (35), 4473–4476.

14. Lan, Y.; Xie, S.; Liu, B. Regioselective 1,2-Di(Hetero)Arylation of Activated and Unactivated Alkenes with (Hetero)Aryl Chlorides. *Org. Lett.* **2025**, *27* (19), 4952–4957.

15. Zheng, J.; Bai, J.-F.; Jia, Y.; Qian, T.; Tang, J.; Jiang, Z.-J.; Chen, J.; Gao, Z. $B(C_6F_5)_3$-Catalyzed Regioselective Deuteration of Terminal Olefins Using D_2O as the Deuterium Source. *Org. Lett.* **2025**, *27* (21), 5324–5330.

16. Budai, B.; Leclair, A.; Wang, Q.; Zhu, J. Copper-Catalyzed 1,2-Methoxy Methoxycarbonylation of Alkenes with Methyl Formate. *Angew. Chem. Int. Ed.* **2019**, *58* (30), 10305–10309.

17. Kimura, Y.; Warashina, T. Convenient Preparation of Dichloromethyl Alkyl Ethers. *Tetrahedron Lett.* **2017**, *58* (49), 4598–4599.

VI. NMR Spectra

^{1}H NMR Spectrum of **1** (400 MHz, $CDCl_3$)

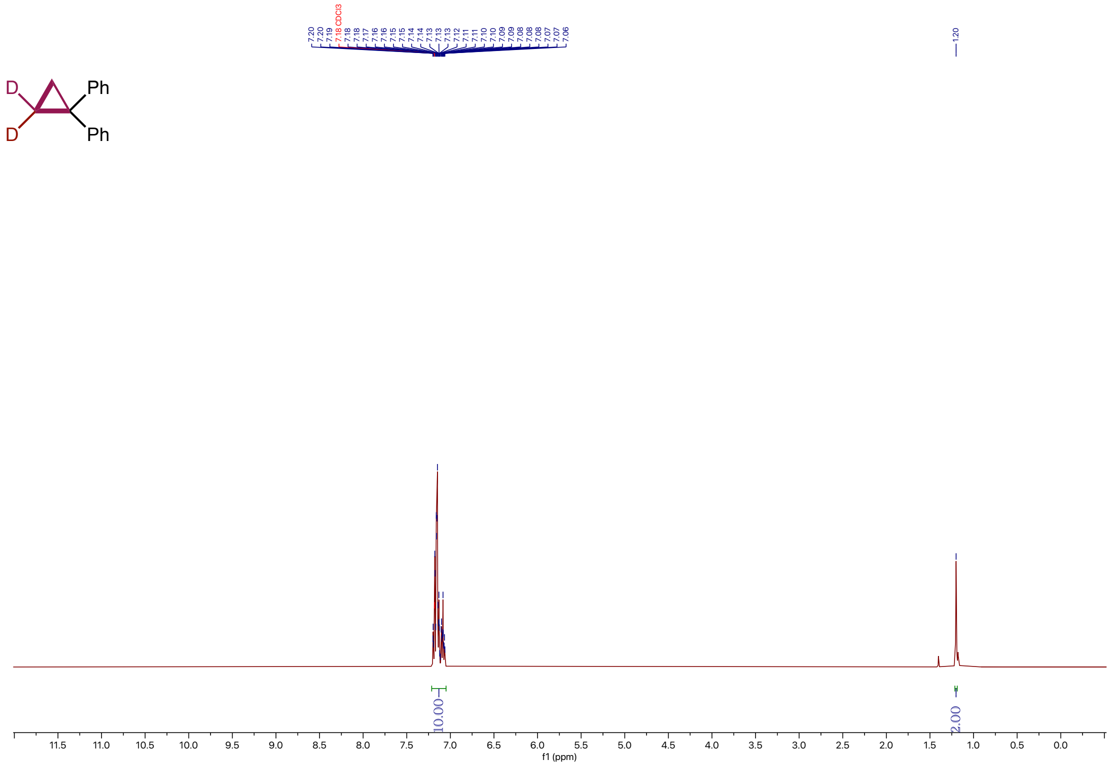

^{13}C NMR Spectrum of **1** (101 MHz, $CDCl_3$)

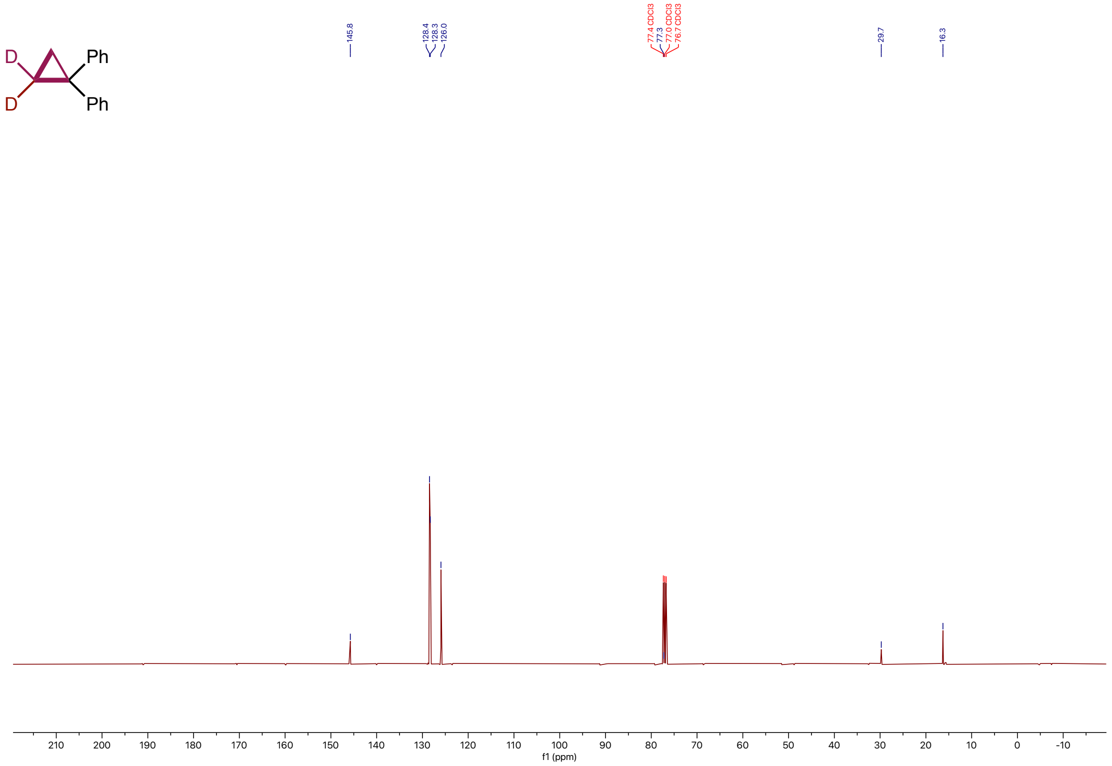

^1H NMR Spectrum of **2** (400 MHz, $CDCl_3$)

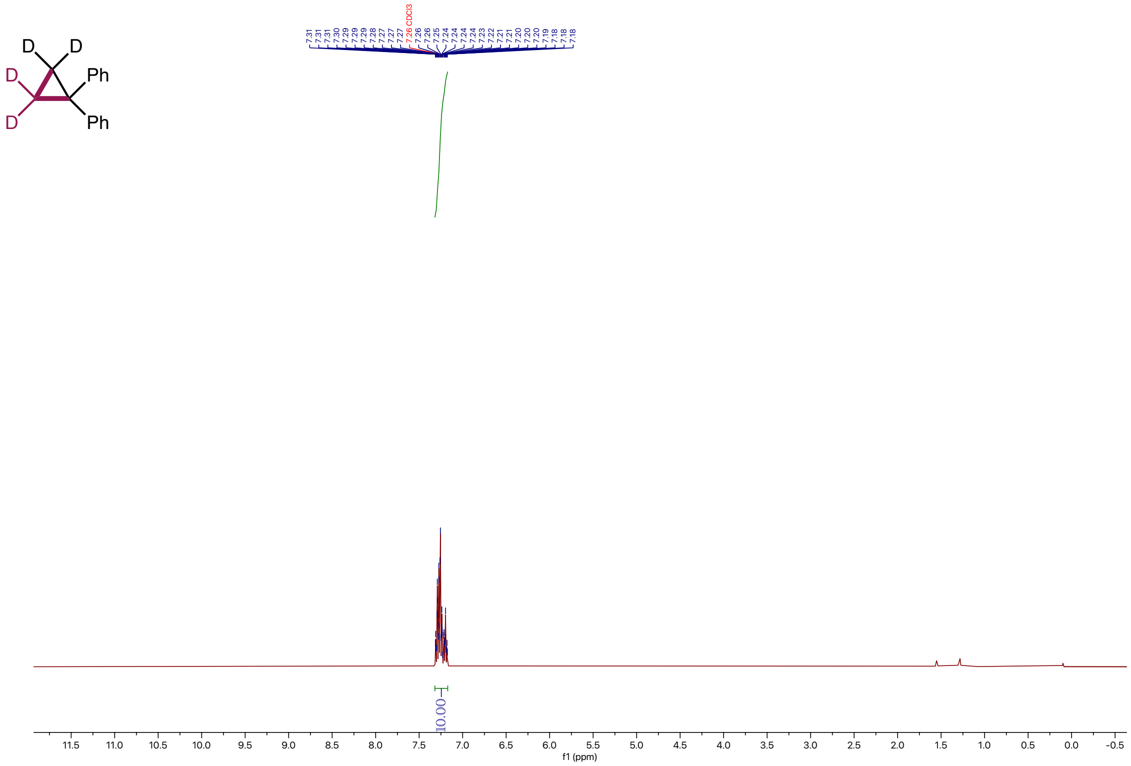

^{13}C NMR Spectrum of **2** (101 MHz, $CDCl_3$)

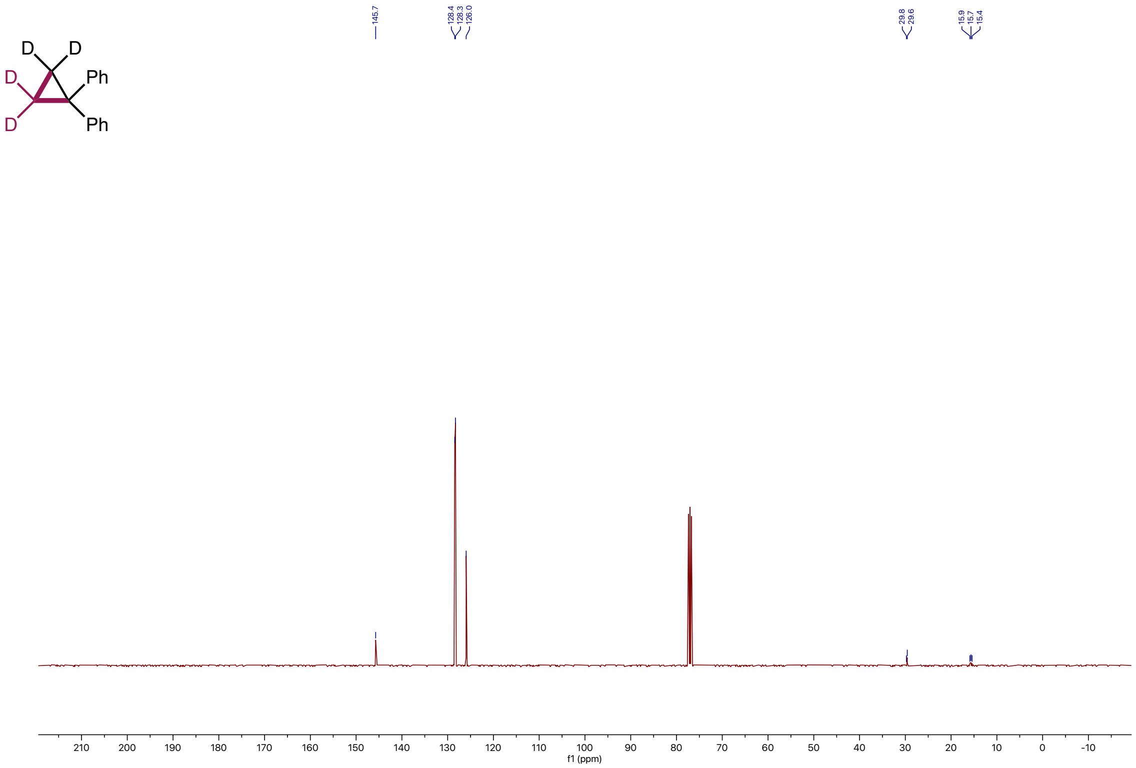

^{1}H NMR Spectrum of **3** (400 MHz, $CDCl_3$)

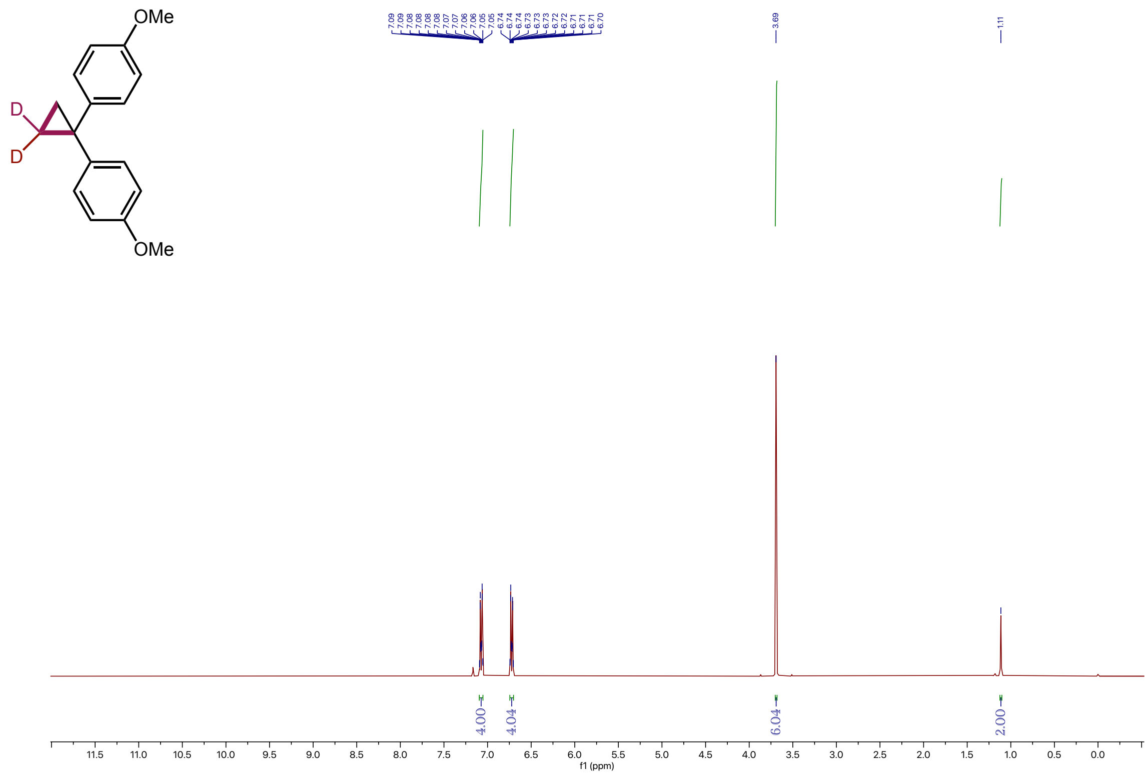

^{13}C NMR Spectrum of **3** (101 MHz, $CDCl_3$)

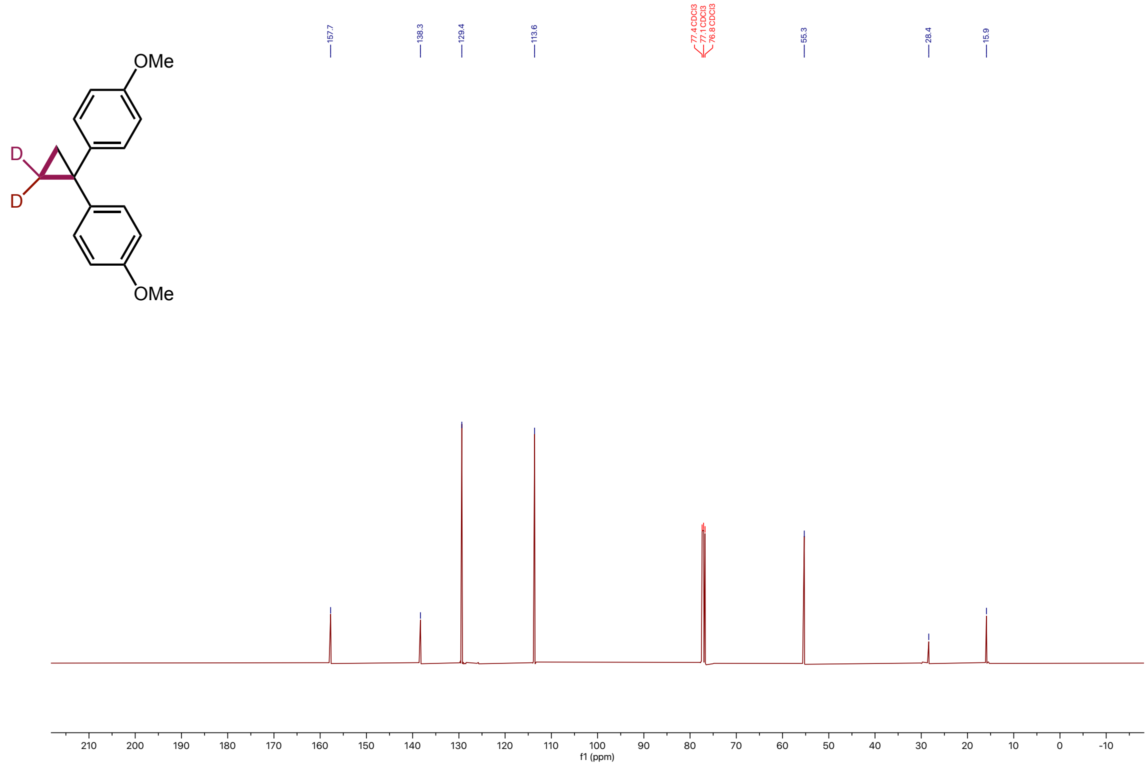

^{1}H NMR Spectrum of **4** (400 MHz, $CDCl_3$)

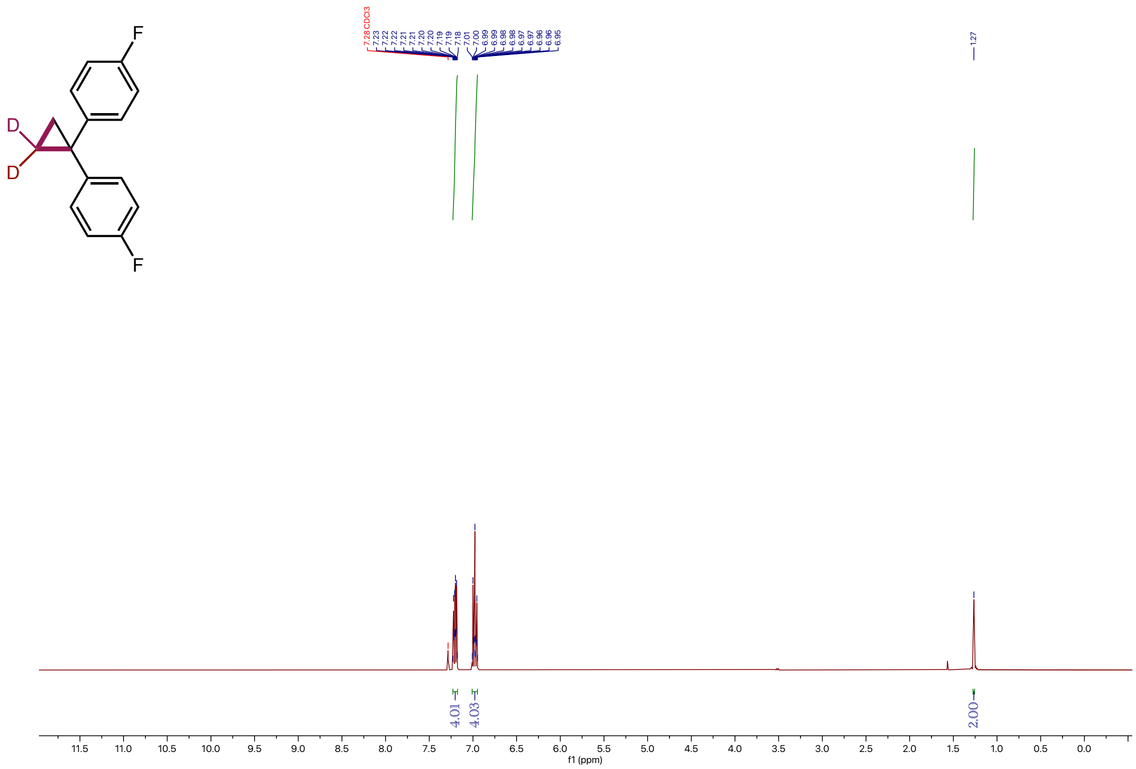

^{13}C NMR Spectrum of **4** (101 MHz, $CDCl_3$)

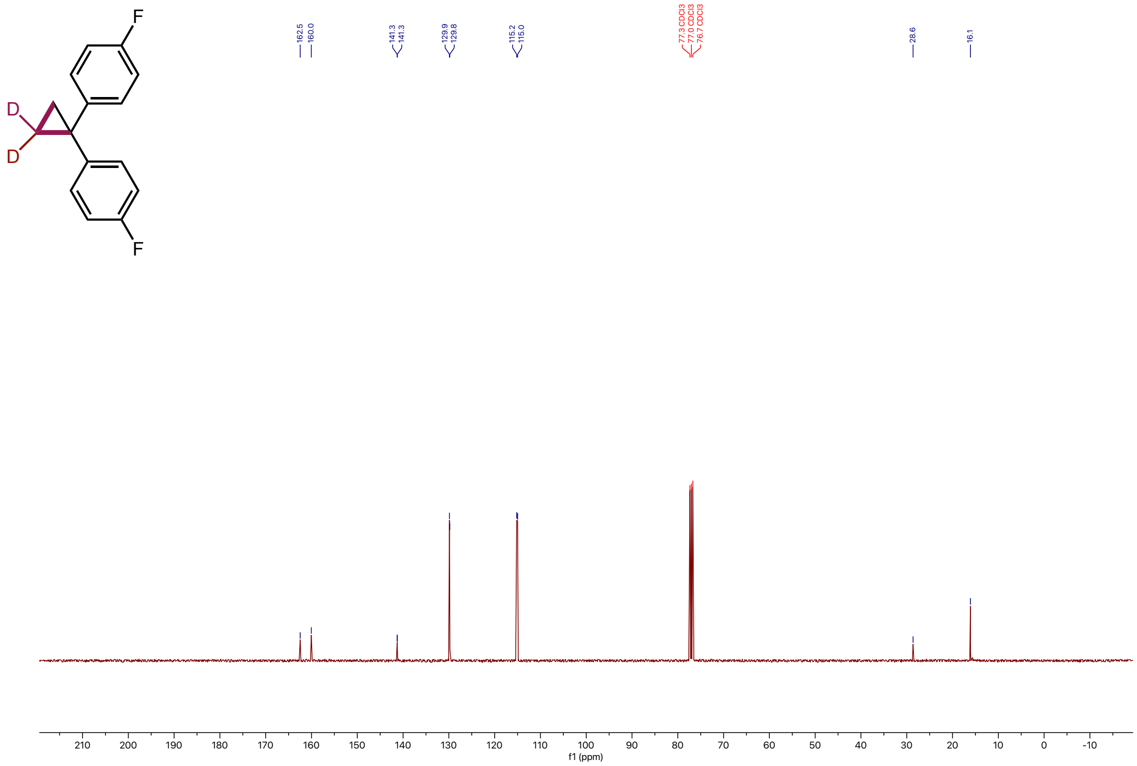

^{19}F NMR Spectrum of **4** (376 MHz, $CDCl_3$)

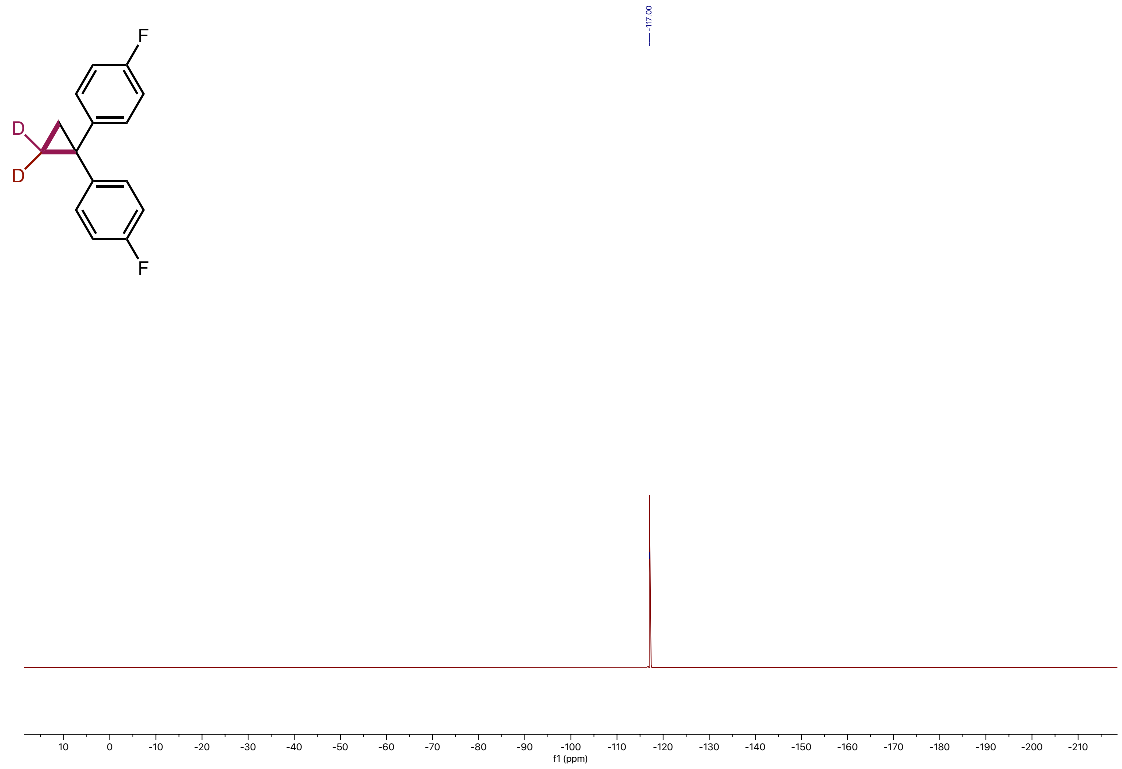

^{1}H NMR Spectrum of **5** (400 MHz, $CDCl_3$)

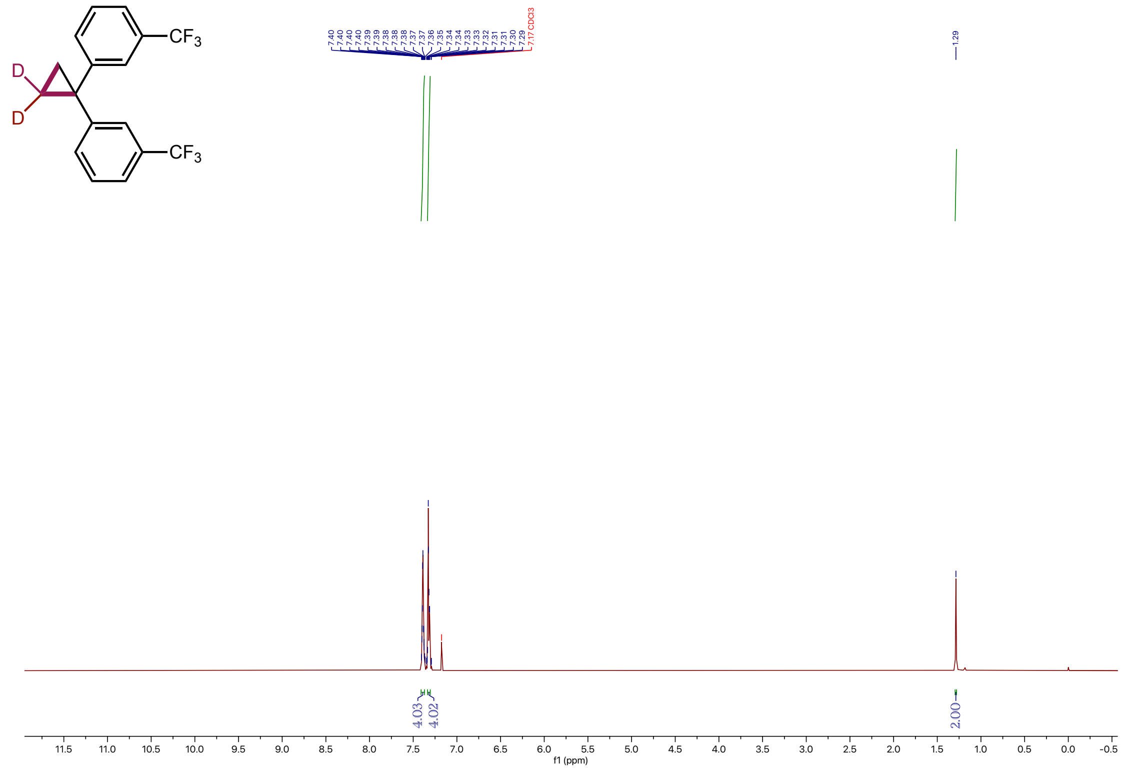

^{13}C NMR Spectrum of **5** (101 MHz, $CDCl_3$)

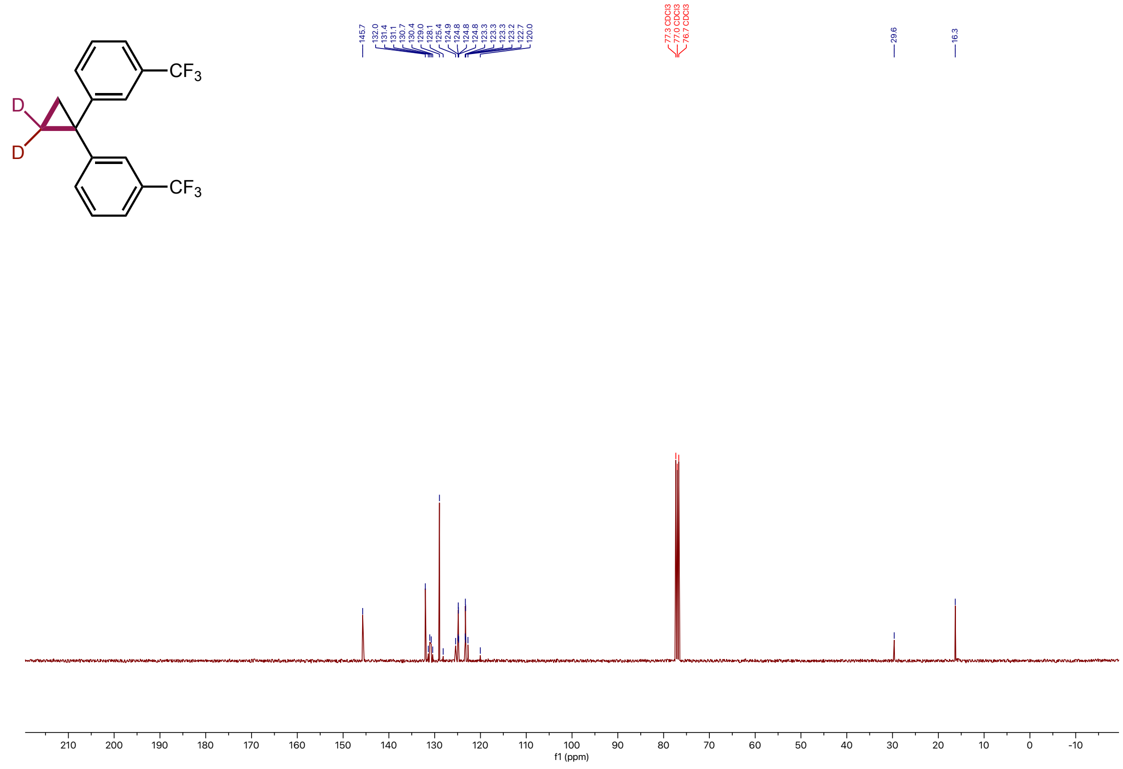

^{19}F NMR Spectrum of **5** (376 MHz, $CDCl_3$)

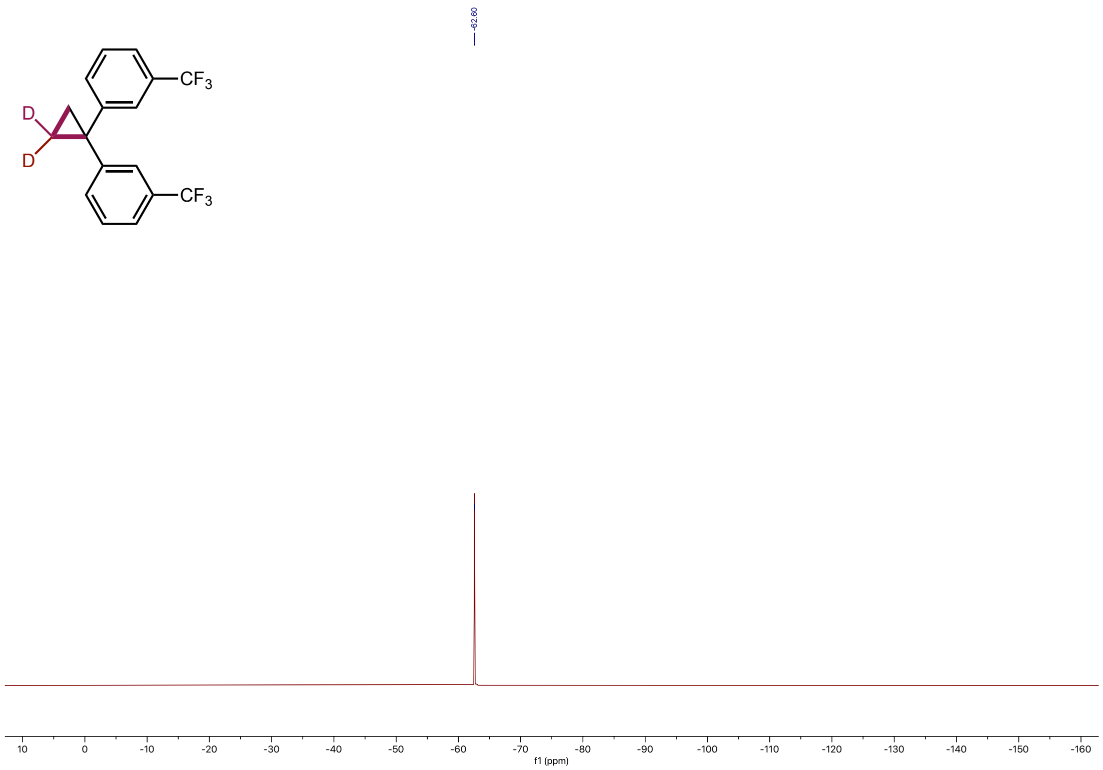

^{1}H NMR Spectrum of **6** (400 MHz, $CDCl_3$)

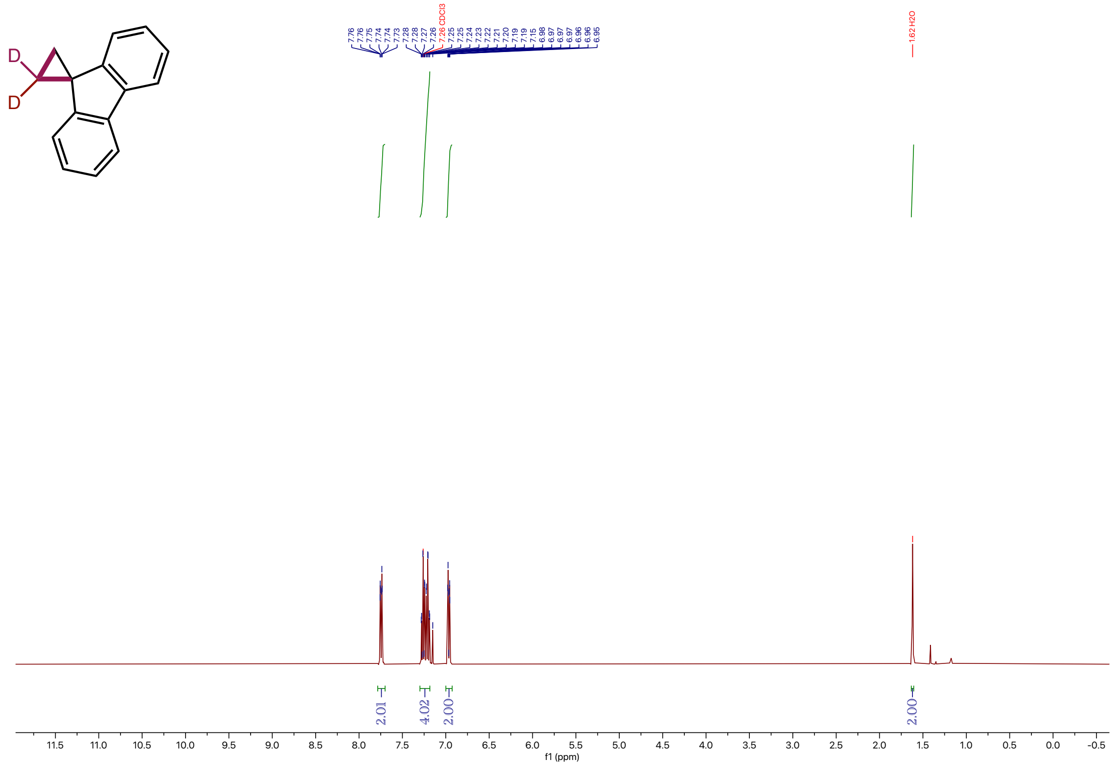

^{13}C NMR Spectrum of **6** (101 MHz, $CDCl_3$)

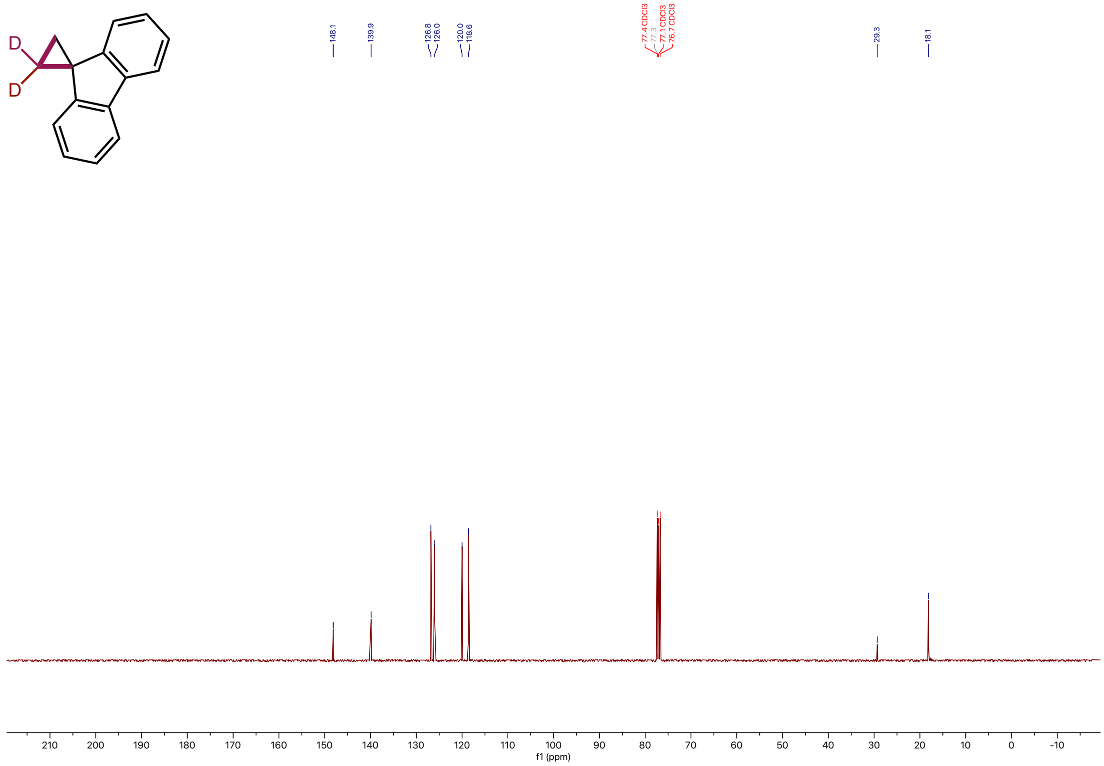

^{1}H NMR Spectrum of **7** (400 MHz, $CDCl_3$)

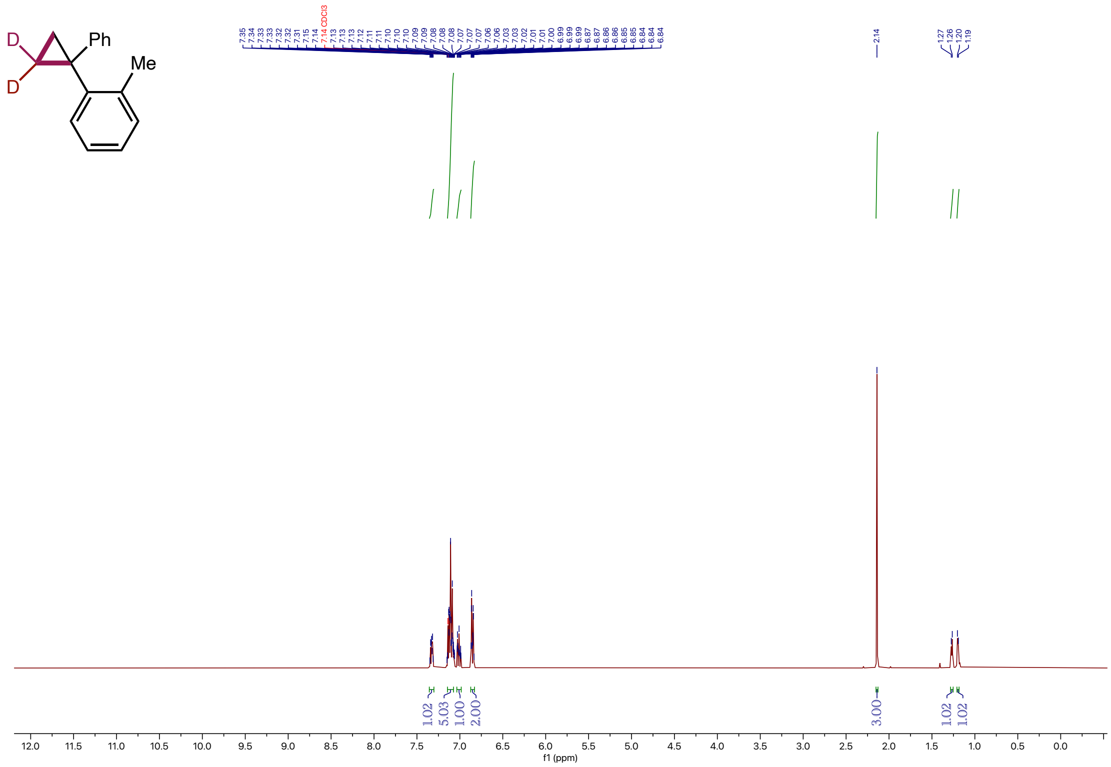

^{13}C NMR Spectrum of **7** (101 MHz, $CDCl_3$)

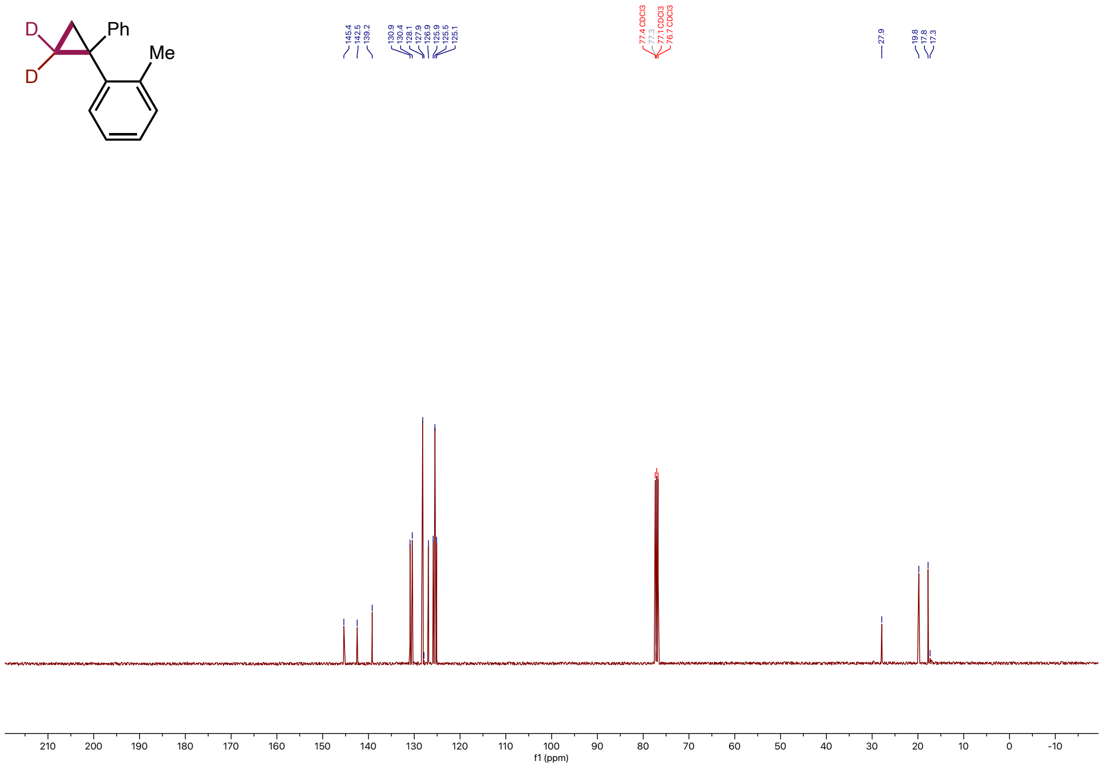

^{1}H NMR Spectrum of **8** (400 MHz, $CDCl_3$)

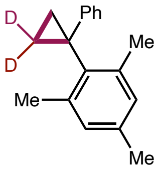

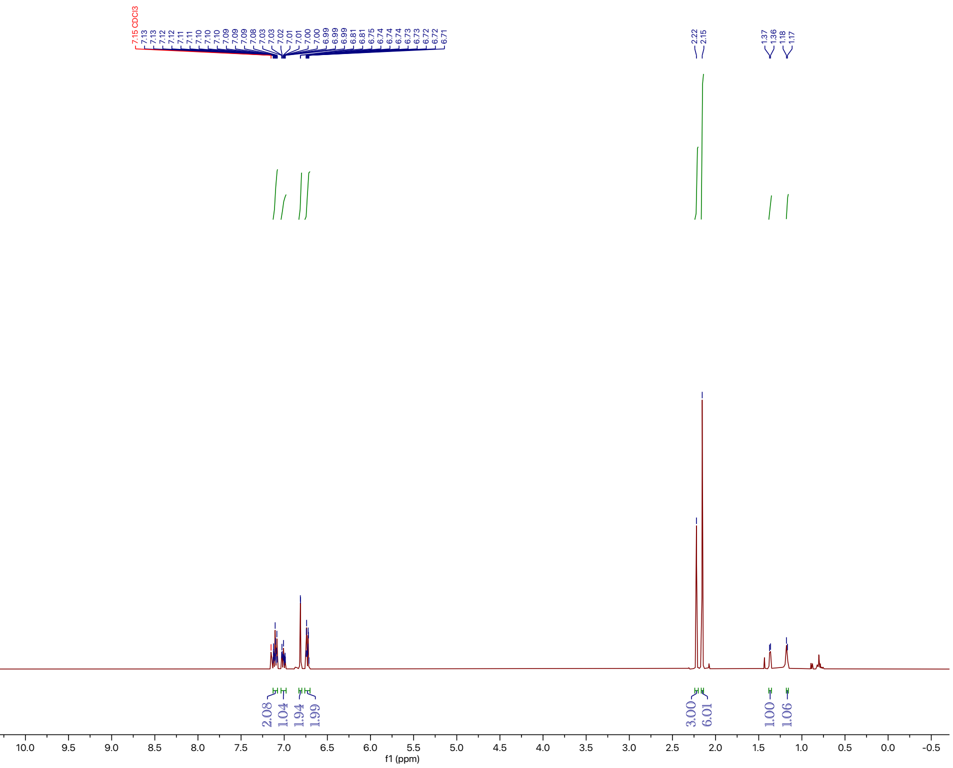

^{13}C NMR Spectrum of **8** (101 MHz, $CDCl_3$)

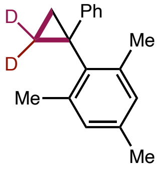

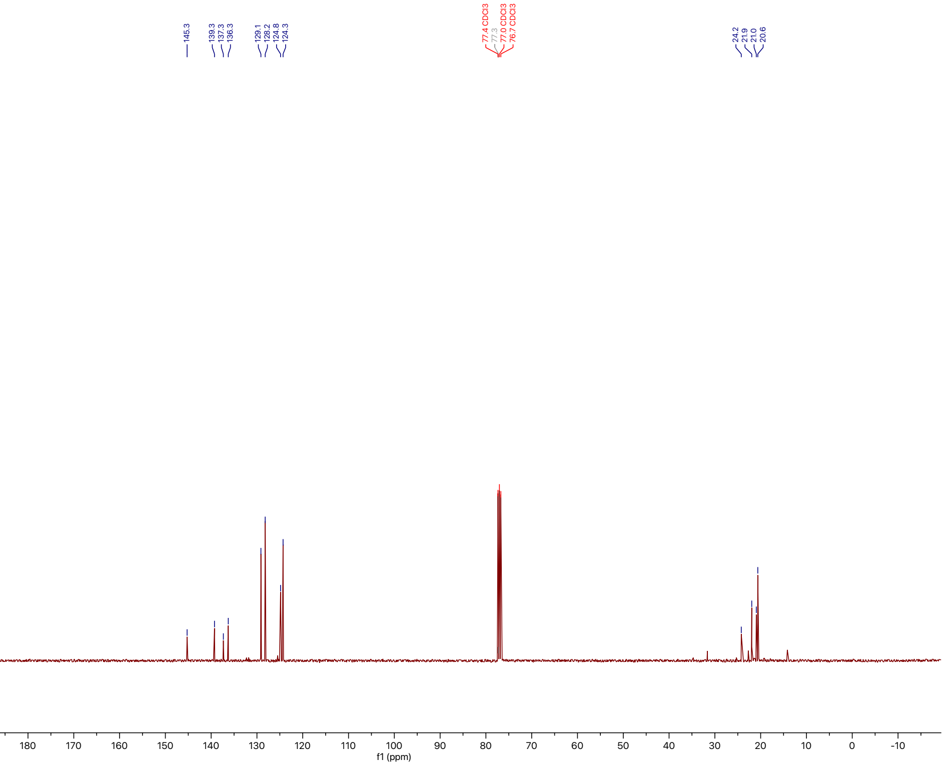

^{1}H NMR Spectrum of **9** (400 MHz, $CDCl_3$)

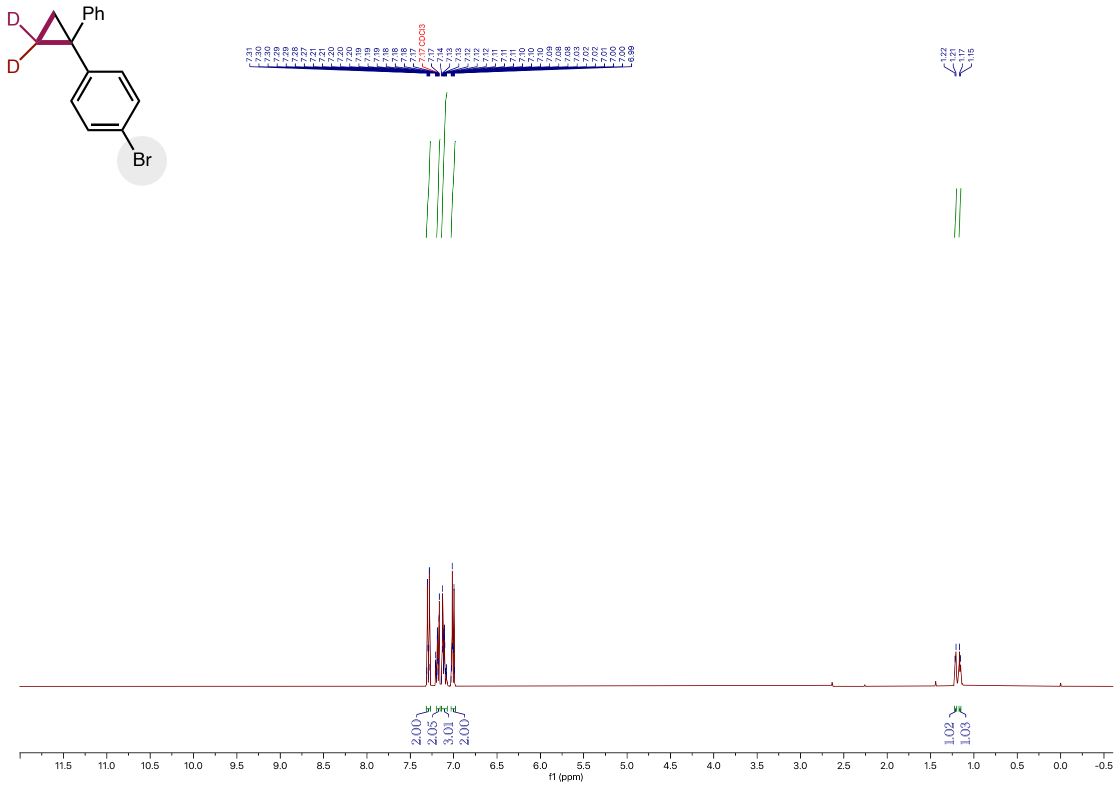

^{13}C NMR Spectrum of **9** (101 MHz, $CDCl_3$)

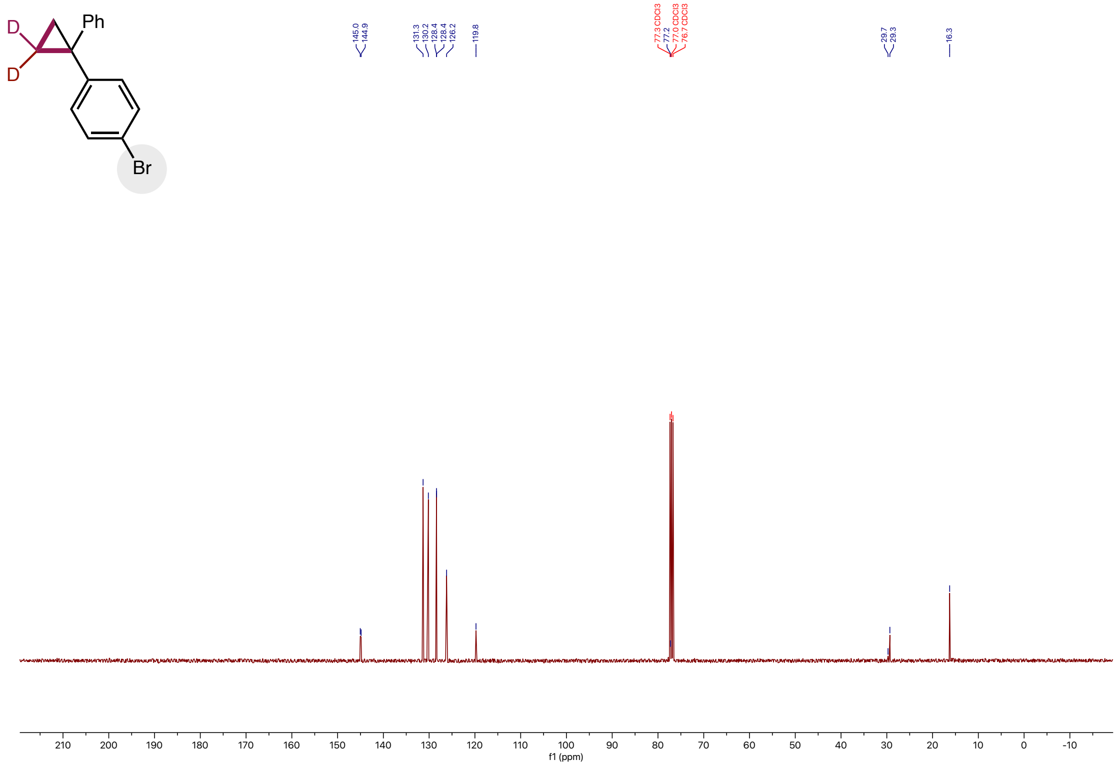

^{1}H NMR Spectrum of **10** (400 MHz, $CDCl_3$)

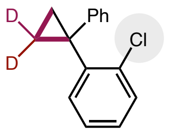

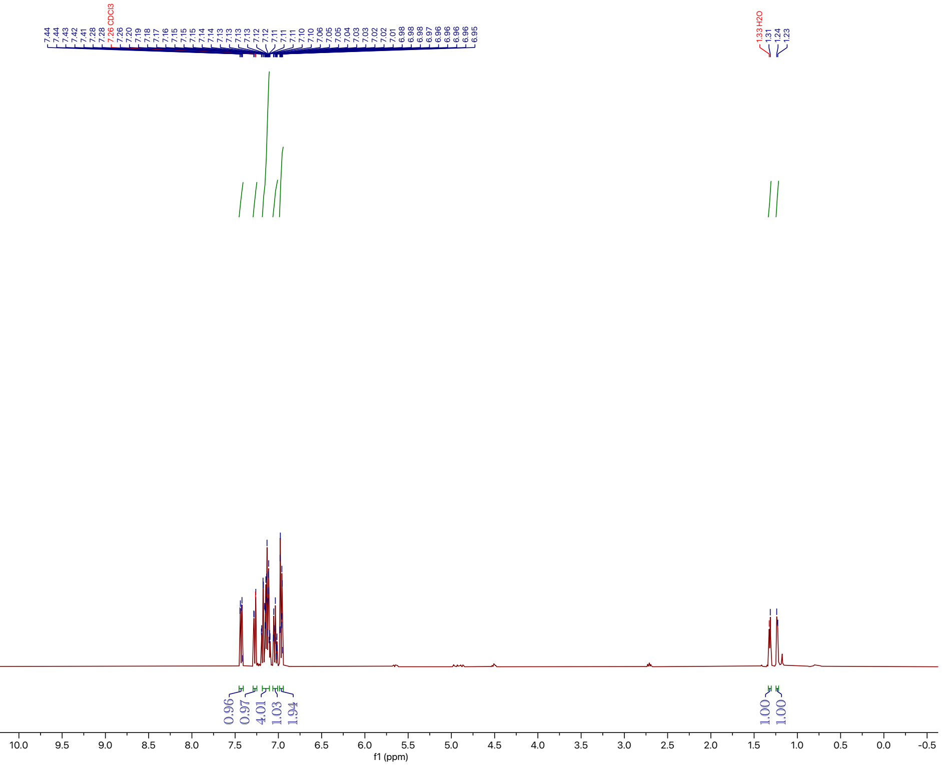

^{13}C NMR Spectrum of **10** (101 MHz, $CDCl_3$)

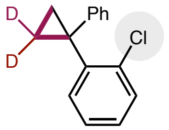

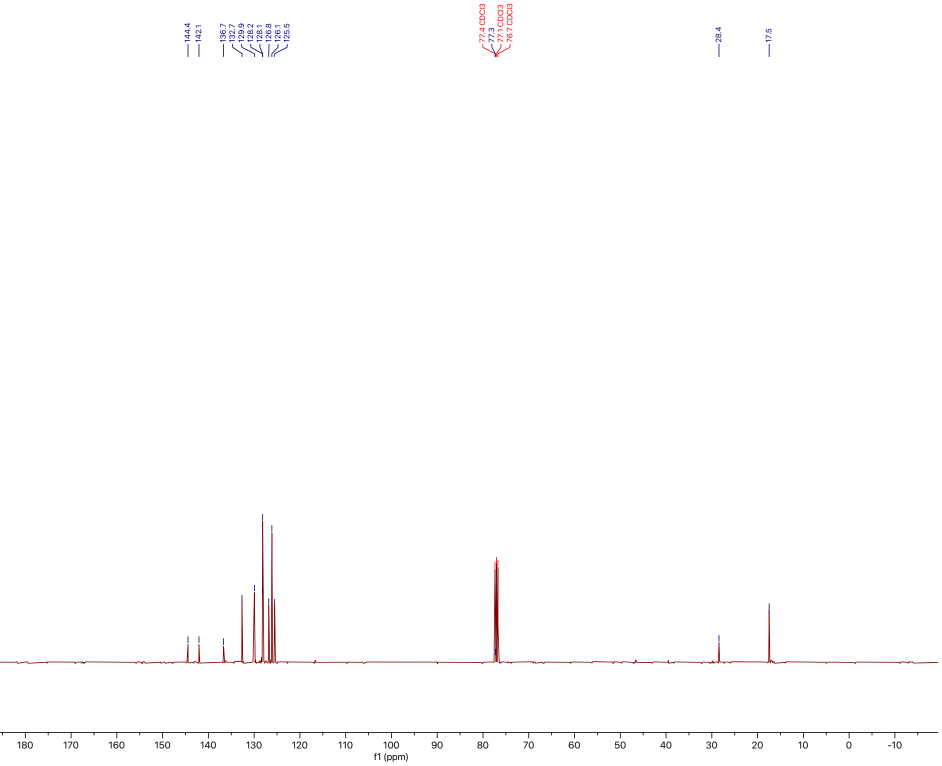

^{1}H NMR Spectrum of **11** (400 MHz, $CDCl_3$)

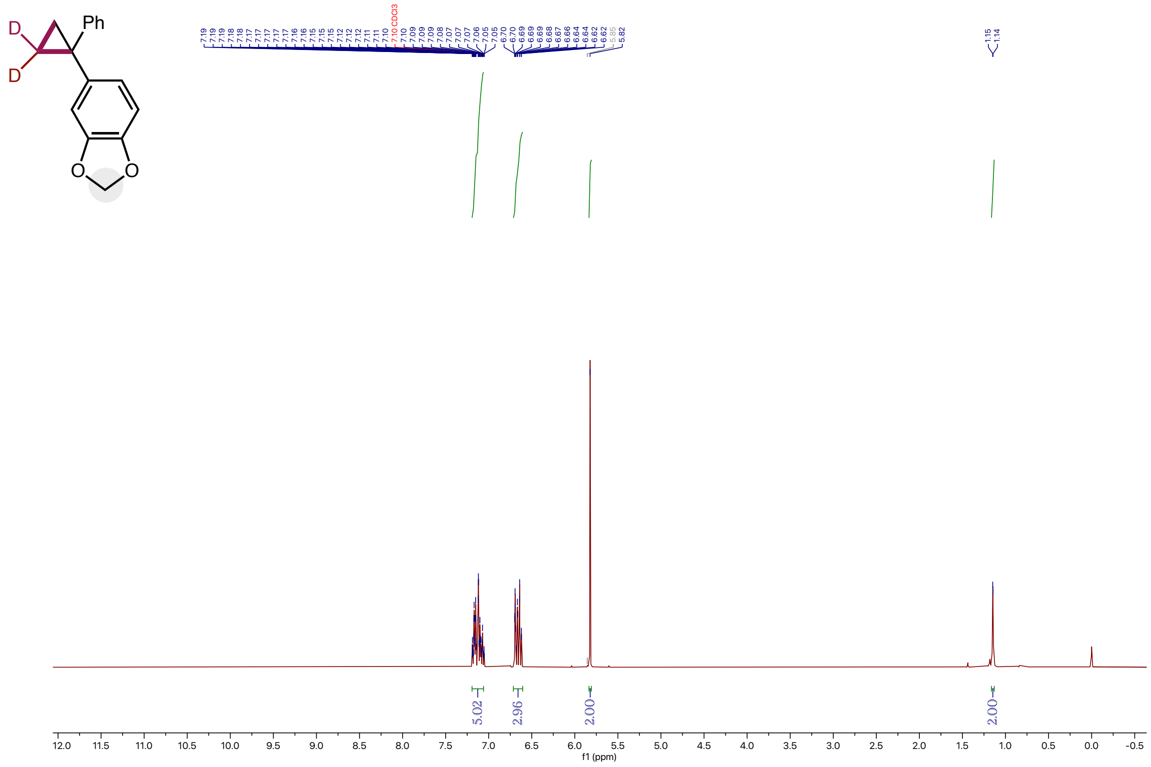

^{13}C NMR Spectrum of **11** (101 MHz, $CDCl_3$)

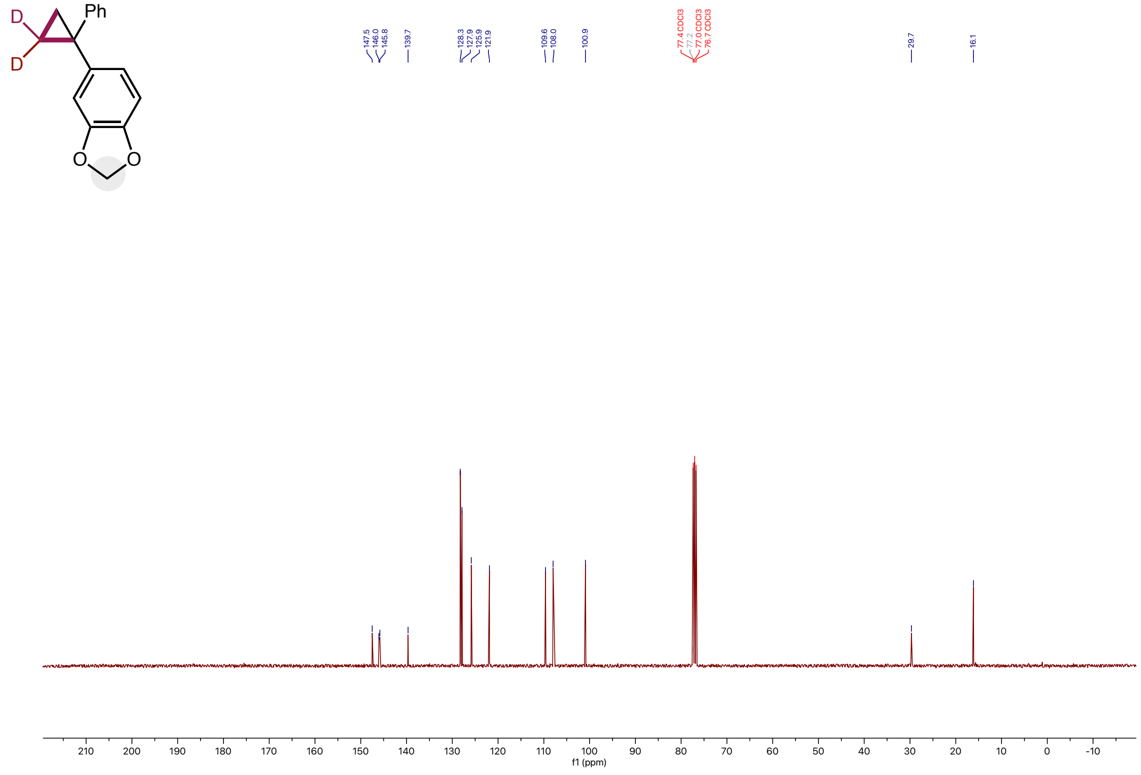

^{1}H NMR Spectrum of **12** (400 MHz, $CDCl_3$)

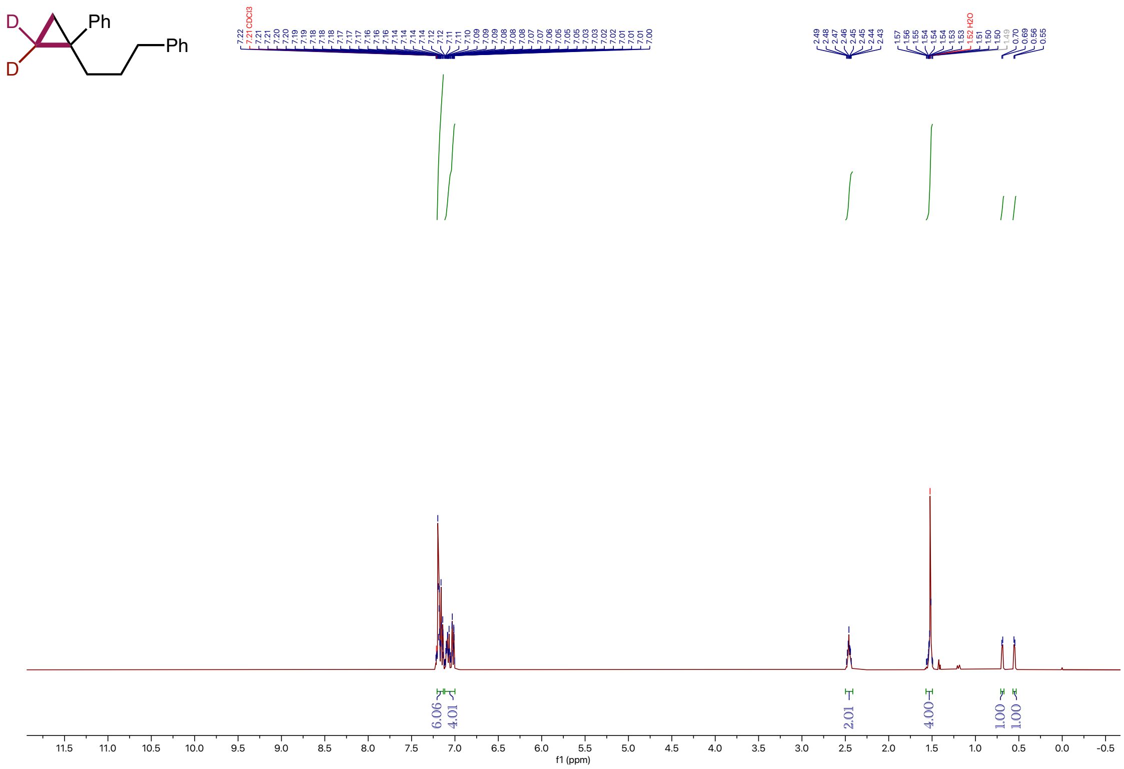

^{13}C NMR Spectrum of **12** (101 MHz, $CDCl_3$)

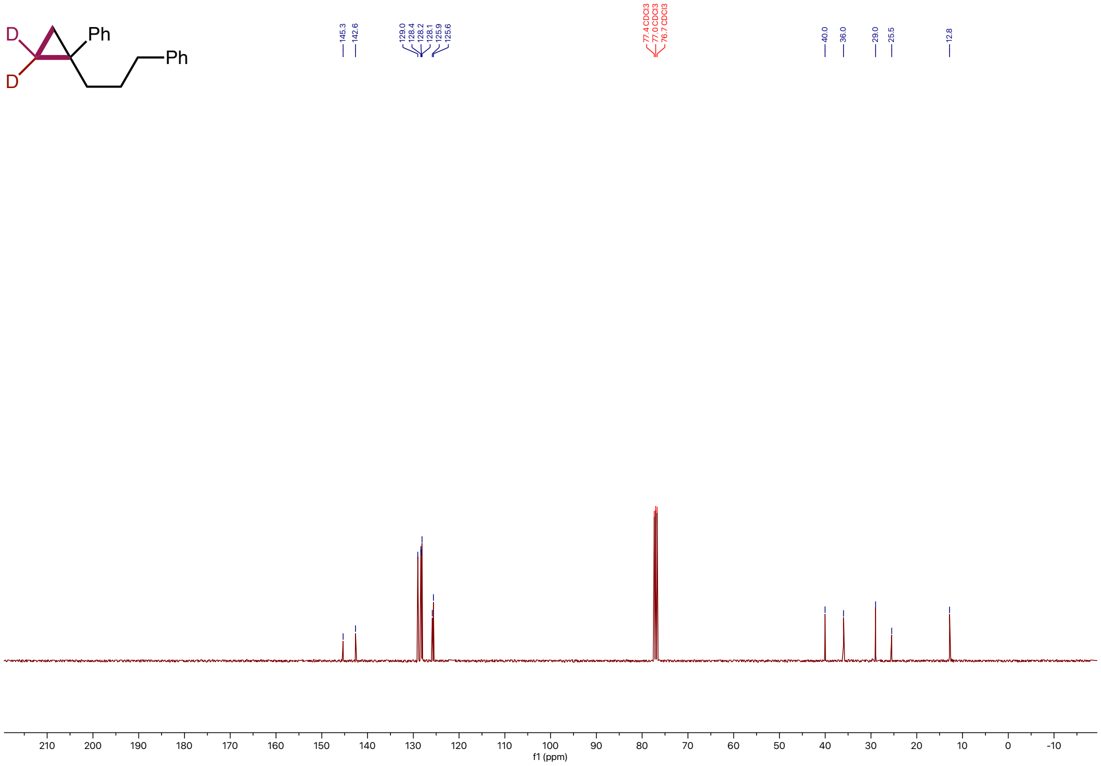

^{1}H NMR Spectrum of **13** (400 MHz, $CDCl_3$)

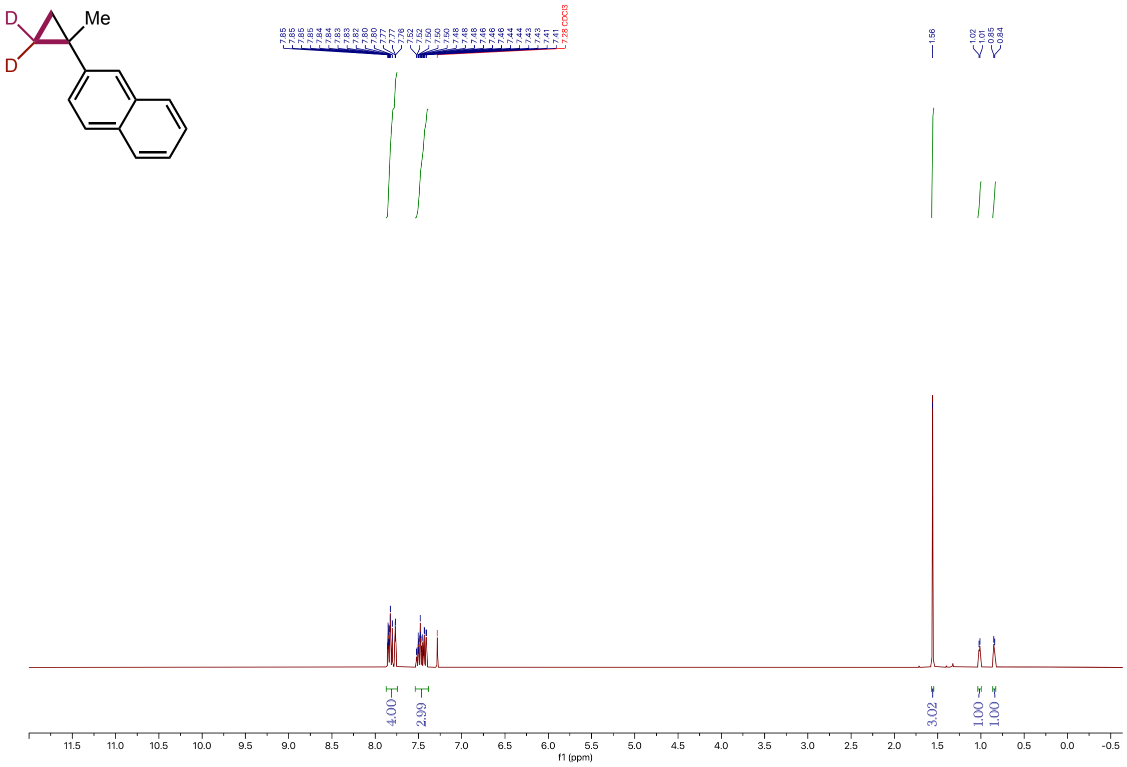

^{13}C NMR Spectrum of **13** (101 MHz, $CDCl_3$)

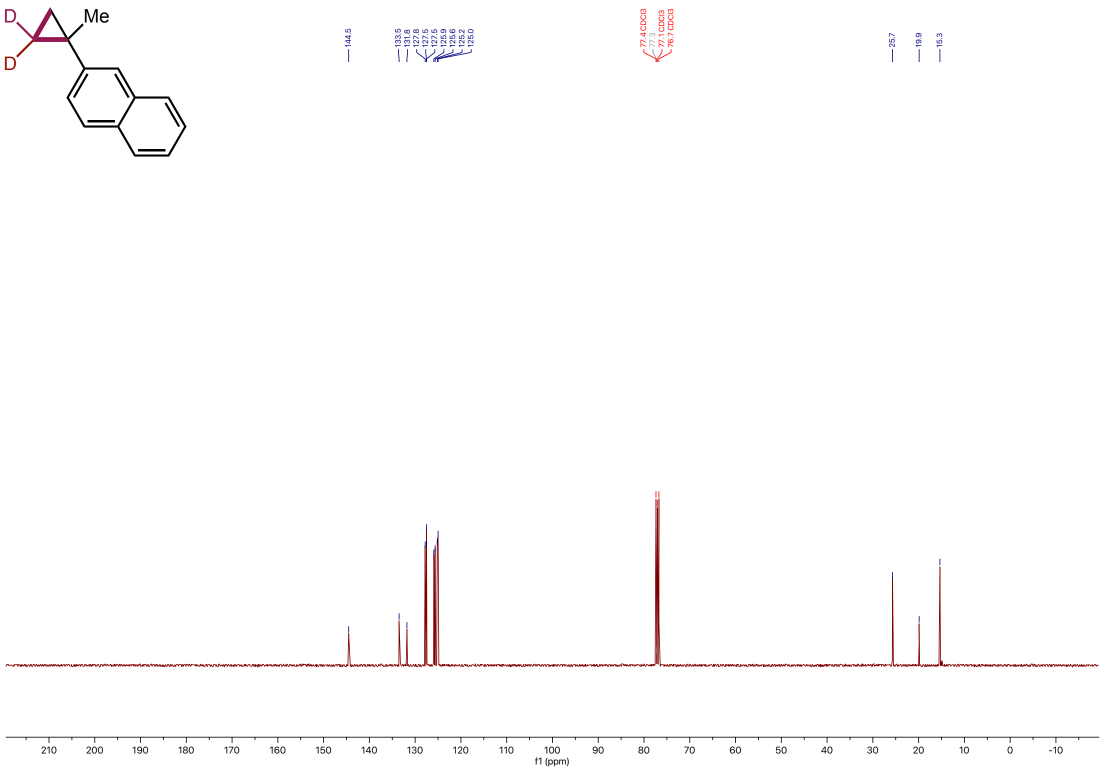

^{1}H NMR Spectrum of **14** (400 MHz, $CDCl_3$)

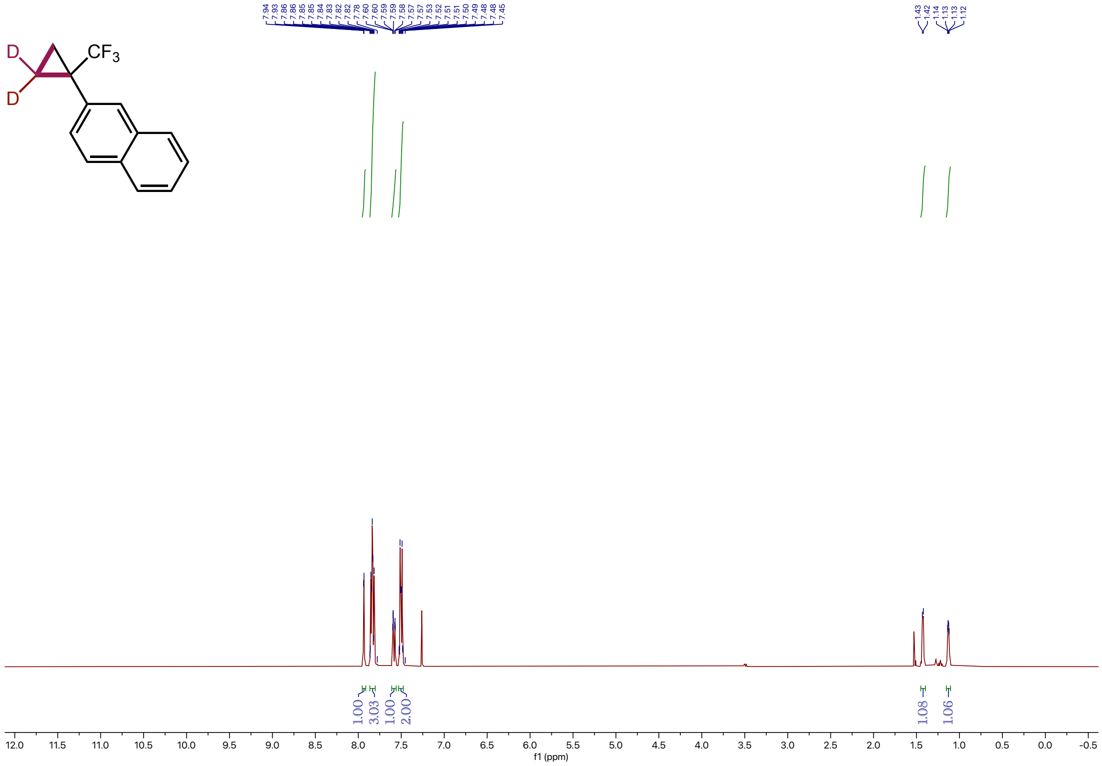

^{13}C NMR Spectrum of **14** (101 MHz, $CDCl_3$)

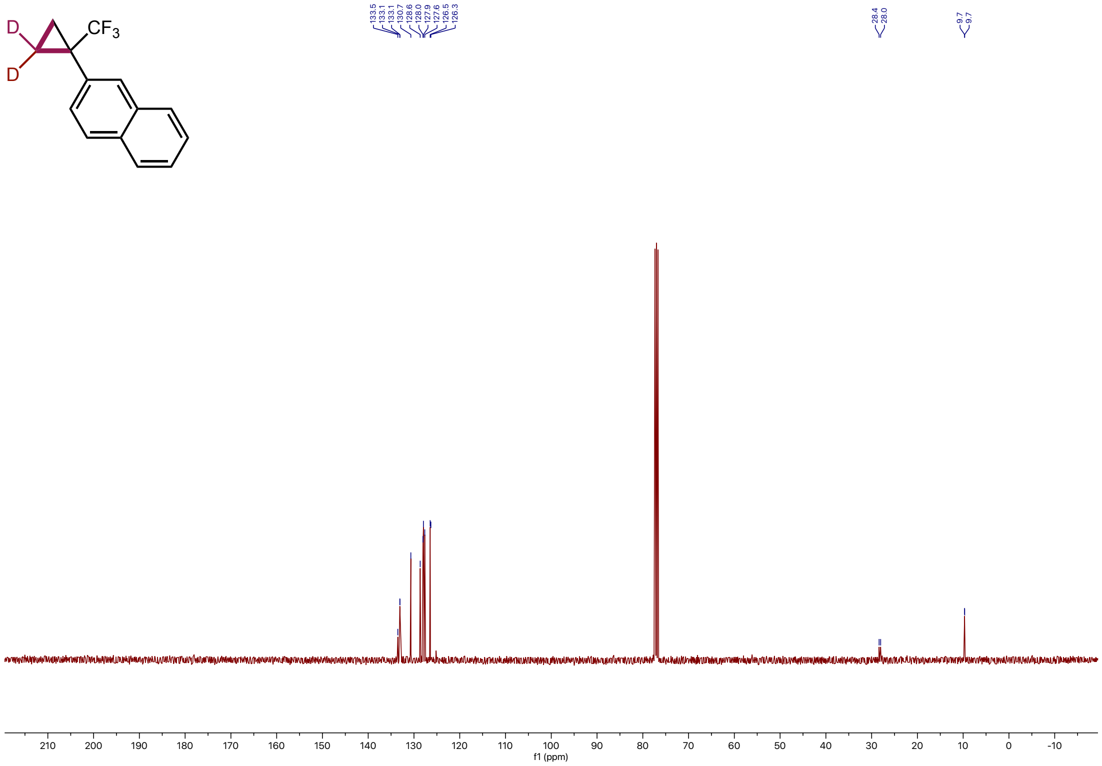

^{19}F NMR Spectrum of **14** (376 MHz, $CDCl_3$)

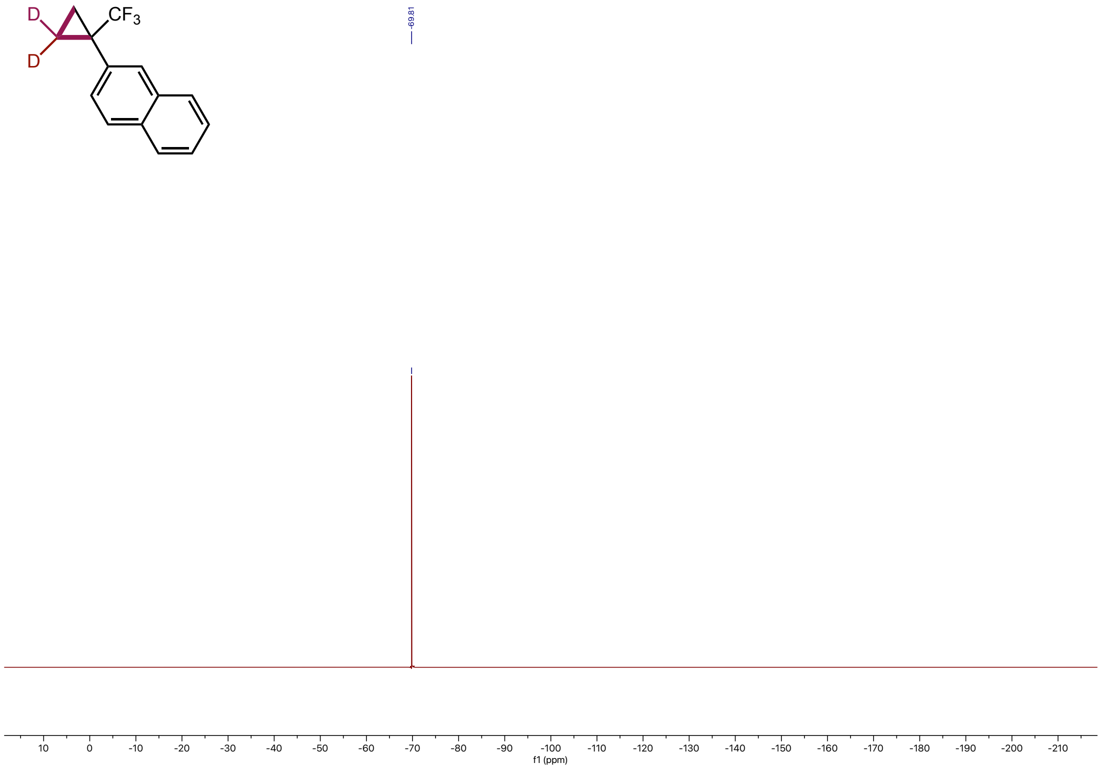

^{1}H NMR Spectrum of **15** (400 MHz, $CDCl_3$)

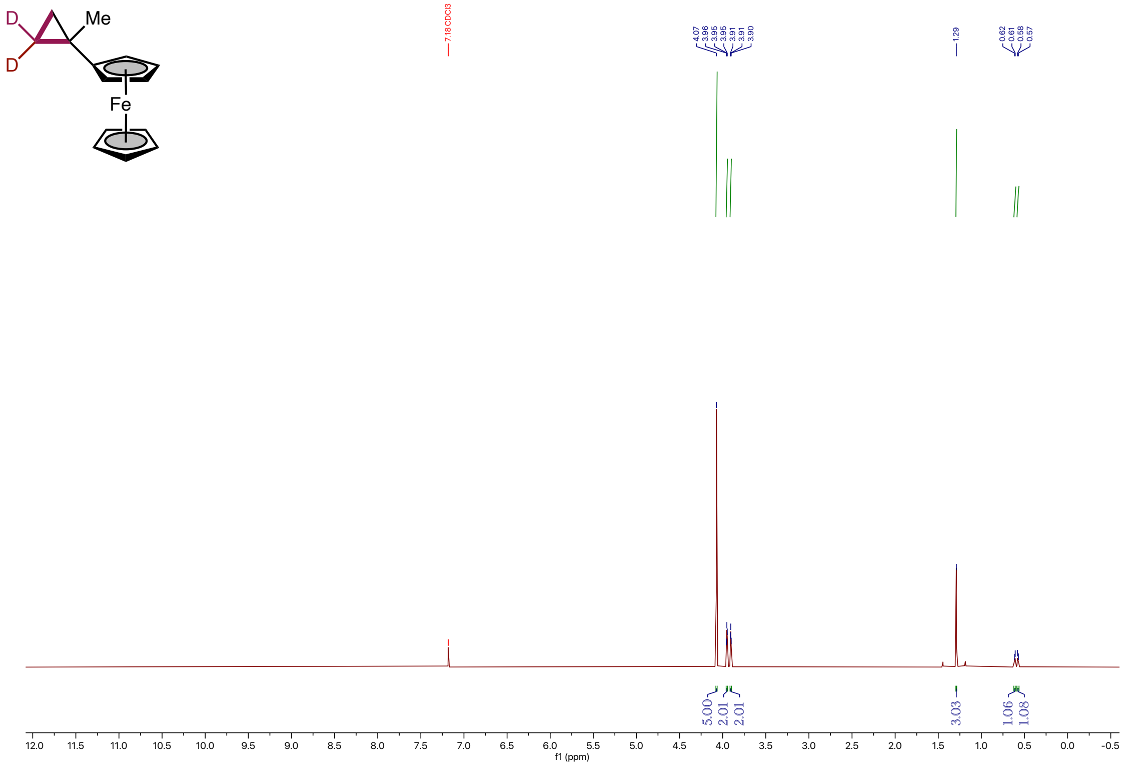

^{13}C NMR Spectrum of **15** (101 MHz, $CDCl_3$)

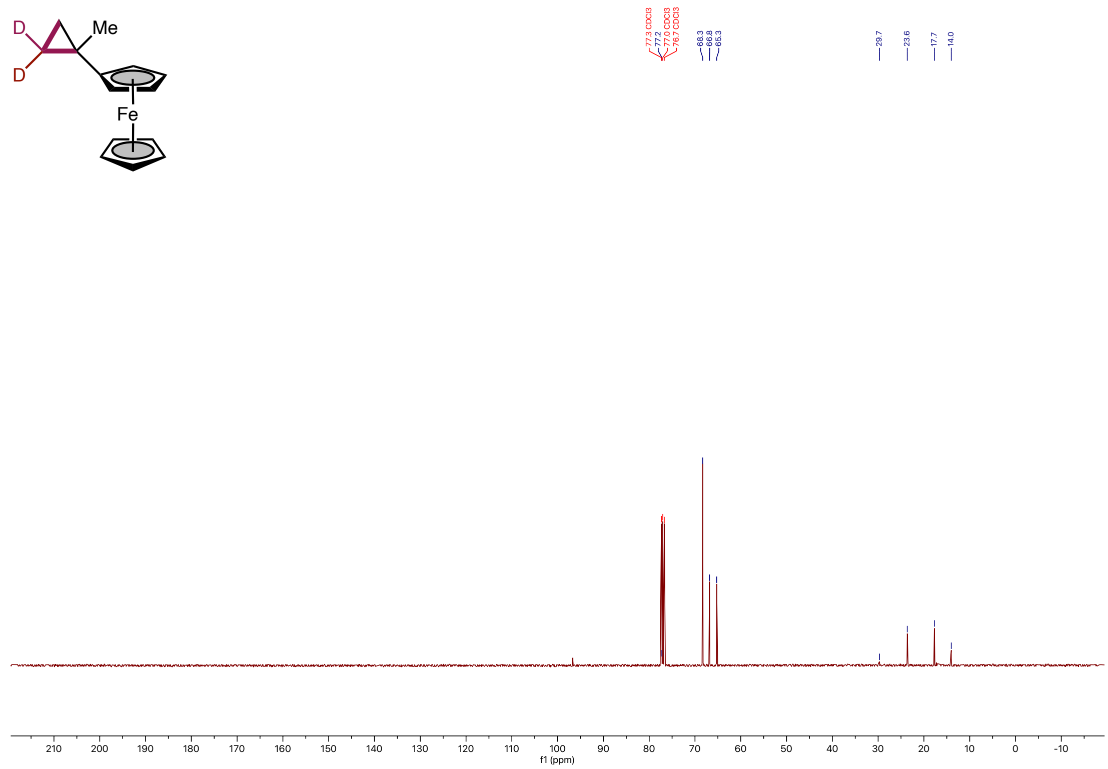

^{1}H NMR Spectrum of **16** (400 MHz, $CDCl_3$)

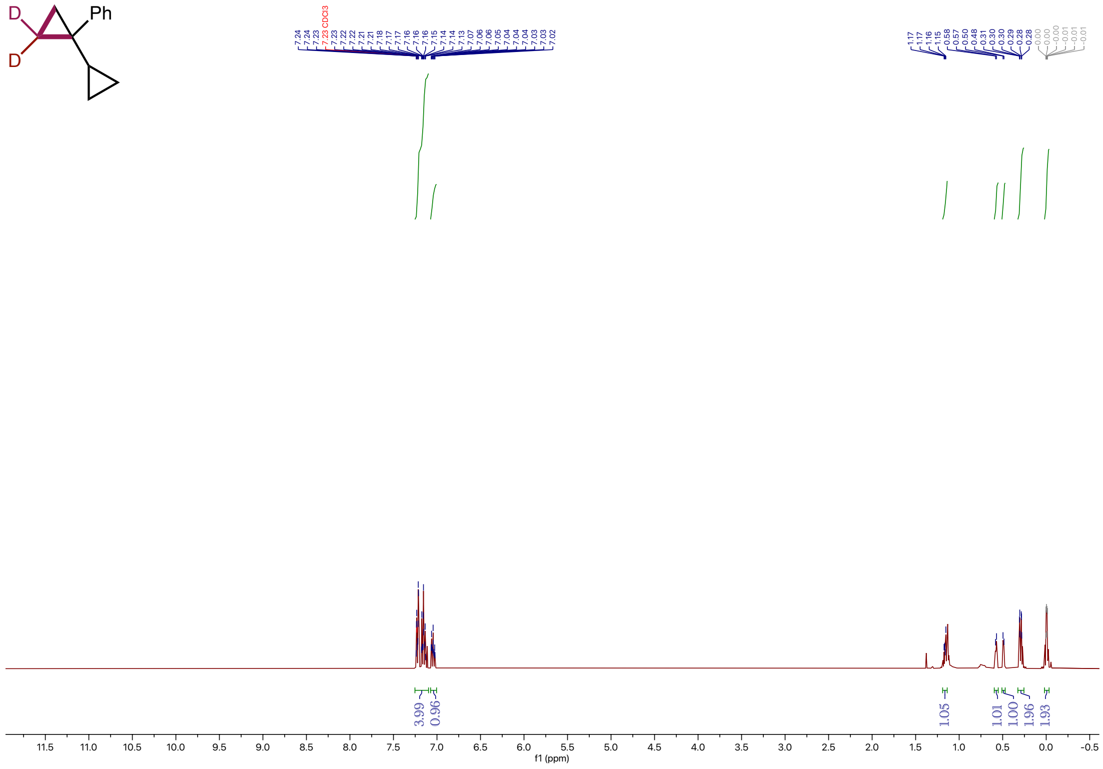

^{13}C NMR Spectrum of **16** (101 MHz, $CDCl_3$)

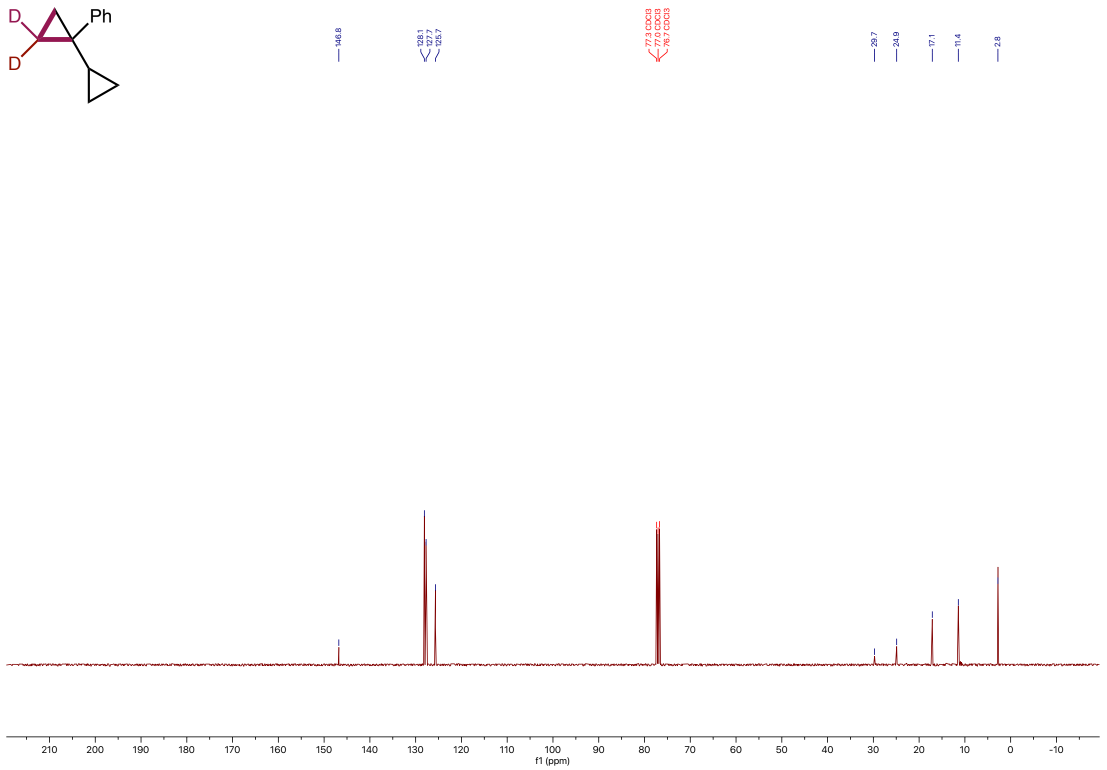

^{1}H NMR Spectrum of **17** (400 MHz, $CDCl_3$)

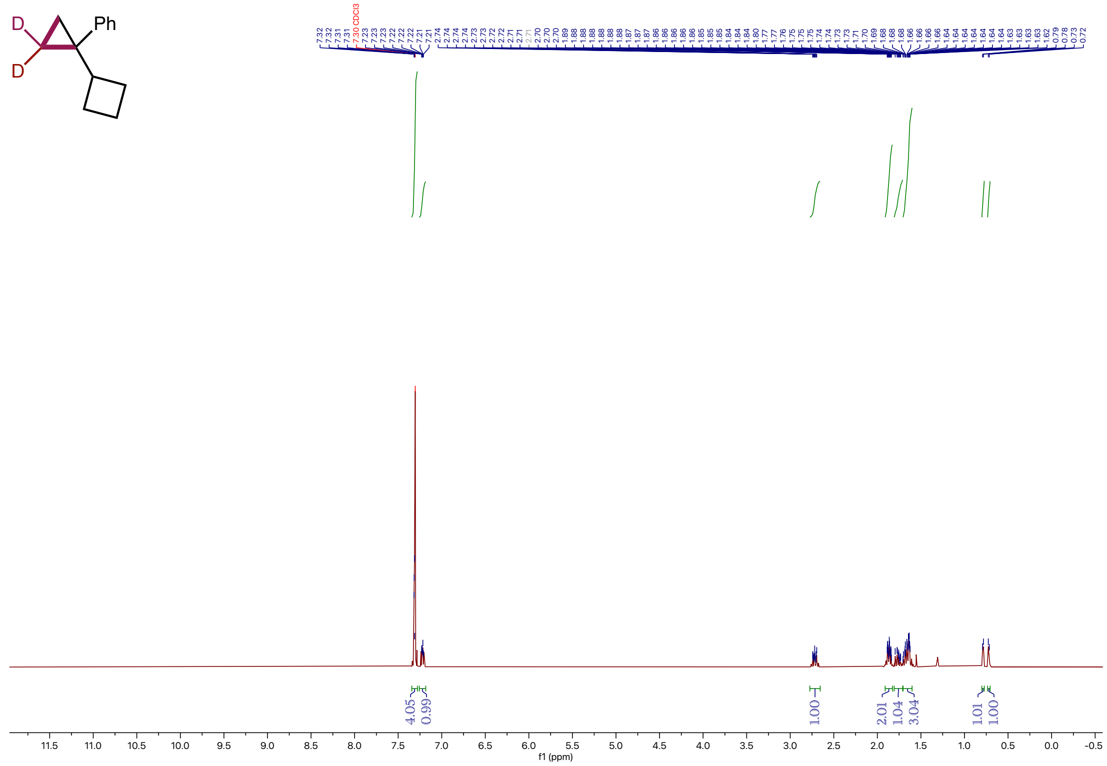

^{13}C NMR Spectrum of **17** (101 MHz, $CDCl_3$)

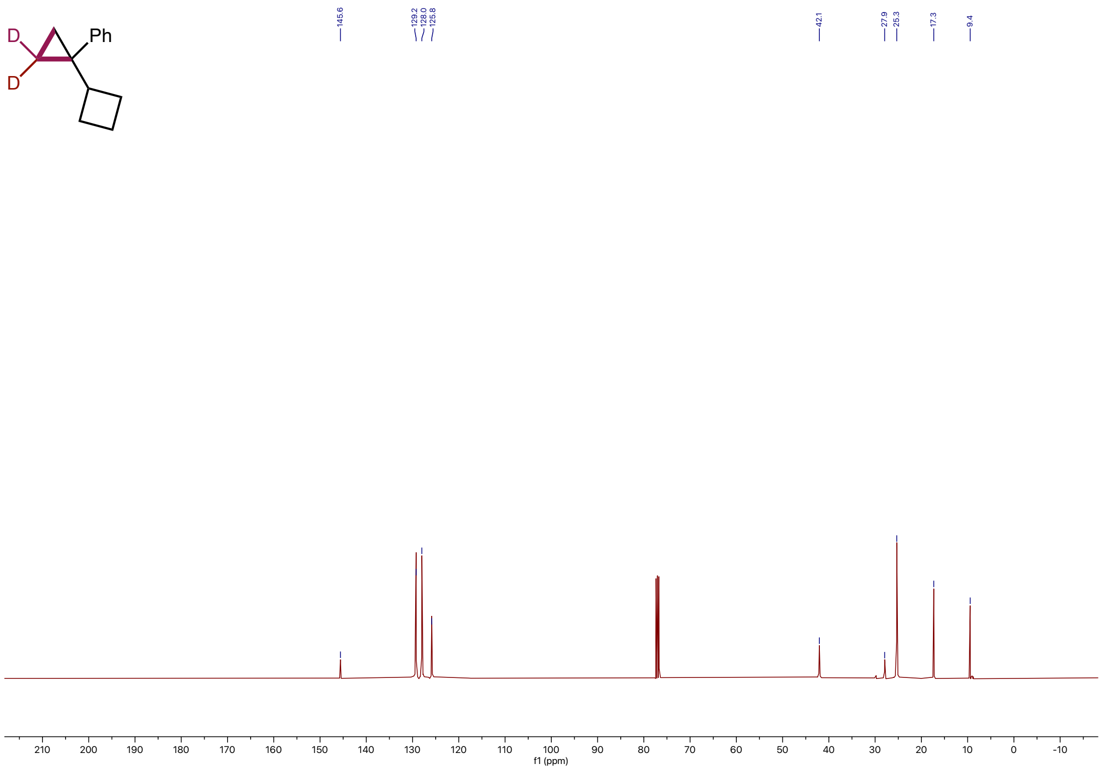

^{1}H NMR Spectrum of **18** (400 MHz, $CDCl_3$)

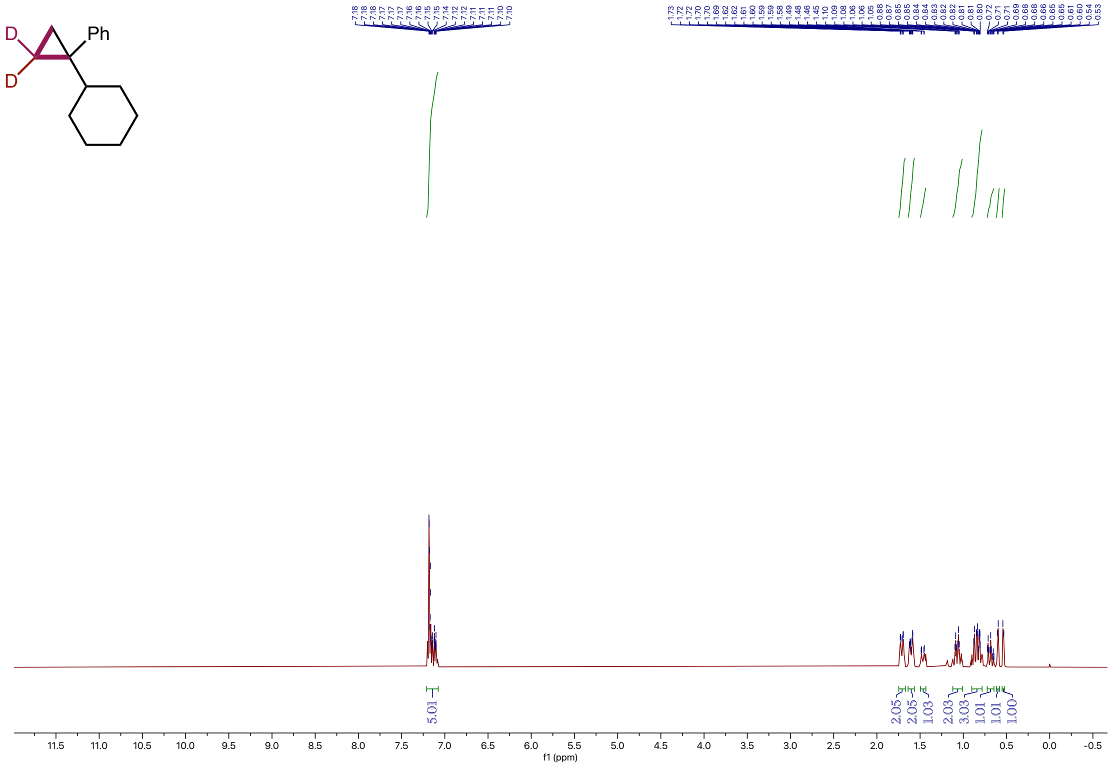

^{13}C NMR Spectrum of **18** (101 MHz, $CDCl_3$)

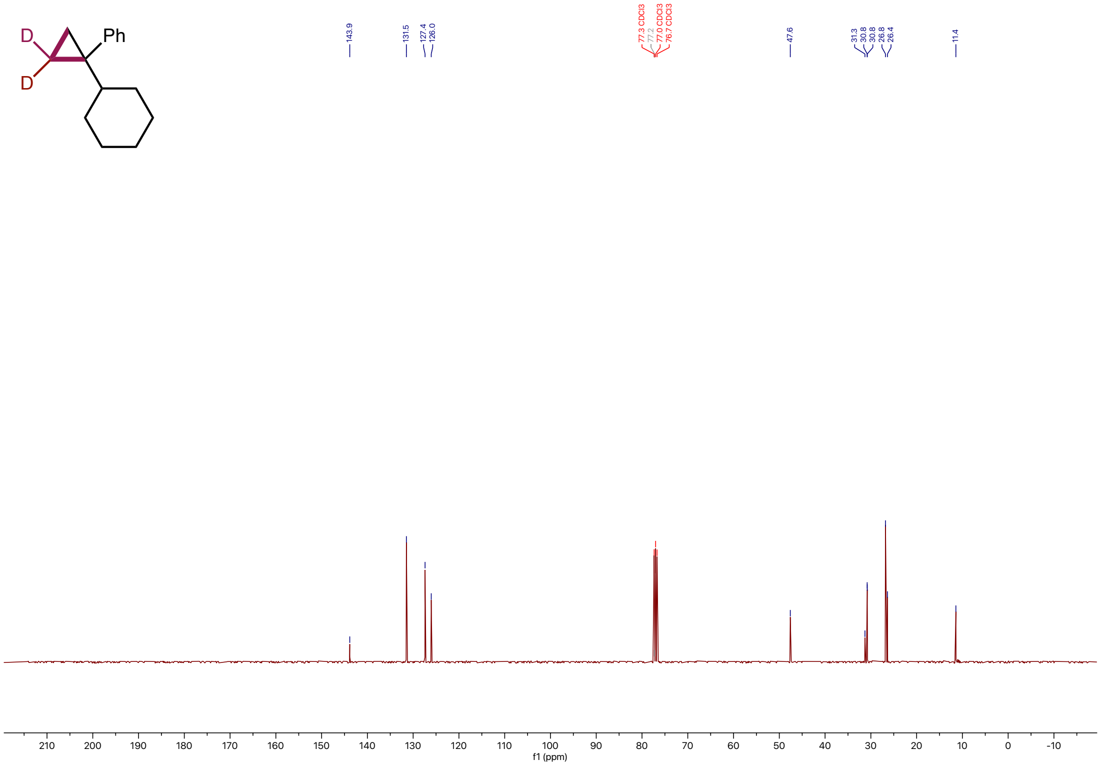

^{1}H NMR Spectrum of **19** (400 MHz, $CDCl_3$)

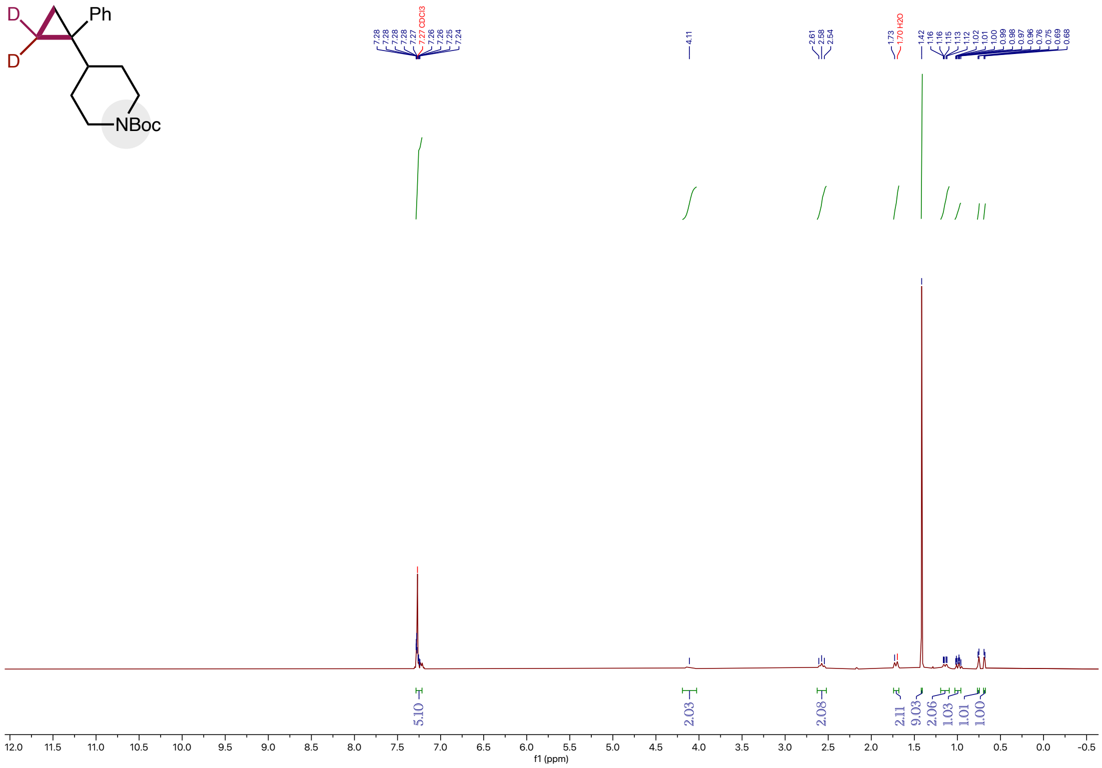

^{13}C NMR Spectrum of **19** (101 MHz, $CDCl_3$)

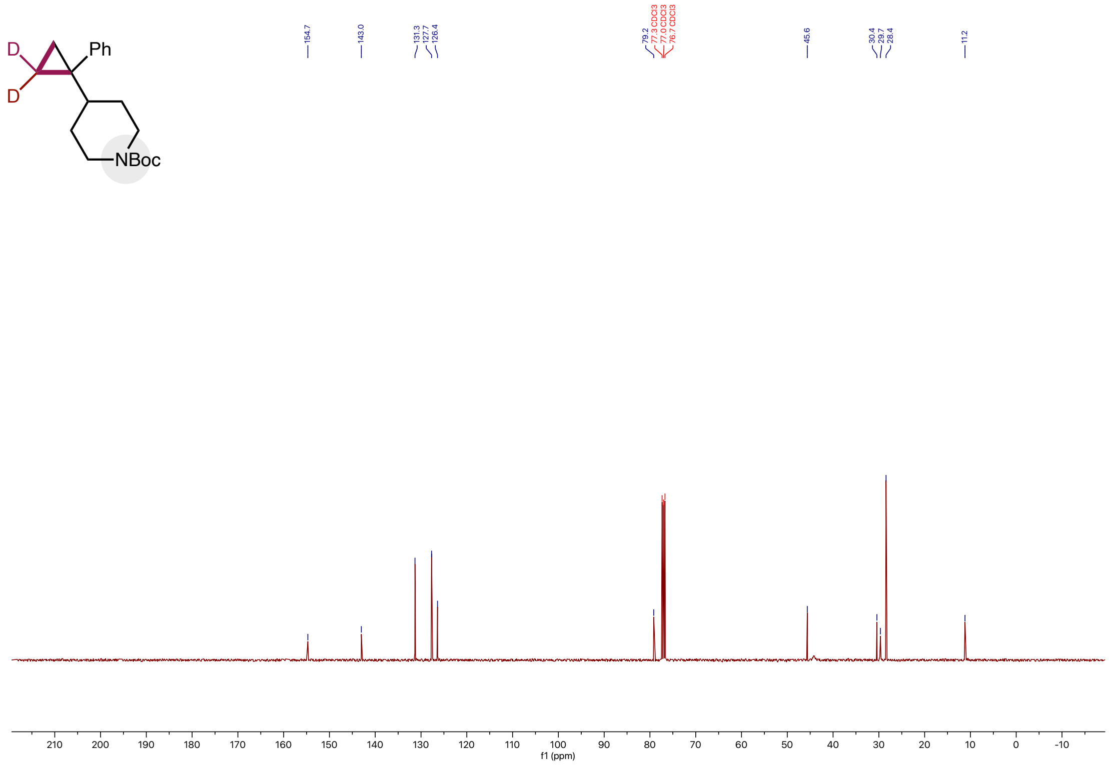

^{1}H NMR Spectrum of **20** (400 MHz, $CDCl_3$)

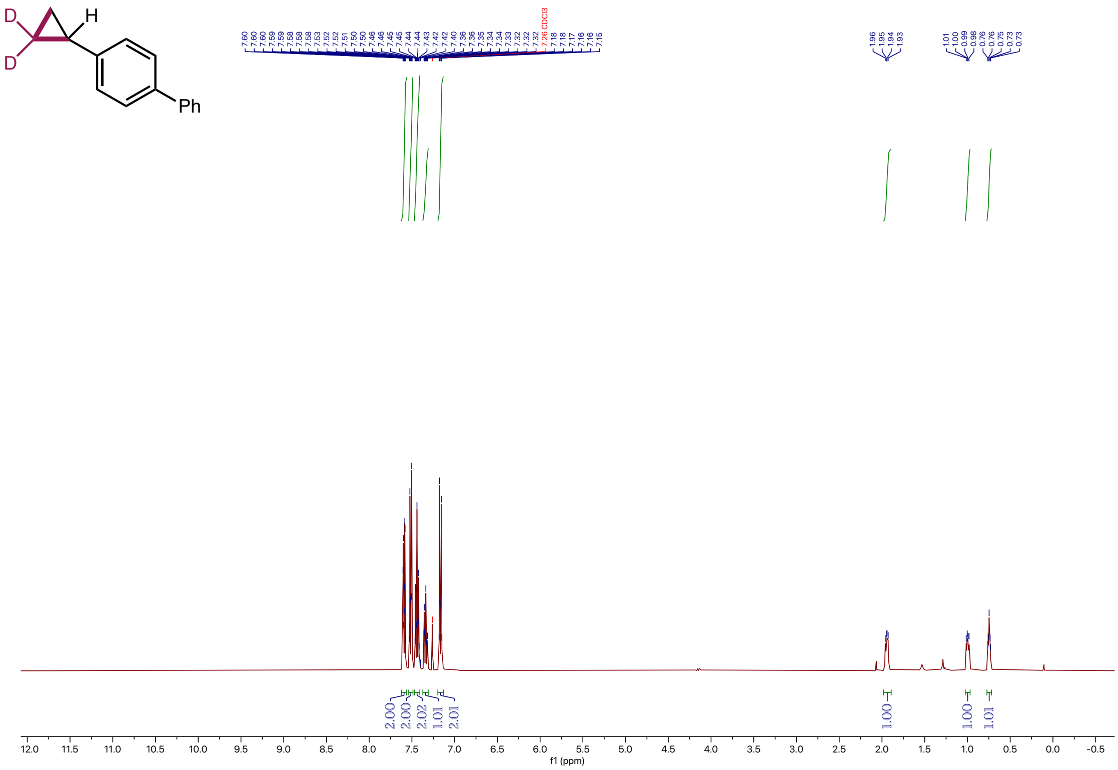

^{13}C NMR Spectrum of **20** (101 MHz, $CDCl_3$)

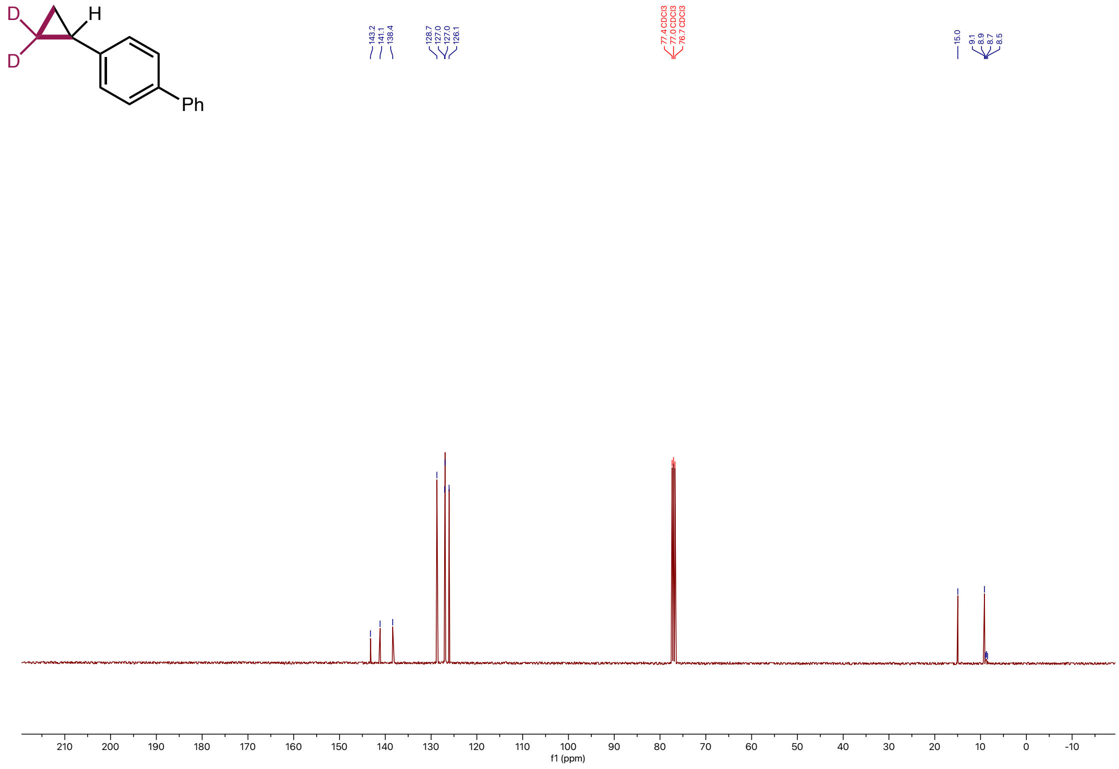

^{1}H NMR Spectrum of **21** (400 MHz, $CDCl_3$)

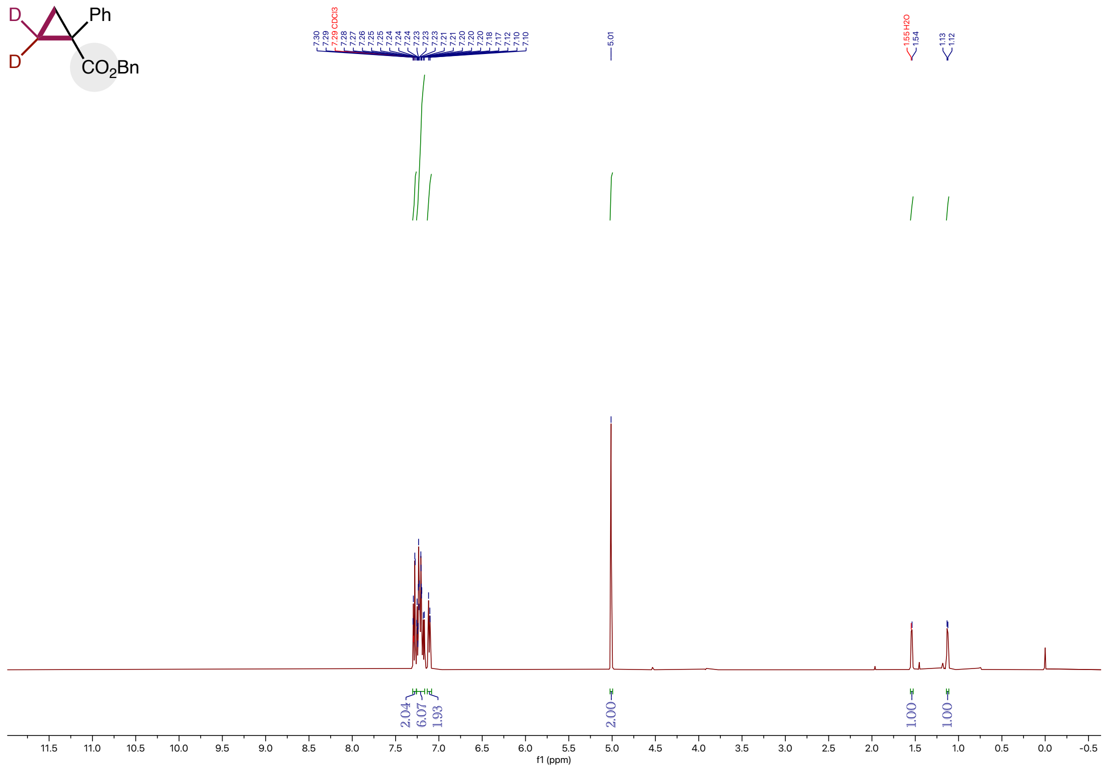

^{13}C NMR Spectrum of **21** (101 MHz, $CDCl_3$)

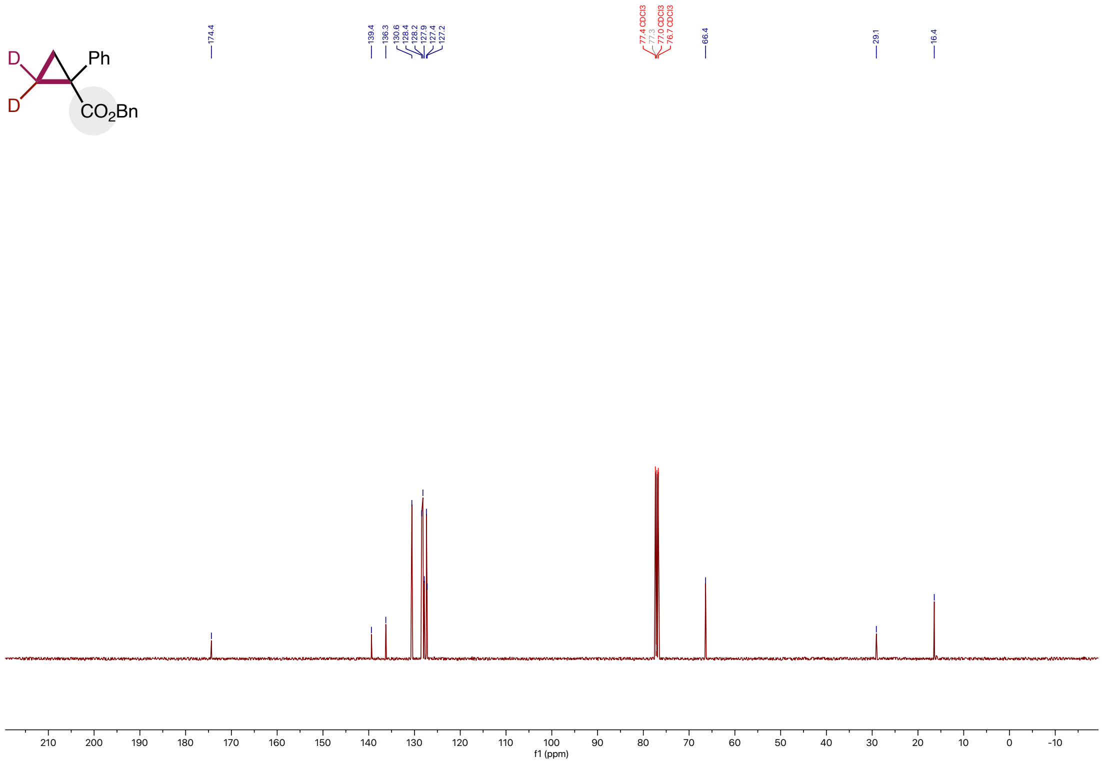

^{1}H NMR Spectrum of **22** (400 MHz, $CDCl_3$)

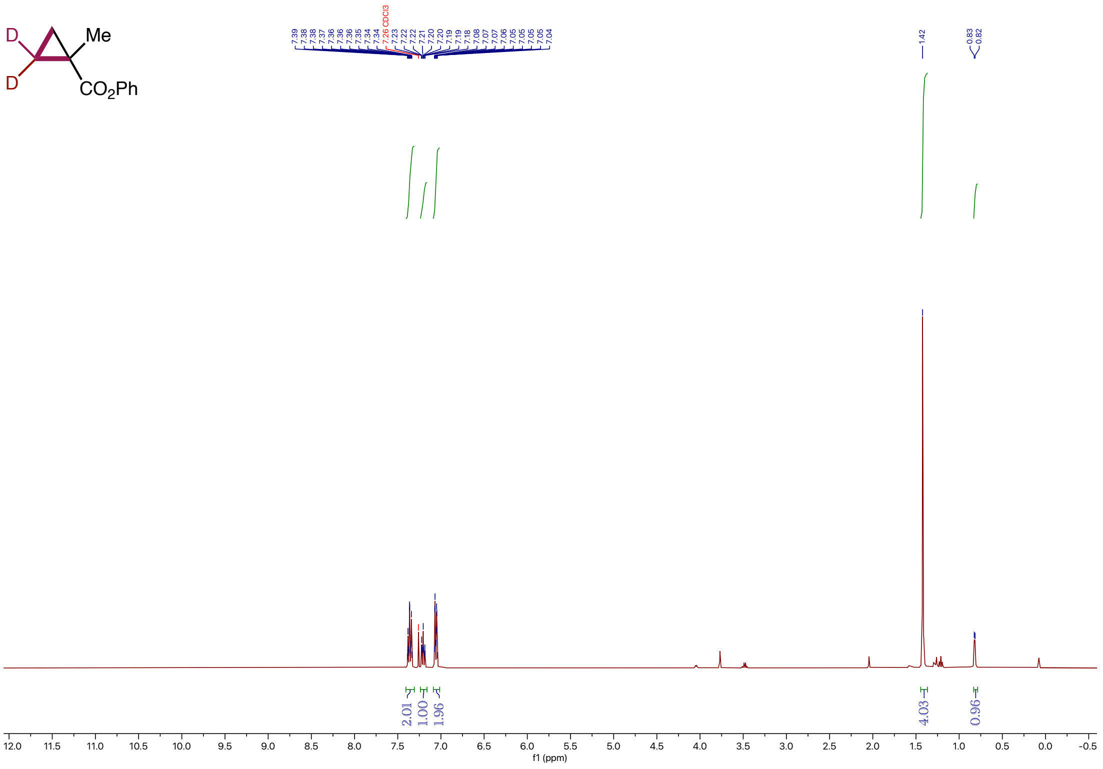

^{13}C NMR Spectrum of **22** (101 MHz, $CDCl_3$)

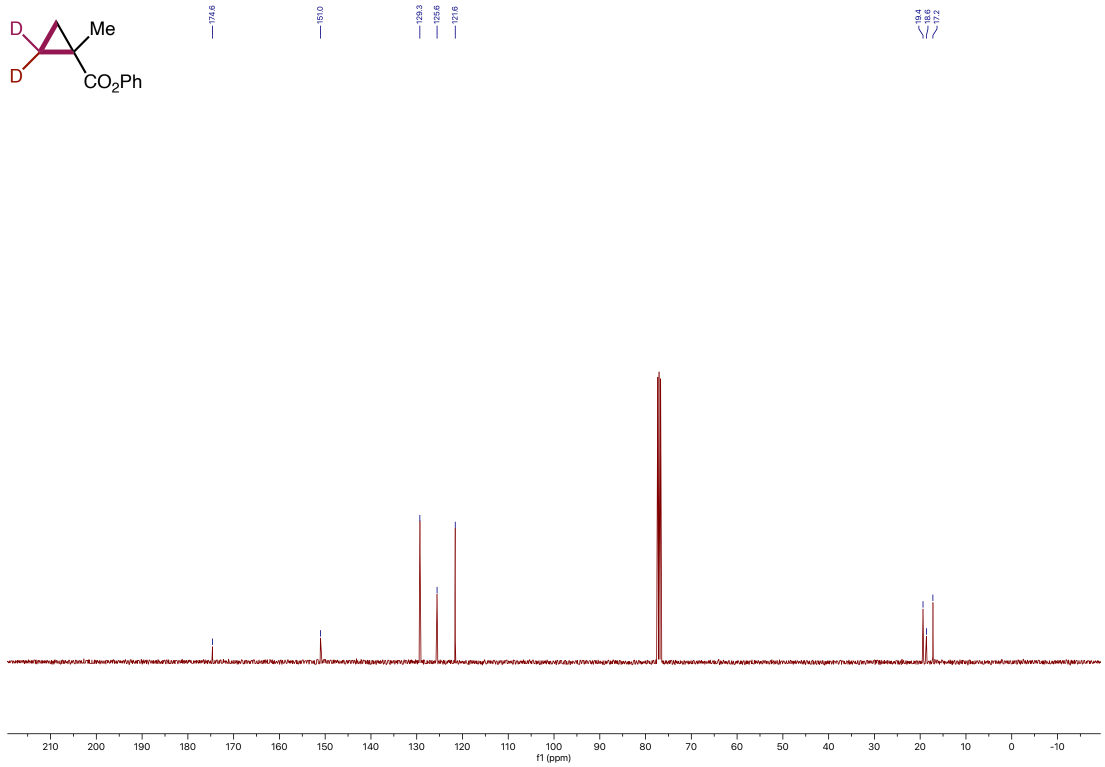

^{1}H NMR Spectrum of **23** (400 MHz, $CDCl_3$)

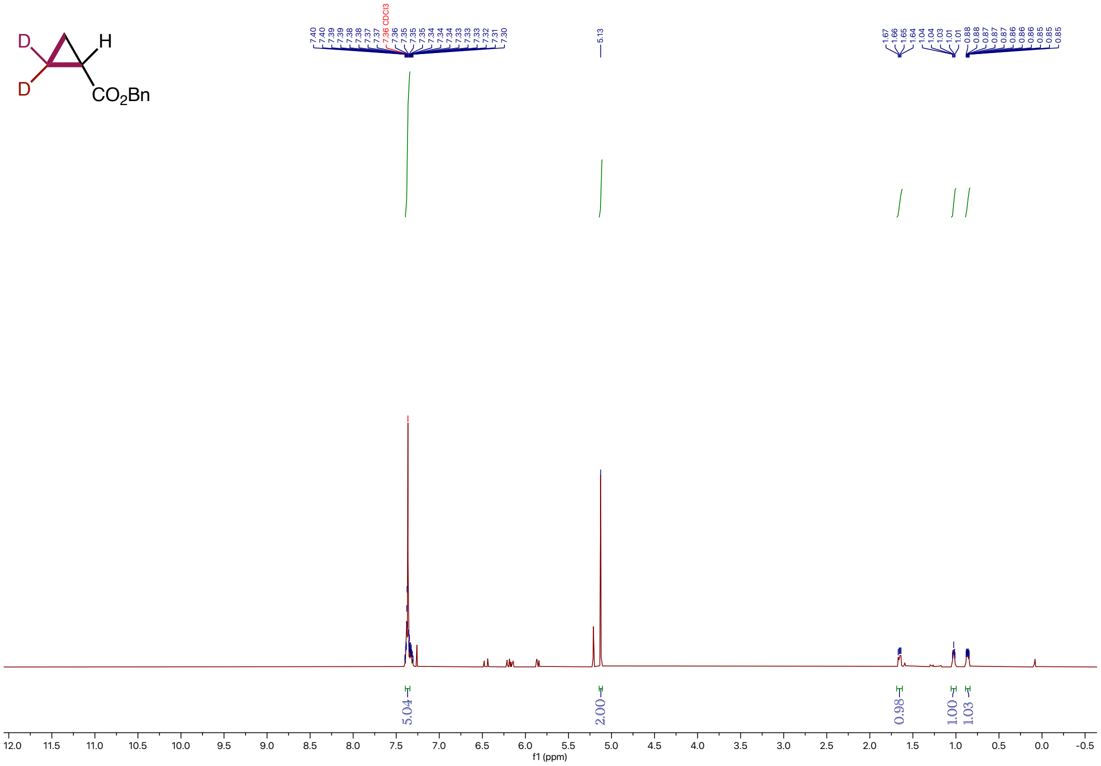

^{13}C NMR Spectrum of **23** (101 MHz, $CDCl_3$)

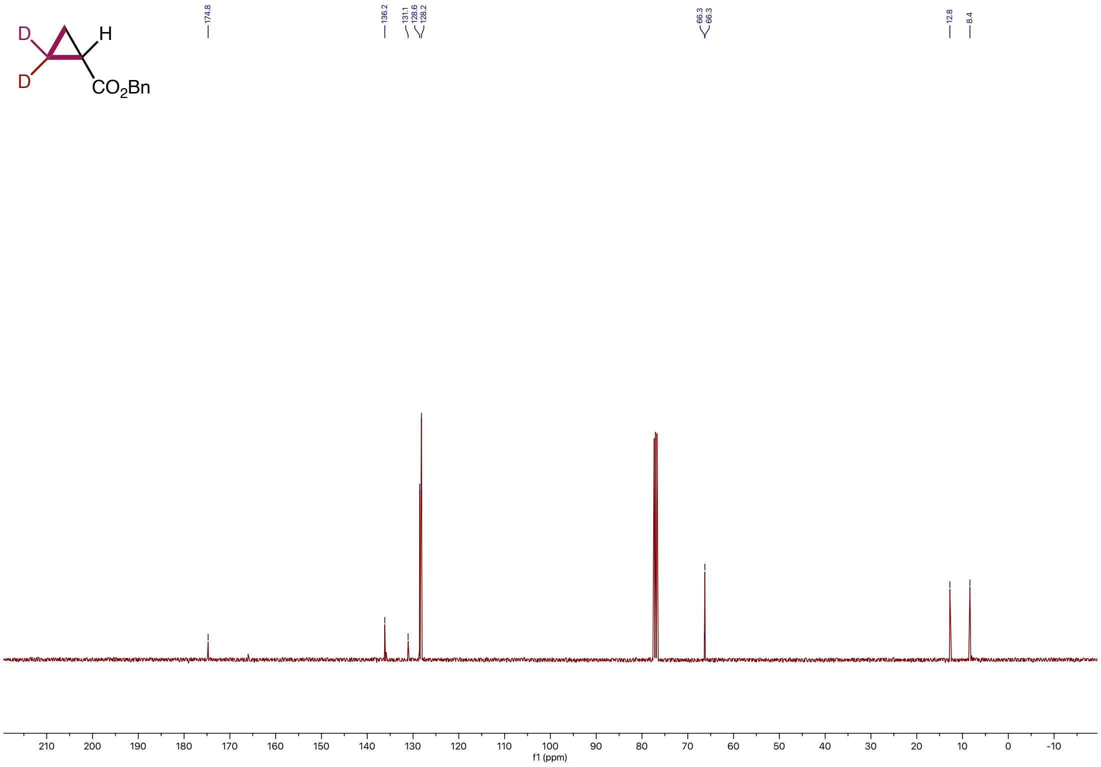

^{1}H NMR Spectrum of **24** (400 MHz, $CDCl_3$)

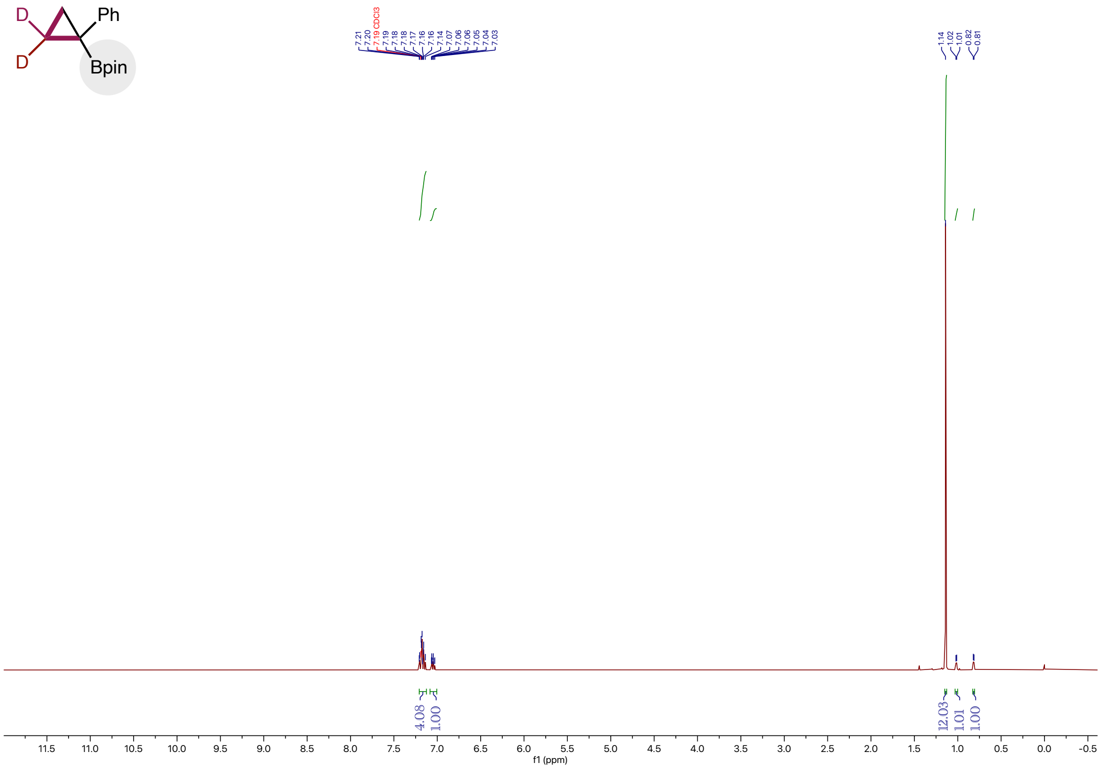

^{13}C NMR Spectrum of **24** (101 MHz, $CDCl_3$)

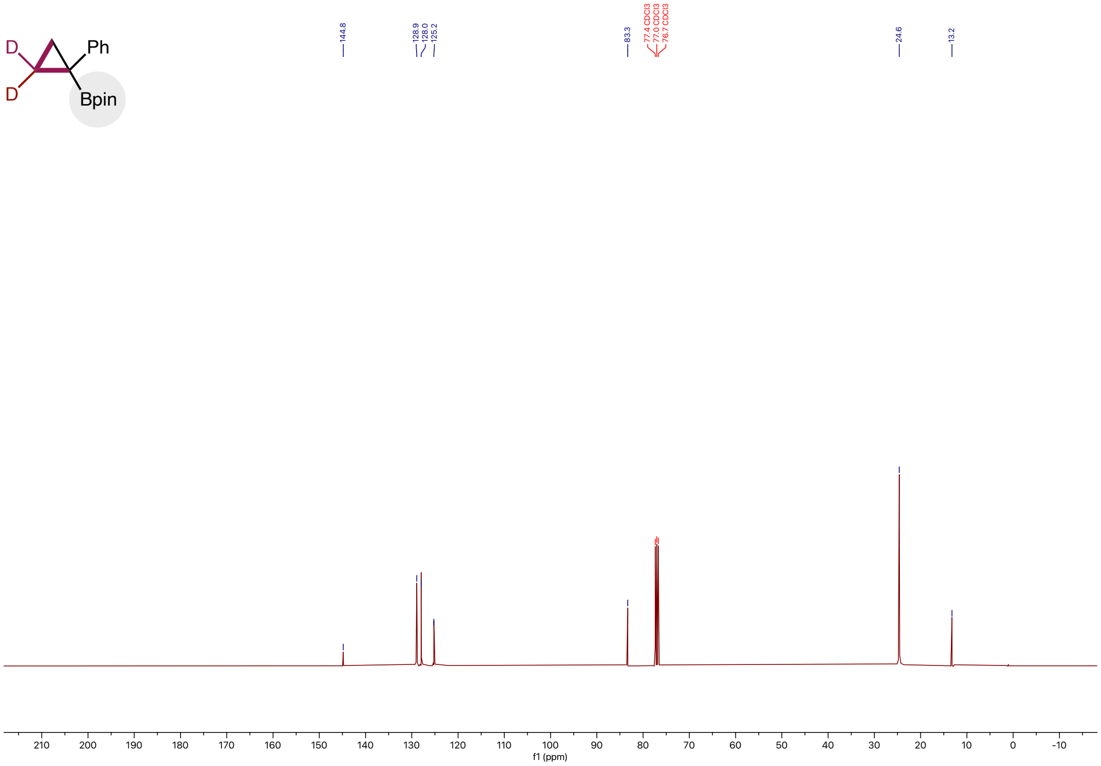

^{1}H NMR Spectrum of **25** (400 MHz, $CDCl_3$)

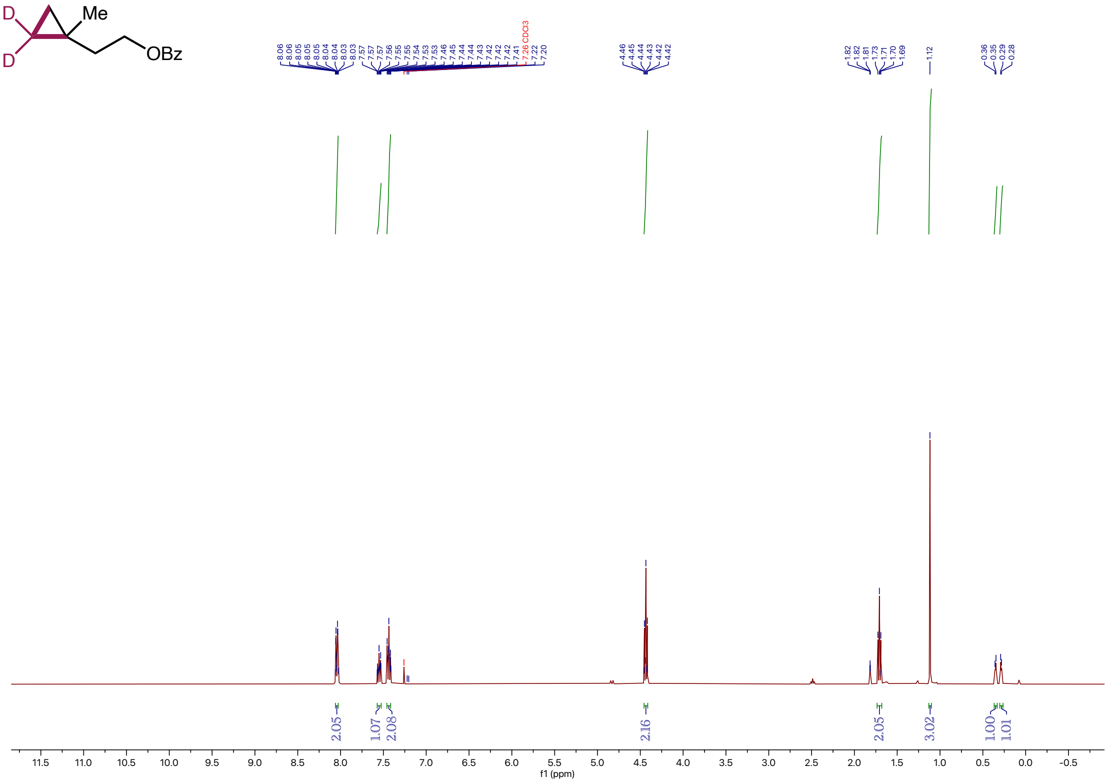

^{13}C NMR Spectrum of **25** (101 MHz, $CDCl_3$)

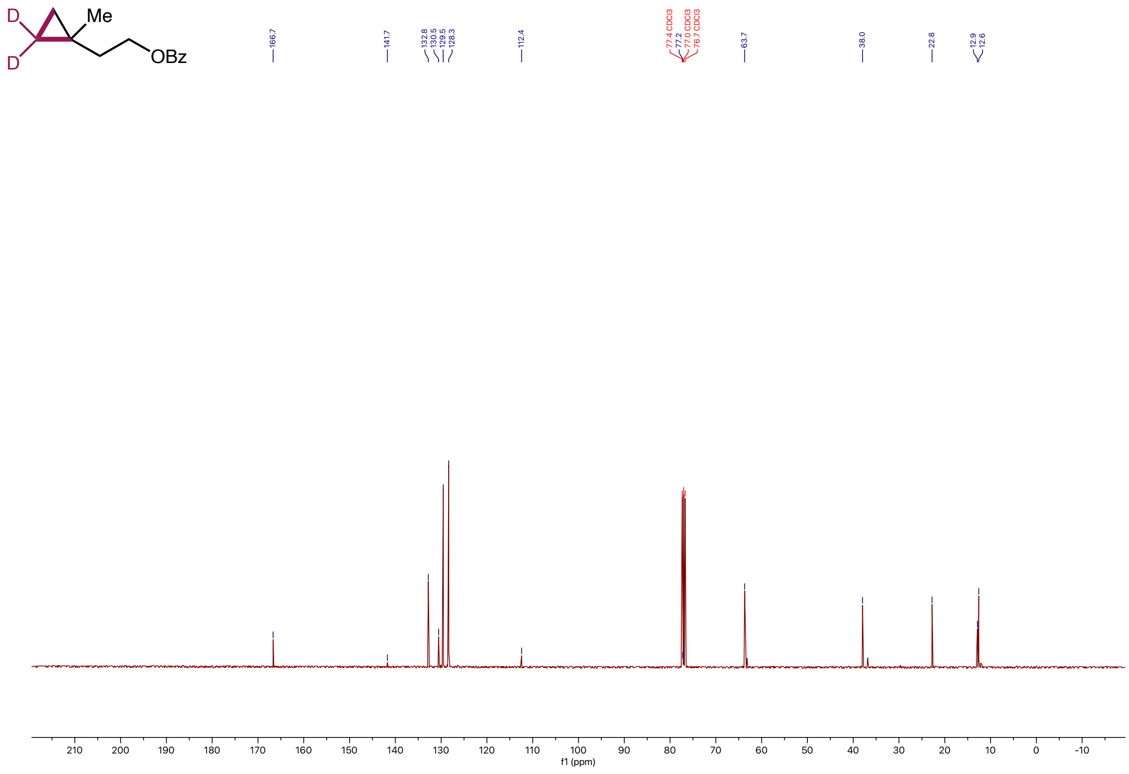

^{1}H NMR Spectrum of **26** (400 MHz, $CDCl_3$)

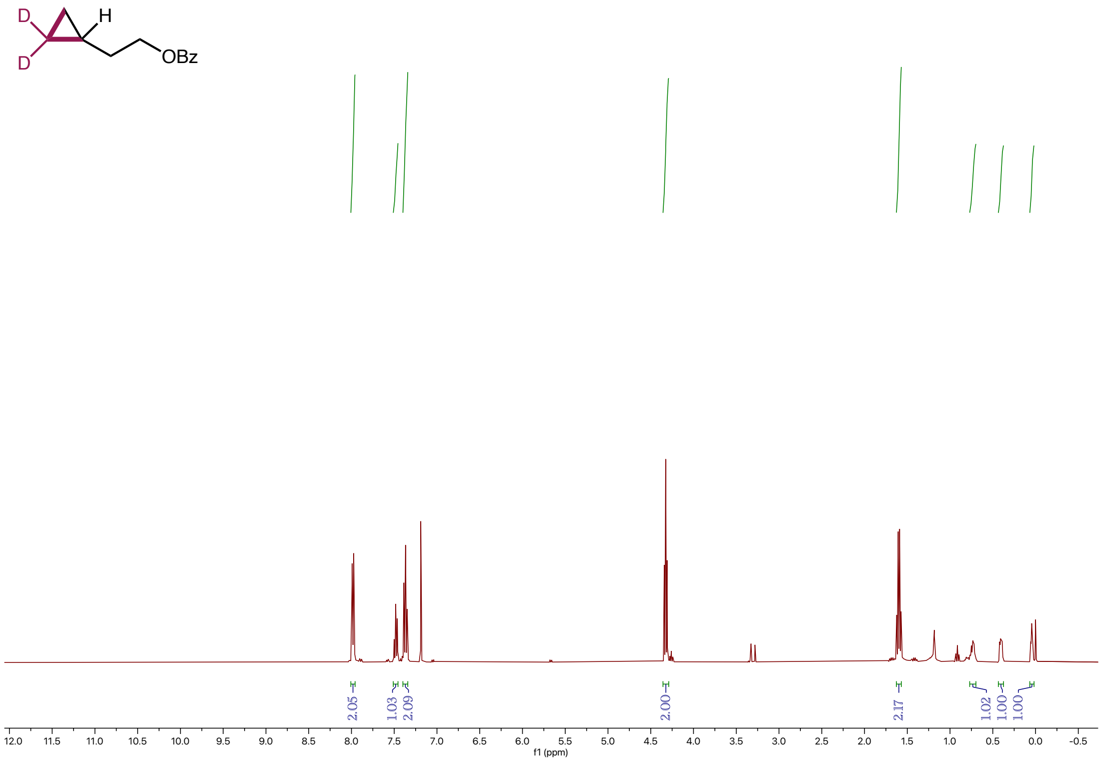

^{13}C NMR Spectrum of **26** (101 MHz, $CDCl_3$)

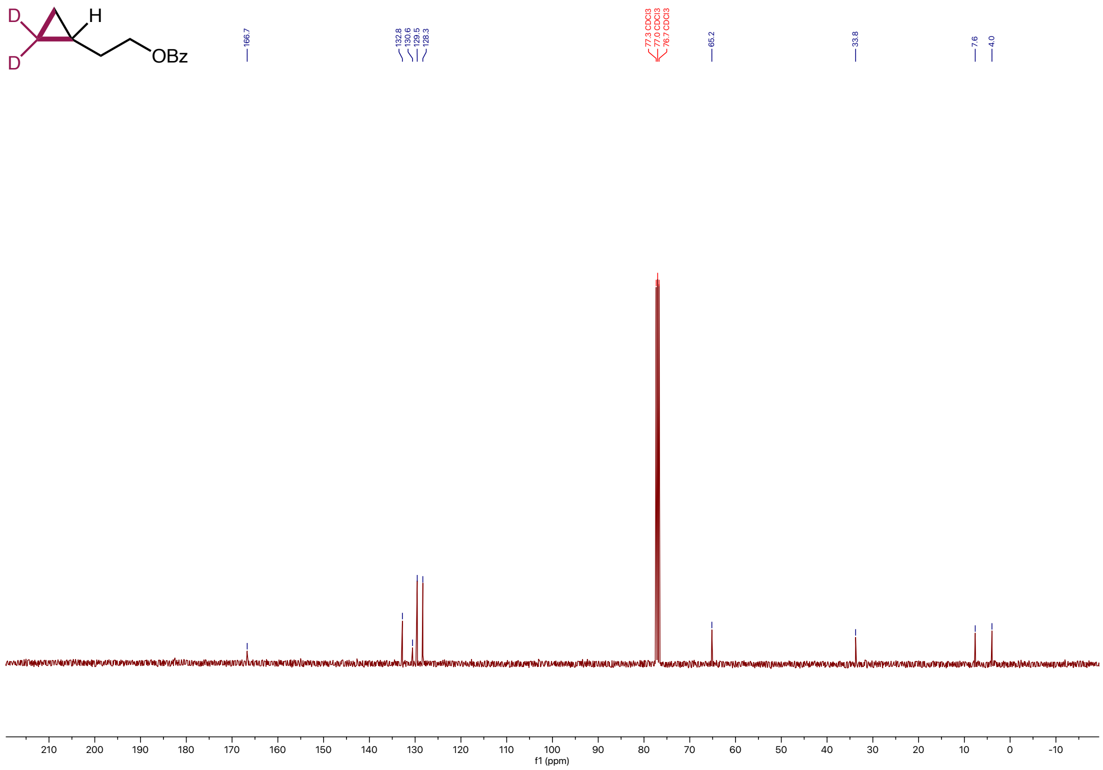

^{1}H NMR Spectrum of **27** (400 MHz, $CDCl_3$)

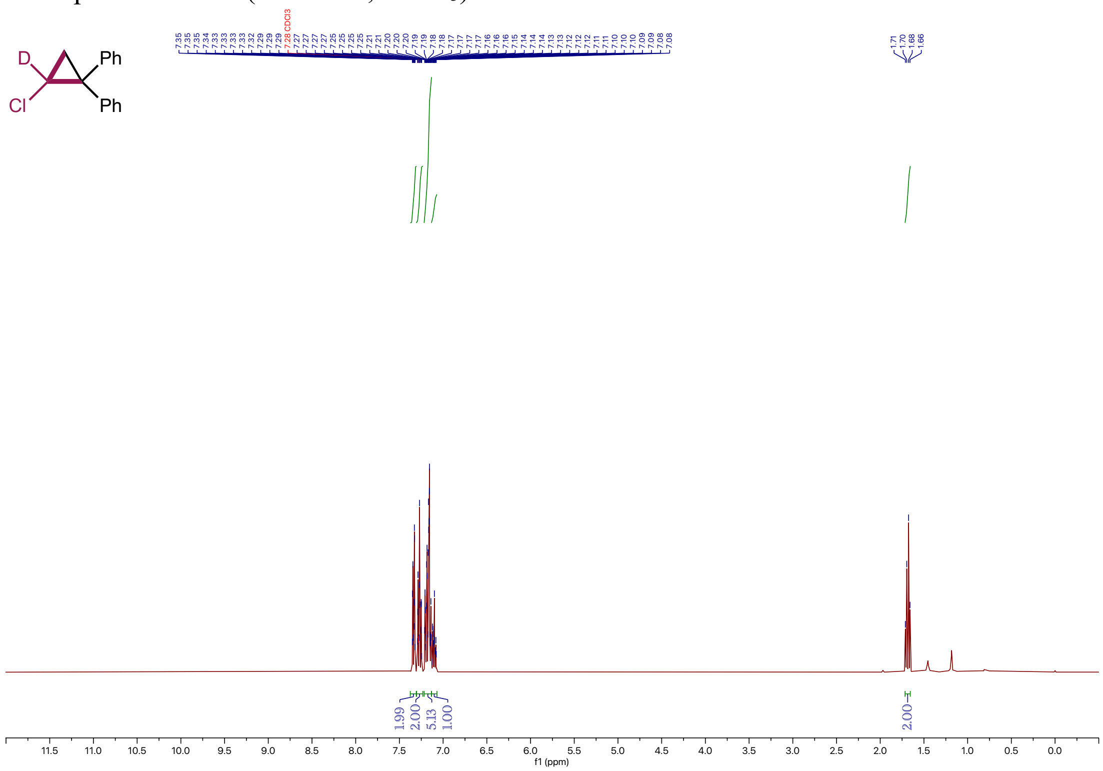

^{13}C NMR Spectrum of **27** (101 MHz, $CDCl_3$)

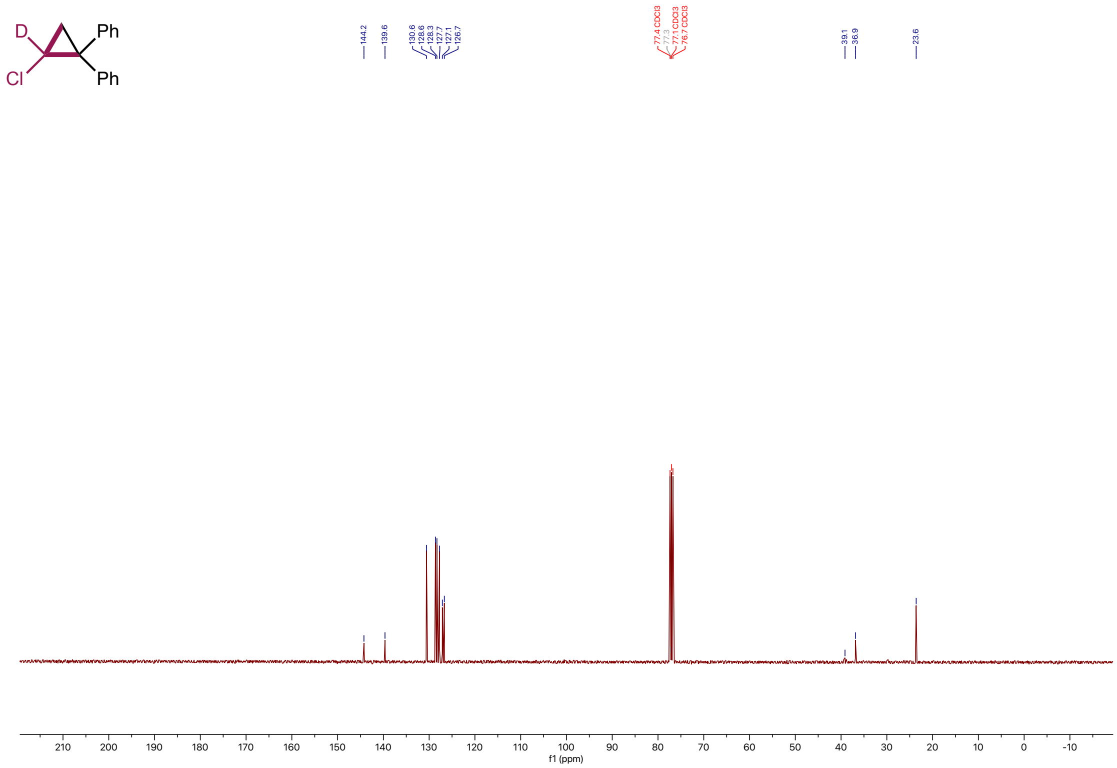

^{1}H NMR Spectrum of **28** (400 MHz, $CDCl_3$)

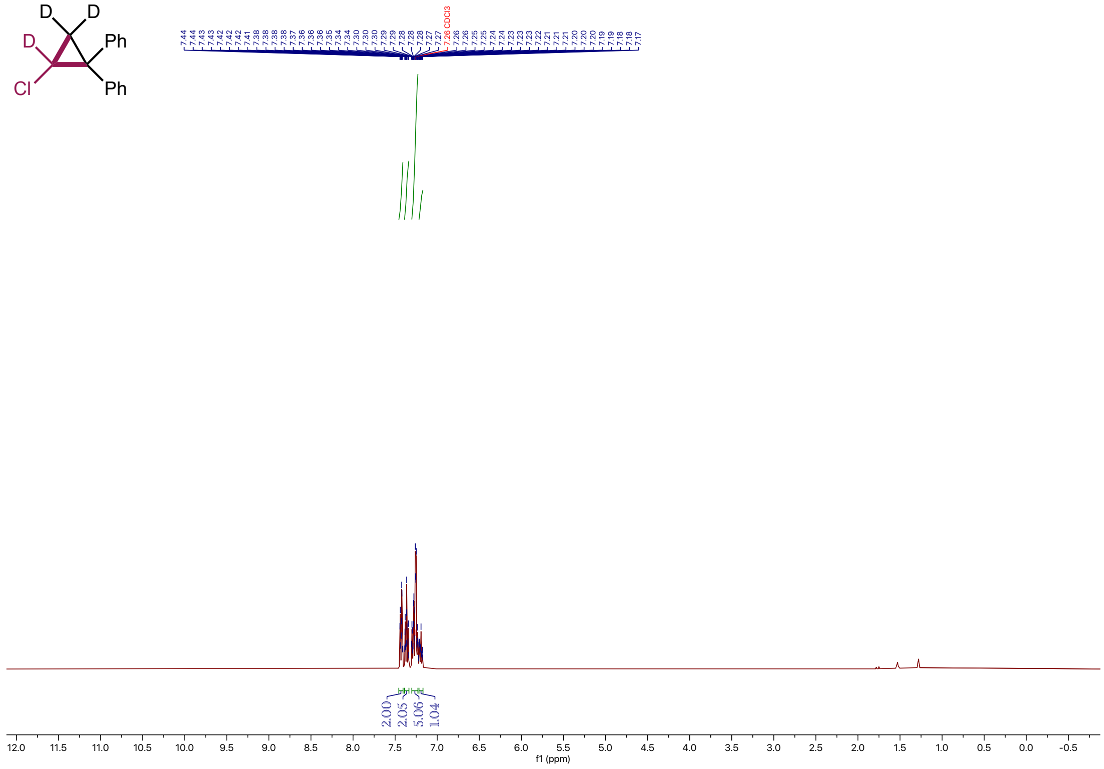

^{13}C NMR Spectrum of **28** (101 MHz, $CDCl_3$)

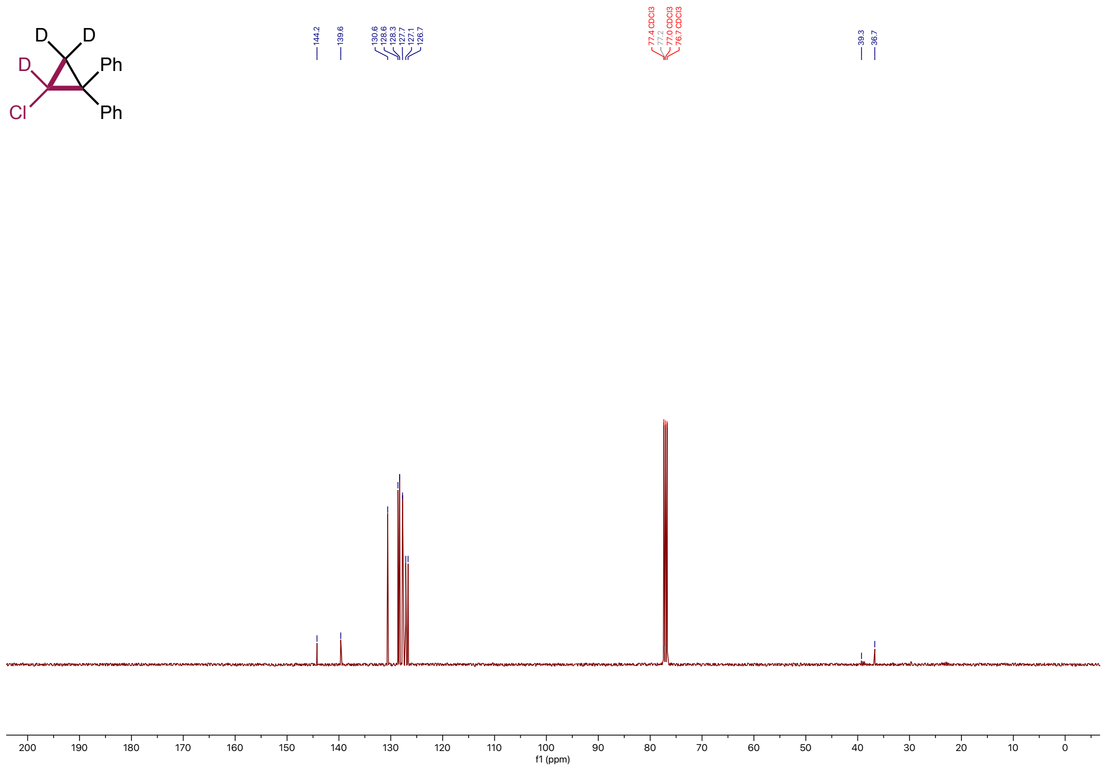

^{1}H NMR Spectrum of **29** (400 MHz, $CDCl_3$)

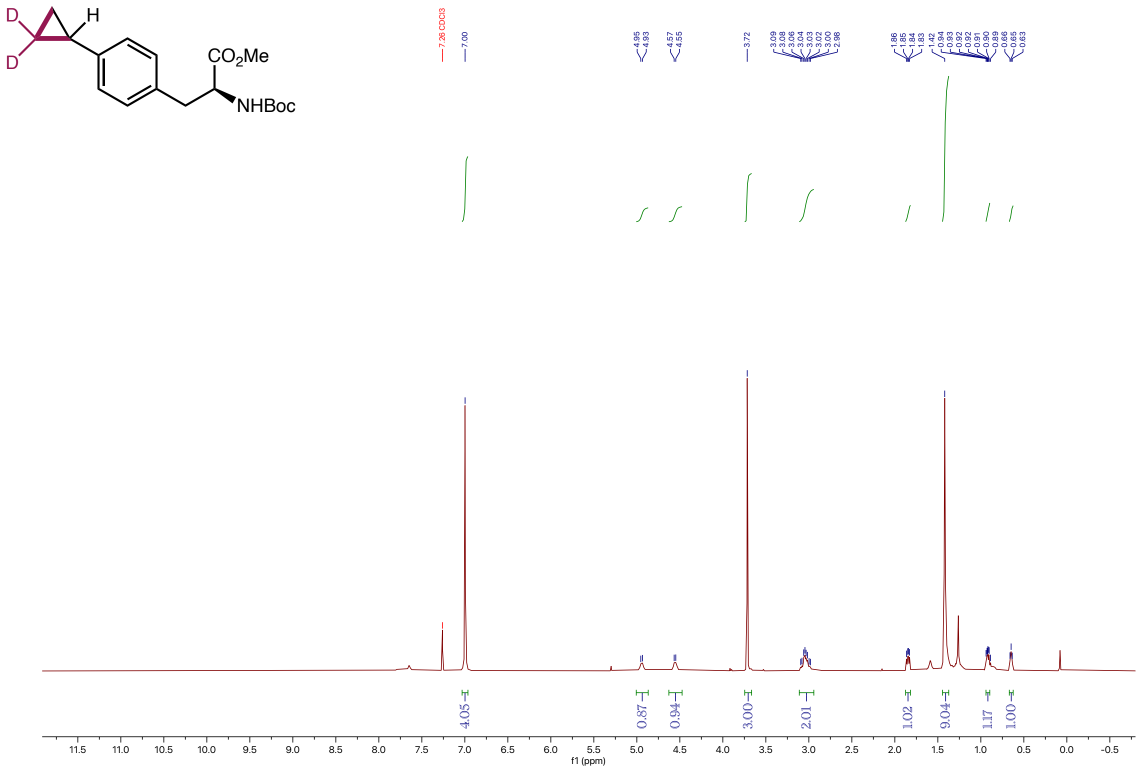

^{13}C NMR Spectrum of **29** (101 MHz, $CDCl_3$)

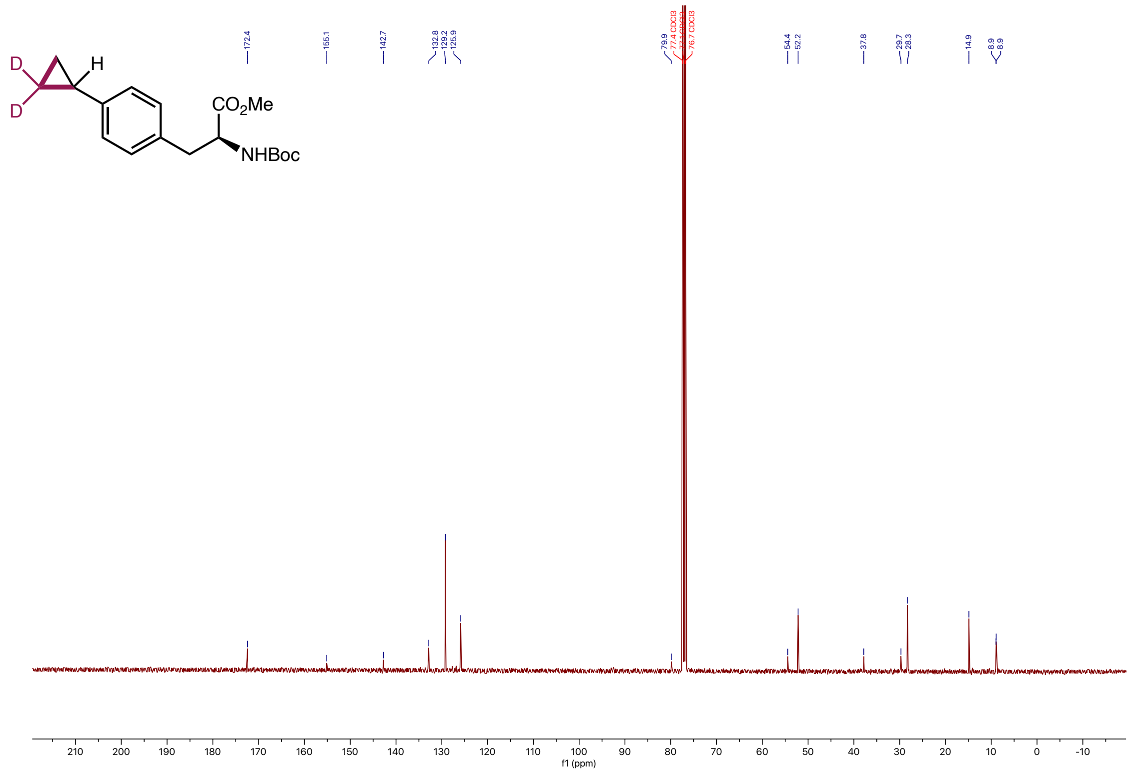

^{1}H NMR Spectrum of **30** (400 MHz, $CDCl_3$)

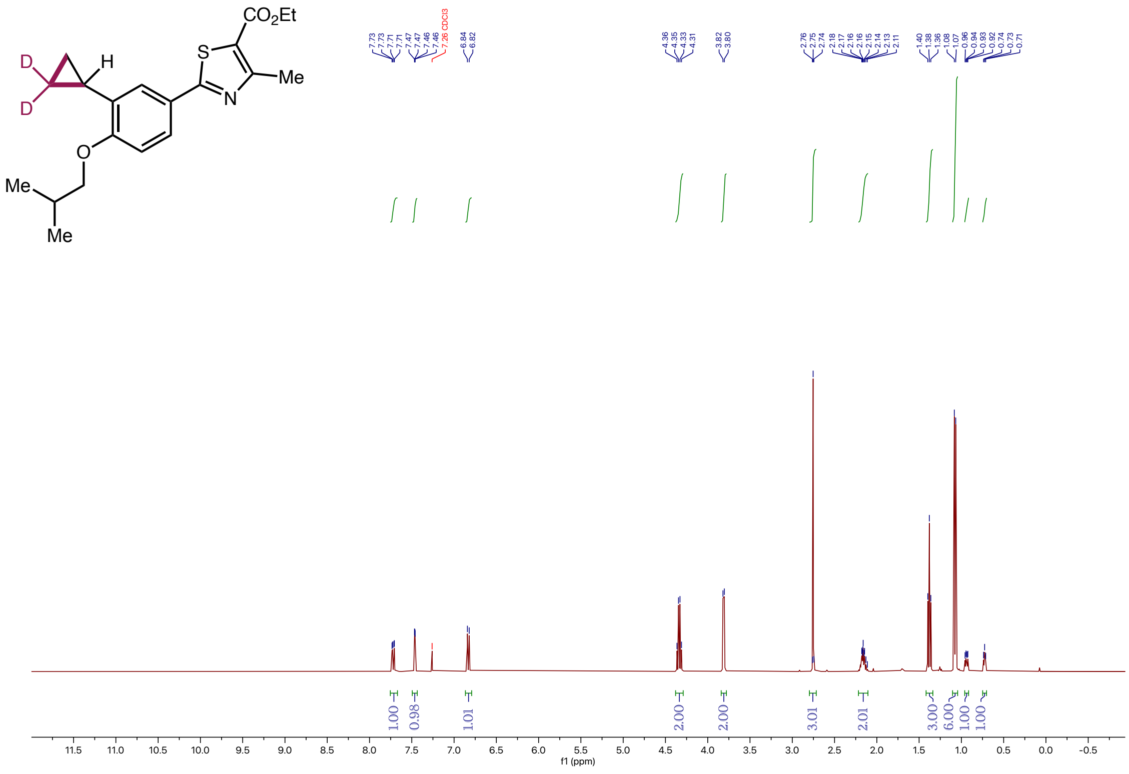

^{13}C NMR Spectrum of **30** (101 MHz, $CDCl_3$)

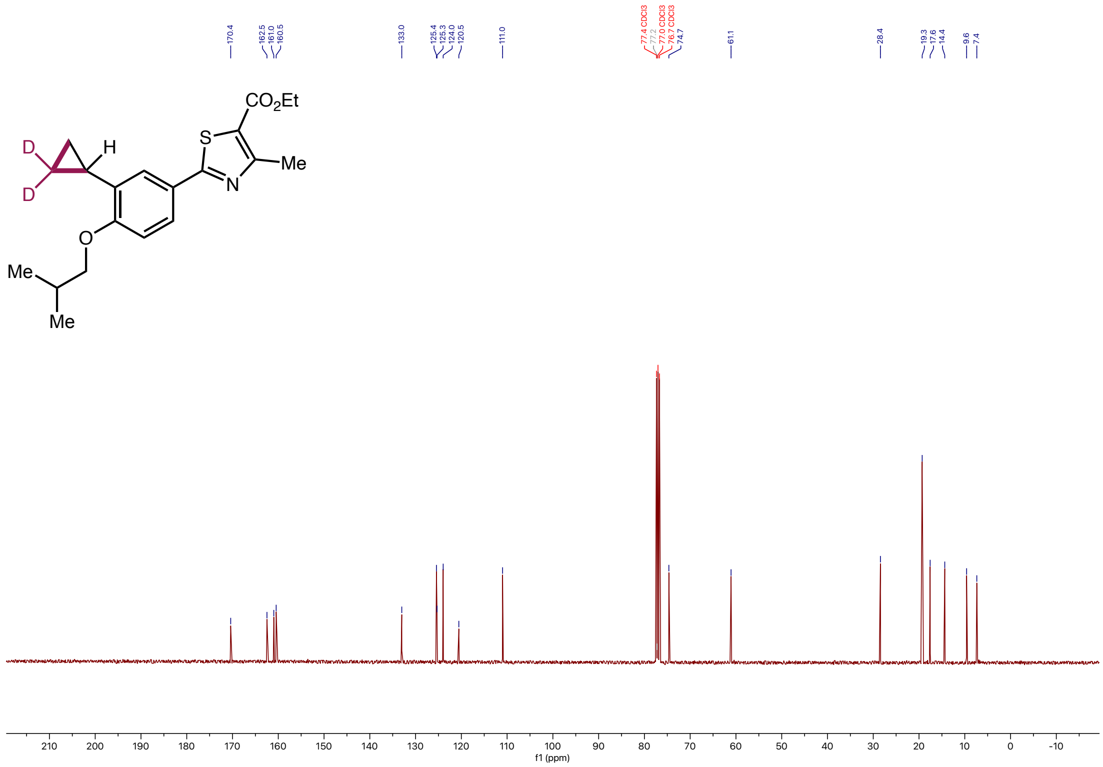

^{1}H NMR Spectrum of **31** (400 MHz, $CDCl_3$)

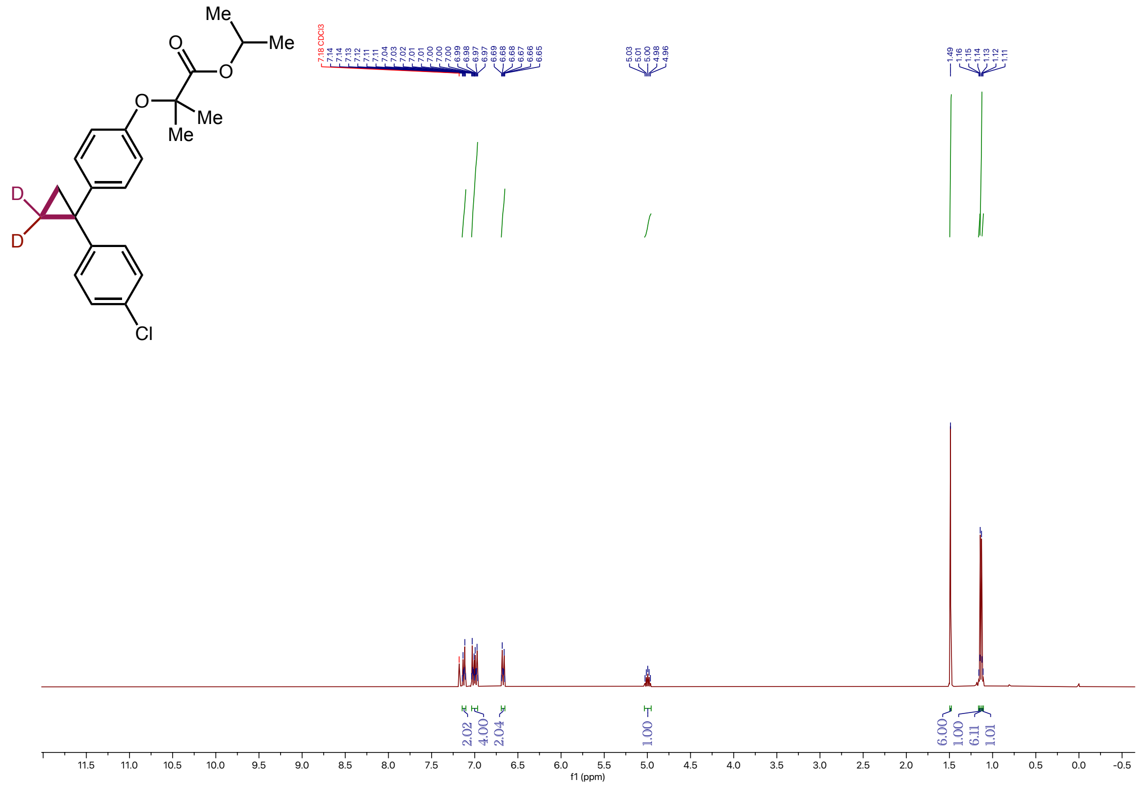

^{13}C NMR Spectrum of **31** (101 MHz, $CDCl_3$)

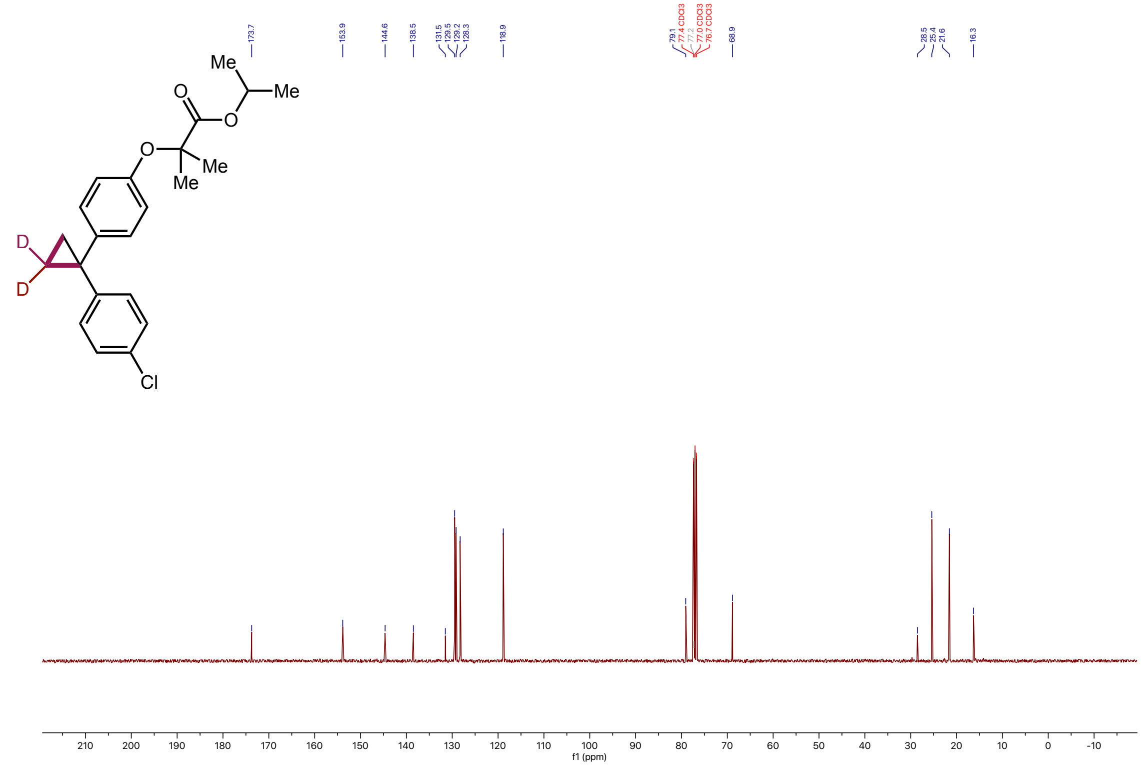

^{1}H NMR Spectrum of **32** (400 MHz, $CDCl_3$)

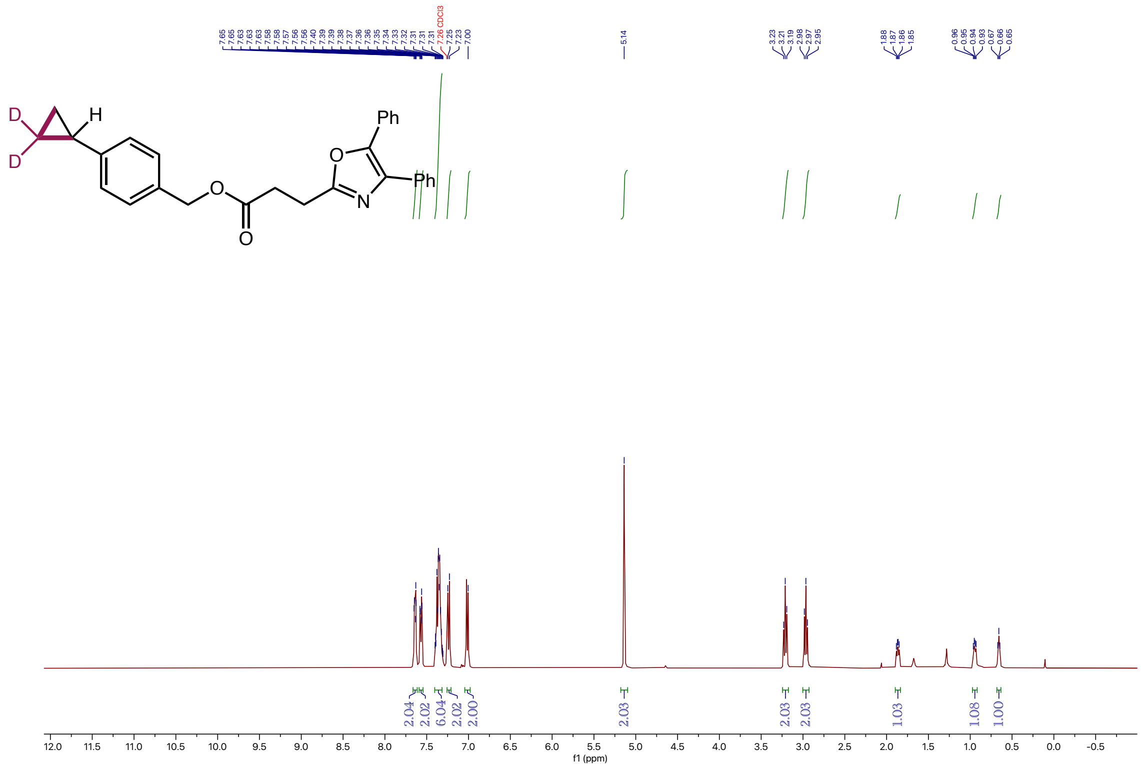

^{13}C NMR Spectrum of **32** (101 MHz, $CDCl_3$)

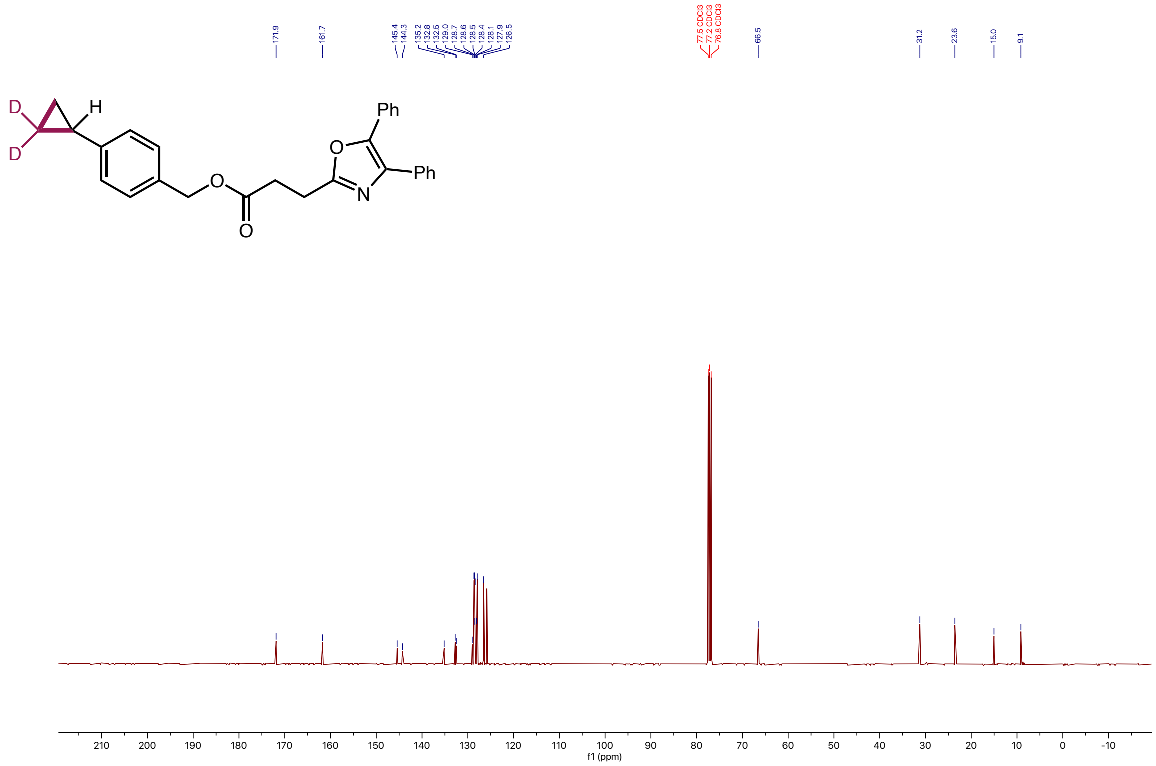

^{1}H NMR Spectrum of **33** (400 MHz, $CDCl_3$)

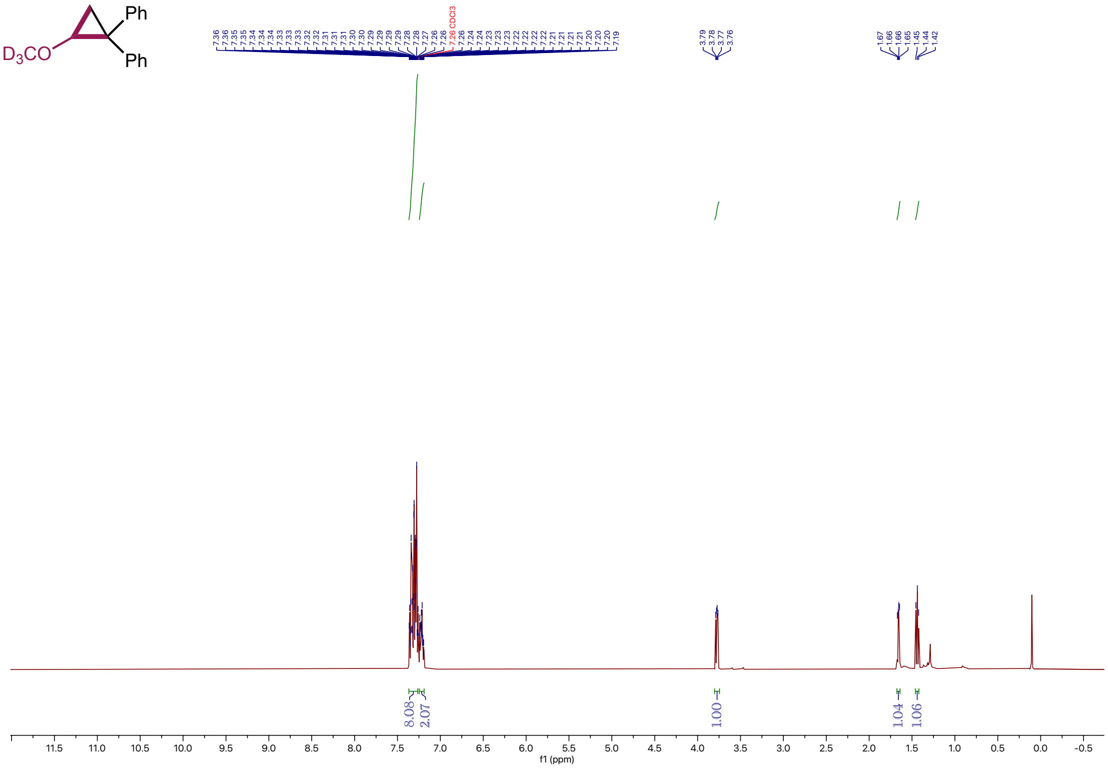

^{13}C NMR Spectrum of **33** (101 MHz, $CDCl_3$)

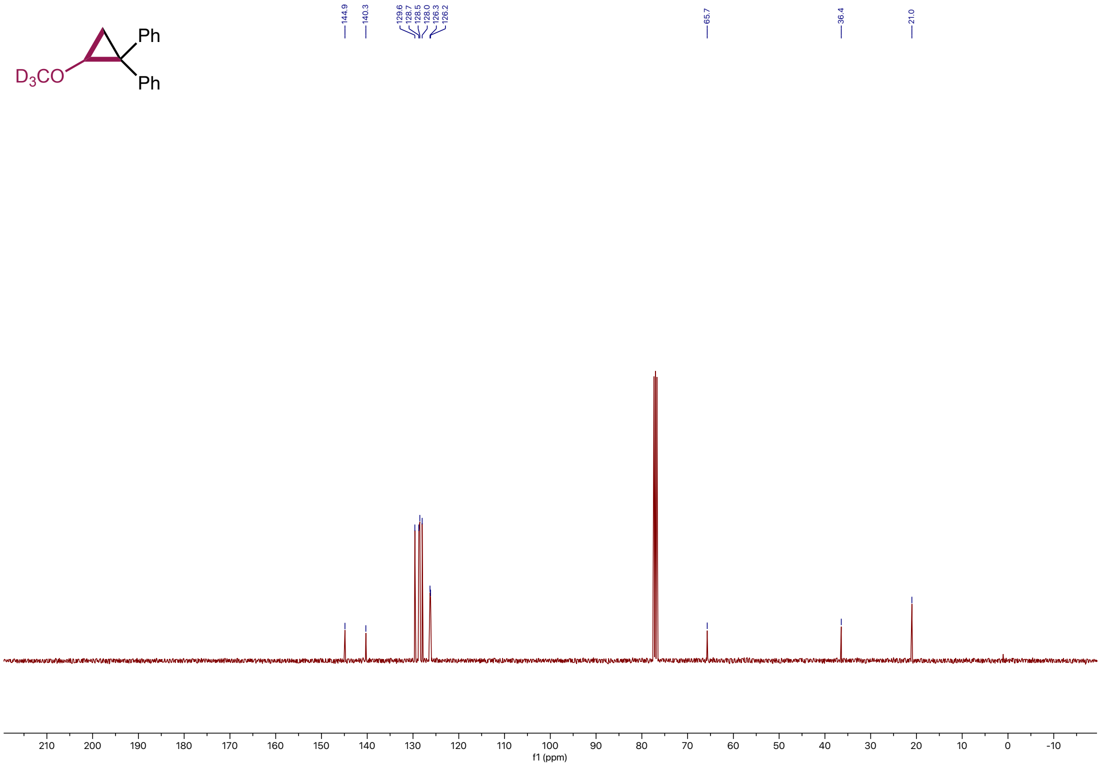

Supplement: Supplementary file 1 [file ol5c05260_si_001.pdf]
